# Decoding triancestral origins, archaic introgression, and natural selection in the Japanese population by whole-genome sequencing

**DOI:** 10.1126/sciadv.adi8419

**Published:** 2024-04-17

**Authors:** Xiaoxi Liu, Satoshi Koyama, Kohei Tomizuka, Sadaaki Takata, Yuki Ishikawa, Shuji Ito, Shunichi Kosugi, Kunihiko Suzuki, Keiko Hikino, Masaru Koido, Yoshinao Koike, Momoko Horikoshi, Takashi Gakuhari, Shiro Ikegawa, Kochi Matsuda, Yukihide Momozawa, Kaoru Ito, Yoichiro Kamatani, Chikashi Terao

**Affiliations:** ^1^ Laboratory for Statistical and Translational Genetics, RIKEN Center for Integrative Medical Sciences, Yokohama, Japan.; ^2^ Clinical Research Center, Shizuoka General Hospital, Shizuoka, Japan.; ^3^ Laboratory for Cardiovascular Genomics and Informatics, RIKEN Center for Integrative Medical Sciences, Yokohama, Japan.; ^4^ Medical and Population Genetics and Cardiovascular Disease Initiative, Broad Institute of Harvard and MIT, Boston, MA, USA.; ^5^ Cardiovascular Research Center, Massachusetts General Hospital, Boston, MA, USA.; ^6^ Laboratory for Genotyping Development, RIKEN Center for Integrative Medical Sciences, Yokohama, Japan.; ^7^ Laboratory for Bone and Joint Diseases, RIKEN Center for Medical Sciences, Tokyo, Japan.; ^8^ Department of Orthopedic Surgery, Faculty of Medicine, Shimane University, Izumo, Japan.; ^9^ Laboratory for Pharmacogenomics, RIKEN Center for Integrative Medical Sciences, Yokohama, Japan.; ^10^ Laboratory of Complex Trait Genomics, Department of Computational Biology and Medical Sciences, Graduate School of Frontier Sciences, The University of Tokyo, Tokyo, Japan.; ^11^ Department of Orthopedic Surgery, Hokkaido University Graduate School of Medicine, Sapporo, Japan.; ^12^ Laboratory for Genomics of Diabetes and Metabolism, RIKEN Center for Integrative Medical Sciences, Yokohama, Japan.; ^13^ Institute for the Study of Ancient Civilizations and Cultural Resources, College of Human and Social Sciences, Kanazawa University, Kanazawa, Japan.; ^14^ Laboratory of Genome Technology, Human Genome Center, Institute of Medical Science, The University of Tokyo, Tokyo, Japan.; ^15^ Laboratory of Clinical Genome Sequencing, Department of Computational Biology and Medical Sciences, Graduate School of Frontier Sciences, The University of Tokyo, Tokyo, Japan.; ^16^ The Department of Applied Genetics, The School of Pharmaceutical Sciences, University of Shizuoka, Shizuoka, Japan.

## Abstract

We generated Japanese Encyclopedia of Whole-Genome/Exome Sequencing Library (JEWEL), a high-depth whole-genome sequencing dataset comprising 3256 individuals from across Japan. Analysis of JEWEL revealed genetic characteristics of the Japanese population that were not discernible using microarray data. First, rare variant–based analysis revealed an unprecedented fine-scale genetic structure. Together with population genetics analysis, the present-day Japanese can be decomposed into three ancestral components. Second, we identified unreported loss-of-function (LoF) variants and observed that for specific genes, LoF variants appeared to be restricted to a more limited set of transcripts than would be expected by chance, with *PTPRD* as a notable example. Third, we identified 44 archaic segments linked to complex traits, including a Denisovan-derived segment at *NKX6-1* associated with type 2 diabetes. Most of these segments are specific to East Asians. Fourth, we identified candidate genetic loci under recent natural selection. Overall, our work provided insights into genetic characteristics of the Japanese population.

## Teaser

Whole-genome sequencing of 3256 Japanese individuals uncovered genetic characteristics of this population.

## INTRODUCTION

Whole-genome sequencing (WGS) datasets are invaluable resources for human genetic and biomedical research (*1*). Through comprehensive profiling of genetic variants, WGS data have enabled various in-depth analyses. These analyses have yielded insights into the characteristics of human genome variation (*2*), unveiled complex histories of human populations (*3*, *4*), and shed light on the processes of evolutionary adaptation and positive selection (*5*, *6*). In terms of application in genetics, WGS datasets are indispensable for imputation analysis. Large-scale WGS datasets have made it possible to construct multiethnic or population-specific reference panels (*7*, *8*). By accurately inferring ungenotyped variants from microarray data, imputation analysis effectively boosts the power of genome-wide association studies (GWASs), enables fine-mapping, and facilitates transethnic meta-analysis (*9*). Furthermore, WGS datasets provide a rich source of variants, including those that are rare, specific to certain populations, or predicted to be deleterious or loss of function (LoF) (*10*). These variants can be investigated not only for associations with various diseases but also for the effects of human knockouts, providing opportunities to identify their functional roles in both physiological and pathological processes and hence to explore the possibilities as targets for drug development (*11*, *12*). Accordingly, WGS datasets are essential to precise genetic analysis and the development of personalized medicine.

Currently, large-scale population-wide WGS data have been disproportionately represented by individuals of European descent, and substantial contributions have been made by projects such as U.K. Biobank (*13*), FinnGen (*14*), deCODE (*15*), among others. The Eurocentric imbalance in genomic data could result in unequal benefits of precision medicine and raise health disparity concerns (*16*). For example, polygenic risk scores often showed several times greater accuracy for individuals with European ancestry compared to other ancestries (*17*). Recognizing the importance of capturing the broader spectrum of human genetic variation to implement personalized medicine tailored for a specific population, concerted efforts have been made to sequence samples in more diverse ethnic groups such as in Trans-Omics for Precision Medicine and in *All of Us* project (*18*, *19*). In this context, noteworthy progress has also been made in generating WGS data from East Asian (EA) populations. Key initiatives such as GenomeAsia 100K (*20*), SG10K consortium (*21*), ChinaMap project (*22*), and Westlake BioBank for Chinese have been established (*23*). These efforts collectively uncover a wider range of genetic variants in EA populations, thereby enriching our understanding of this region’s genetic diversity. Regarding WGS data from the Japanese population, notable efforts have been made by the Tohoku Medical Megabank (ToMMo) project (*24*). Nagasaki *et al.* (*25*) conducted a WGS of 1070 Japanese individuals recruited from the northeastern area of Japan. This study identified rare genetic variants and structural variants (SVs) and generated a Japanese-specific reference panel. Subsequent sequencing efforts from ToMMo and others have continued, and summary-level allele frequencies (AFs) based on WGS of 3500 and 8300 Japanese individuals have been reported (*26*, *27*). In addition, AF data based on a continually increasing number of individuals are available in the Japanese Multi-Omics Reference Panel database and the TogoVar database (*27*, *28*). These datasets offer valuable information as a catalog of genetic variations in the Japanese population and are important for variant interpretation in the context of genetic counseling. Recently, the National Center Biobank Network has released WGS data from 9287 individuals to aim primarily for use as common control samples, further enriching the Japanese genetic data resource (*29*).

Here, we generated Japanese Encyclopedia of Whole-Genome/Exome Sequencing Library (JEWEL), a comprehensive WGS using samples from Biobank Japan (BBJ)—one of Japan’s largest biobanks and a leading entity in biobank research across Asia (note S1) (*30*, *31*). Differing from ToMMo, which is based on the general population in the northeastern area of Japan, BBJ was established as a nationwide patient-based biobank to advance the genomic medicine research (*32*). JEWEL, by sampling from diverse geographic regions, aims to better capture the genetic diversity of the Japanese. Principal components analysis (PCA) has identified a dual population structure of Japanese consisting of the main-island cluster and Ryukyu cluster, and recent studies have highlighted substantial genetic heterogeneity within main-island Japanese (*33*–*35*). Using WGS, JEWEL offers an opportunity to further explore the fine-scale population structure. In addition, extensive efforts have been made to collect and curate deep phenotypes through a review of medical records, follow-up surveys, and examinations in BBJ. These include primary and secondary disease diagnoses, longitudinal clinical test results, past medical history, family history, and survival information. As a result, JEWEL is enriched with potentially pathogenic variants associated with diseases, and detailed clinical information permits targeted examination of carriers of particular interest. In this study, we present in-depth analyses that includes a reexamination of the genetic structure using both common and rare variants, characterization of LoF variants and human knockouts, and identification of archaic segments likely introgressed from Neanderthals or Denisovans. Last, we attempted to identify genetic loci potentially targeted by selection in the Japanese population.

## RESULTS

### Characteristics of the JEWEL WGS dataset

**Fig. 1.**
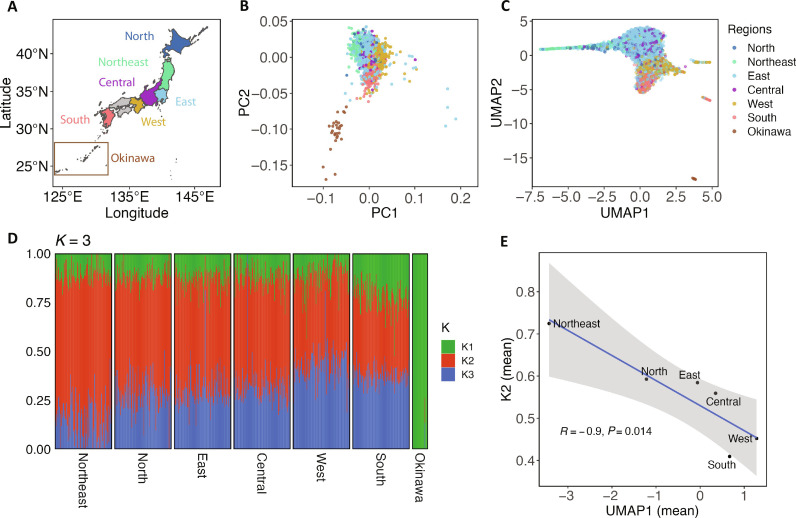
Fine-scale genetic structure of the modern Japanese and its three ancestry origins. (**A**) Geographic regions in Japan from which the samples were recruited are described. These regions include the Japan archipelago, commonly known as Hondo, and the Ryukyu archipelago, which is termed as Okinawa in this study. The number of individuals from each region is provided in table S1. (**B**) PCA analysis based on common variants with a minor AF (MAF) ≥ 0.01. Individuals are colored according to their recruitment regions. (**C**) Rare variant–based PCA-UMAP analysis (0.001 ≤ MAF < 0.01) is displayed. (**D**) ADMIXTURE analysis with *K* set to 3. For regions other than Okinawa, 100 individuals were randomly selected and plotted. All 28 individuals from Okinawa were included in the plot. K1 represents Okinawa, while K2 and K3 are the highest in the Northeast and West, respectively. (**E**) UMAP1 is negatively correlated with the fraction of K2 ancestry. The correlations between each combination of UMAP and *K* are presented in fig. S5.

A total of 3256 individuals, enrolled from medical institutes in seven geographic regions across Japan, were sequenced to generate JEWEL. These regions include Hokkaido, Tohoku, Kanto, Chubu, Kansai, Kyushu, and Okinawa, which are hereafter referred to as North, Northeast, East, Central, West, South, and Okinawa (see Materials and Methods and Fig. 1A). All regions except for Okinawa are located on the main islands of the Japanese Archipelago, commonly known as Hondo, while the term Okinawa in this study indicates the Ryukyu islands. The relative sample size proportionally reflects the population sizes of these regions in Japan (table S1). Sequencing was performed according to standard Illumina protocols, and an average WGS coverage depth of 25.6× was achieved. Variant calling was conducted according to established Genome Analysis Toolkit (GATK) best practices (see Materials and Methods and note S2 for details). The final dataset consisted of 45,586,919 single-nucleotide variants and 9,113,420 insertions or deletions (indels) from 23 chromosomes. We observed that 61 and 40% of variants were not registered in the Genome Aggregation Database (gnomAD) and ToMMo, respectively (*26*, *36*) (table S2); 15,410,953 (32.7%) variants were only observed in JEWEL. Compared to microarray genotyping data, a high genotype concordance rate of 99.971% was obtained (see Materials and Methods). Using 42,389,421 biallelic autosomal single-nucleotide variants, we estimated the ratio of transition to transversion (Ti/Tv) to be 2.11, which was in line with recent large-scale WGS analyses (*21*, *22*) (tables S2 and S3). These results confirmed that JEWEL dataset is of high quality in various aspects, allowing for a deeper analysis of the genetic characteristics of this population.

### Triancestral origins of the Japanese population

We first conducted a conventional PCA based on 184,036 independent pruned common variants (see Materials and Methods). Consistent with previous studies, the analysis replicated the classic “dual-cluster” structure consisting of Okinawa and the Hondo clusters (Fig. 1B) (*33*, *35*, *37*). We hypothesized that rare variants might be more informative in revealing the population structure, and we conducted a PCA–Uniform Manifold Approximation and Projection (PCA-UMAP) analysis, which exclusively used 1,835,116 independent pruned rare variants (see Materials and Methods). The analysis uncovered an unprecedentedly fine structure of the Japanese population (Fig. 1C). This structure, resembling a “hummingbird,” not only recapitulated patterns obtained from PCA based on common variants but also highlighted several notable features. Specifically, we observed (i) a clearer separation among subregions of Hondo and a clearer distinction of Okinawa cluster from Hondo cluster, (ii) Northeast individuals clustered in a thin, narrow area, and (iii) additional subclusters of individuals from West and South (figs. S1 and S2 and note S3).

To gain a deeper insight into the population structure, we performed an unsupervised ADMIXTURE analysis based on common variants (see Materials and Methods and note S4). To determine the optimal *K* value, we used Structure Selector, a method demonstrated to exhibit superior performance compared to other estimators (*38*). In this analysis, all four metrics support the *K* value of three as the optimal number of ancestral components (fig. S3). In addition, we used badMIXTURE to evaluate the goodness of fit and observed no systematic pattern of large residuals, indicating an overall good fit at *K* = 3 (fig. S4) (*39*). Therefore, our data suggested that the Japanese population could be best modeled by admixtures of three ancestral components (hereafter K1 to K3). K1 to K3 were the highest in Okinawa, Northeast, and West, respectively (Fig. 1D and table S4). K1 (Okinawa) component maintains a relatively stable fraction of around 12% in Hondo subgroups, except for South (which is a region adjacent to Okinawa), with a higher proportion of 22%. K2 (Northeast) and K3 (West) components showed a cline from West to East. We also conducted the ADMIXTURE analysis using both common and rare variants and observed consistent results, with additional detail from Okinawa (note S4).

We observed significant correlations between *K* values and PCA-UMAP values, despite the former obtained from the analysis of common variants and the latter from rare variant analysis. This finding seemed to offer additional support for *K* = 3. Specifically, UMAP1 is significantly correlated with K2/K3 (Pearson coefficient = −0.69 with K2 and 0.60 with K3, *P* < 2.2 × 10^−16^ for both). This correlation pattern can also be clearly visualized by aggregating samples according to their respective regions (Fig. 1E and fig. S5). We additionally analyzed *K* values in the context of geography and found that the proportions of Okinawa (K1) and Northeast (K2) ancestries are correlated with geographic longitude. In contrast, the correlation with West (K3) is less pronounced and not statistically significant (fig. S6).

We attempted to gain hints about the potential ancestral origins of K1 to K3. Previous studies have suggested that Japanese carry Jomon and EA ancestry (represented by Han Chinese) (*34*, *40*). Recently, the presence of Northeast Asian (NEA) ancestry has been proposed on the basis of analyses of ancient genomes (*41*, *42*). In this context, we analyzed our data together with modern and ancient genetic data of Jomon, EA, and NEA. Using *f*_4_ ratio statistic, we estimated that Okinawa had the highest Jomon ancestry (28.5%), followed by Northeast (18.9%), and the lowest in West (13.4%) (see Materials and Methods and table S5). These results align with prior studies demonstrating a high genetic affinity between Jomon and Okinawa people (*43*, *44*). Next, on the basis of outgroup *f*_3_ statistic, we observed that individuals from West had the highest shared genetic drift with Han Chinese (table S6). We then used *f*_4_ statistic in the form of *f*_4_ (Mbuti, ancient genome; Northeast, West) to evaluate differential genetic affinities between Northeast and West, in relation to ancient genomes reported from China, Korea, and Japan (*41*, *44*–*47*). Our results indicated a significantly closer relationship between West and ancient Chinese groups around the Yellow River (YR) or upper YR region, specifically in the Middle Neolithic (MN) and Late Neolithic periods (table S7). In contrast, individuals of Northeast showed significantly higher genetic affinities with Jomon and ancient Japanese genome from Miyako Island in Okinawa (which had a high Jomon proportion) and ancient Koreans from the Three Kingdoms (TK) period (Korea-TK_2) (fourth to fifth century CE) (table S7). These results align with reports indicating that ancient Japanese in the Yayoi period and certain ancient Korean groups had a high proportion of Jomon ancestry (*42*, *47*).

We subsequently used qpAdm to estimate contributions of NEA, EA, and Jomon ancestries in each subgroup, following the approach described in prior studies (*41*, *48*) (see Materials and Methods). For this analysis, the Chinese Han was designated as representative of EA, while China_WLR_BA_o and China_HMMH_MN were grouped to represent NEA. The results revealed a generally good fit of the tripartite model to our dataset (table S8). The proportions and trends of Jomon ancestry estimated through qpAdm align with the findings from the *f*_4_ ratio test, revealing the highest proportion in Okinawa (25%) and the lowest in the West (7.5%). Likely because of the low Jomon ancestry in West, we observed that EA ancestry is the highest in South rather than West. However, the fitting of this model for Northeast was rejected, indicated by an extreme *P* value (*P* = 6.5 × 10^−4^). Exploring additional models, we found that Northeast could be alternatively modeled as a two-way admixture of Korea-TK_2 (68%) and Han (32%) (tables S8 and S9). Notably, among Hondo groups, Northeast showed the highest proportion of Korea-TK_2. For West, the initial three-way model that includes NEA, EA, and Jomon showed a better fit, as indicated by a lower chi-square value (9.14 compared to 11.8). Furthermore, the two-way admixture modeling involving combinations of Jomon, EA, and NEA proved to be unsuccessful (table S9). These multiple lines of evidence suggest that K1 and K3 may be linked to Jomon and EA ancestries. Although less clear, the ancestral origins of K2 could potentially be connected to ancient populations in Japan and the Korean Peninsula, such as Korea-TK_2.

Motivated by the above findings, we investigated whether this triancestral framework could offer insights into the likely origins of Japanese founder mutations. We focused on two high-frequency pathogenic mutations associated with hereditary breast cancer among Japanese patients—the *BRCA1* Leu63Ter and the *BRCA2* c.5576_5579delTTAA frameshift mutation. The former is specific to the Japanese population and has a significantly higher frequency in Eastern Japan than in Western Japan (*49*). In contrast, the latter has a high frequency in Western Japan and has been reported in other Asian populations, including Chinese (*50*) and Korean (*51*). Plotting *BRCA1* Leu63Ter carriers in the PCA-UMAP showed that this mutation predominantly occurred in individuals with likely Northeastern ancestry, and its occurrence is significantly associated with UMAP1 (*P* = 9.04 × 10^−6^, logistic regression) (fig. S7). This pattern was not apparent when considering enrollment locations, as most carriers were recruited from East (seven of nine carriers were recruited from East, with the remaining two from North and Northeast). On the other hand, the *BRCA2* c.5576_5579delTTAA mutation was predominantly observed in individuals of West ancestry (fig. S7). Our data align with a recent study based on ~100,000 Japanese samples, showing that *BRCA1* Leu63Ter has the highest frequency in Northeast, while the *BRCA2* frameshift mutation is most frequent in West (*52*). Despite our much smaller sample size, the rare variant–based fine structure sheds insights into the likely origins of the two mutations in Japanese. The data suggested that the *BRCA1* Leu63Ter mutation likely originated in Northeast ancestry and spread to other regions. Since Japanese in West had a higher genetic affinity with Han Chinese, we speculate that this mutation may have been introduced to Japan from continental Asia. In addition, we explored whether *K* values are associated with quantitative phenotypes in JEWEL individuals based on linear regression. We found significant associations, particularly for total cholesterol (*P* = 2.69 × 10^−13^) and prothrombin time (PT; *P* = 1.33 × 10^−12^) with K1. Comparable *P* values of these traits with K2 were also observed (table S10).

### LoF variants and human knockouts

**Fig. 2.**
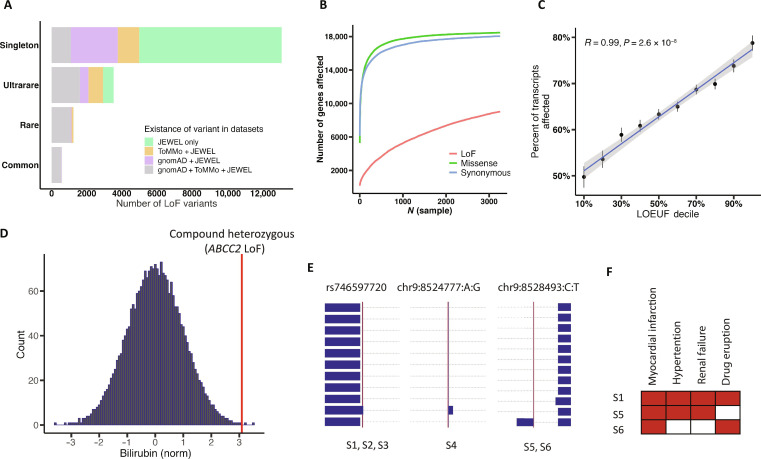
LoF variants and human knockout in the JEWEL dataset. (**A**) Number of known and unregistered LoF variants compared with gnomAD database (v2.1.1) and ToMMo (4.7K). Variants are categorized into four AF bins. Common: MAF > 1%; rare, MAF < 1% and MAF ≥ 0.01%; ultrarare: minor allele count > 1 and MAF < 0.01%; singleton. (**B**) Cumulative number of genes affected by LoF, missense, and synonymous variants. (**C**) The average percentage of transcripts affected by LoF variants, categorized by the genes’ LOEUF deciles. Genes that are highly intolerant to functional variation, as indicated by lower LOEUF deciles, have fewer affected transcripts compared to genes that are more tolerant. Error bars are included to indicate SEs. (**D**) The histogram of normalized total bilirubin levels among individuals in the JEWEL cohort. The red line highlights an individual with compound heterozygous LoF variants in the *ABCC2* gene, ranking third in the whole JEWEL dataset. This elevated level of total bilirubin is consistent with the clinical phenotype of Dubin-Johnson syndrome, which is caused by the inactivation of the *ABCC2* gene. (**E**) The plot presents data on six individuals carrying LoF variants in the *PTPRD* gene. The identifier for each LoF variant, either rsID or variant ID, is displayed at the top. Blue boxes represent exons for different transcripts, while the red lines mark the locations of these LoFs. Individual IDs carrying the LoF variants are indicated at the bottom. A zoomed-out perspective of the plot is presented in fig. S9. (**F**) The shared phenotypes among three *PTPRD* LoF carriers for whom comprehensive clinical data are available (S1, S5, and S6), with the names of the phenotypes provided for reference.

JEWEL dataset allowed us to explore potentially clinically important protein-coding variants in Japan. In our analysis, we identified 18,481 LoF variants in 9045 genes, including 9780 LoF variants not registered in gnomAD or ToMMo (4.7K), with a substantial proportion of these being rare (Fig. 2A and table S11). These LoF variants are defined as variants that may cause premature stop codons (stop-gained), small-sized indels that shift the coding sequence (frameshift), or variants that change two immediately adjunct nucleotides to the splicing sites (splicing variants). Furthermore, we classified 177,112 synonymous variants and 306,923 missense variants, which affected 18,651 and 19,103 genes, respectively (Fig. 2B). Examination of LoF variants together with carriers’ UMAP values identified 32 and 37 LoF variants, whose frequencies were significantly associated with UMAP1 and UMAP2 (false discovery rate < 5%), respectively (see Materials and Methods and table S12). We noticed that individuals from Northeast had the lowest average number of singleton coding variants compared to those from other regions (table S13). Since the sample size of Northeast is smaller than that in other Hondo regions, we conducted a random resampling analysis and confirmed that this observation is likely not attributable to sample size (table S14). We speculate that other factors, such as demographic history, especially population expansion, may be influencing this observation. Despite regional differences, the ratio between singleton missense and singleton synonymous variants (dN/dS) across regions was consistently close to 2, which is an observed ratio of de novo missense and synonymous variants reported in an in vivo study (*53*). Furthermore, consistent with observations in another report, this ratio negatively correlates with the AF, suggesting that many rare missense variants might be deleterious but remain in the gene pool (*54*). To further test this idea, we calculated the missense risk score by integrating annotations from 30 different annotation tools (see Materials and Methods). We observed that the missense risk score increased as the AF decreased (*P* < 2.2 × 10^−16^, Pearson correlation test). On average, singletons exhibited the highest risk scores (table S15). On the basis of the data above, missense variants that are rare in the general population could be prioritized for disease association analysis. This approach to prioritization could narrow down potential candidates, thereby increasing the likelihood of identifying a meaningful clinical connection.

JEWEL allowed us to further assess the potential applicability of LoF observed/expected upper-bound fraction (LOEUF) scores in the Japanese population. The LOEUF score was introduced as a metric to quantify a gene’s tolerance to LoF variants, based on observed and expected counts of LoF variants in the gnomAD project (*36*). Given that individuals with EA ancestry constituted 7% of the gnomAD dataset, we are interested in testing whether LOEUF score is applicable to JEWEL. We observed that genes in the lowest LOEUF decile bin (indicating the highest intolerance to LoF variants) were least affected by LoFs (fig. S8). This supports the utility of LOEUF scores in stratifying genes highly intolerant to LoF variants. However, a discrepancy was found in the number of genes affected by LoF variants in top decile bins (fig. S8). Furthermore, we observed that the fraction of transcripts affected by LoF variants showed a significant positive correlation with LOEUF bins (Fig. 2C). Overall, these results support the generalizability of LOEUF score while acknowledging that there might be room for improvement in relation to LoF-tolerant genes.

Pathogenic variants and human knockouts are highly valuable for clinical research and drug development and may reveal human genotype-phenotype connections. We identified 371 ClinVar-registered pathogenic variants and 1723 unreported LoF variants in genes harboring pathogenic variants in ClinVar (note S5). We searched for human knockouts, defined as homozygotes or compound heterozygotes for LoF variants. Inspection of annotations and manual curation identified 23 human knockouts that are likely to be clinically relevant. We noted a carrier of compound heterozygous LoF variants in the *ABCC2* gene (see Materials and Methods and table S16). The LoF of this gene is known to cause Dubin-Johnson syndrome, an autosomal recessive liver disease related to hyperbilirubinemia (*55*, *56*). The syndrome is typically benign, and patients exhibit an increase in total bilirubin in the blood, leading to chronic jaundice. We obtained clinical history records and blood test results for this individual and confirmed the diagnosis of Dubin-Johnson syndrome and the clinical manifestation of hyperbilirubinemia (Fig. 2D). Furthermore, two of three individuals with homozygous LoF variants in *GJB2*, a gene associated with nonsyndromic sensorineural hearing loss, were confirmed to have hearing loss (*57*). These examples demonstrate that we can use JEWEL to identify likely underlying pathogenic variants responsible for diseases and to mine potentially clinically relevant genotype-phenotype connections.

In addition to conventional human knockout analyses presented above, we leveraged rich phenotyping data in JEWEL to examine individuals with heterozygous LoF variants in genes considered highly intolerant to LoF variants, as indicated by LOEUF scores. Focusing on genes that have multiple LoF variants, we identified six individuals with LoF variants in *PTPRD*, one of the top-ranked LOEUF genes (LOEUF = 0.11, rank = 271 among 19,704 genes), which encodes a receptor-like protein tyrosine phosphatase (Fig. 2E) (*58*). Detailed clinical information was obtained for three of the six individuals, who presented with several shared phenotypes, including myocardial infarction, kidney failure, hypertension, and drug eruption (Fig. 2F and table S17). The *PTPRD* gene has 13 transcripts with most exons being identical and shared among multiple transcripts. However, only two transcripts were affected by LoF variants, which is significantly fewer than would be expected by chance (*P* = 0.005, permutation test; see Materials and Methods, Fig. 2E, and fig. S9). We searched the literature for reported human knockout of *PTPRD*. A case report described a child carrying homozygous microdeletion of *PTPRD*, which was suspected to be associated with intellectual disability, trigonocephaly, and hearing loss (*59*). In addition, *Ptprd* knockout mice exhibit preweaning lethality with an incomplete penetrance (*60*). Given these data and the low LOEUF score, disruption of PTPRD protein might be highly deleterious. However, if LoFs affect only a limited number of transcripts or if the affected transcripts are of lesser functional importance, then the consequences might be more tolerable. Further genome-wide scanning identified additional genes where LoF variants occurred in a restricted set of transcripts, including two more PTPR family genes, both of which are in the lowest LOEUF bin, *PTPRS* (LOEUF = 0.25, *P* = 0.002) and *PTPRM* (LOEUF = 0.23, *P* = 0.009) (table S18). The results suggest that phenotypic impacts of certain LoFs may be mitigated, even in genes that are generally intolerant to LoF. However, other factors such as nonrandom sampling or inaccurate annotation of LoF transcripts should also be considered. Further studies using WGS from either the Japanese population or other populations are needed. Seen as examples above, we highlight the necessity to integrate genetic information with in-depth clinical data to understand the full spectrum of gene functions when potentially disrupted by LoF. These findings also suggest that tolerability to LoF should be evaluated not only at the gene level but also at the transcript level.

### Sequences introgressed from Neanderthals and Denisovans

**Fig. 3.**
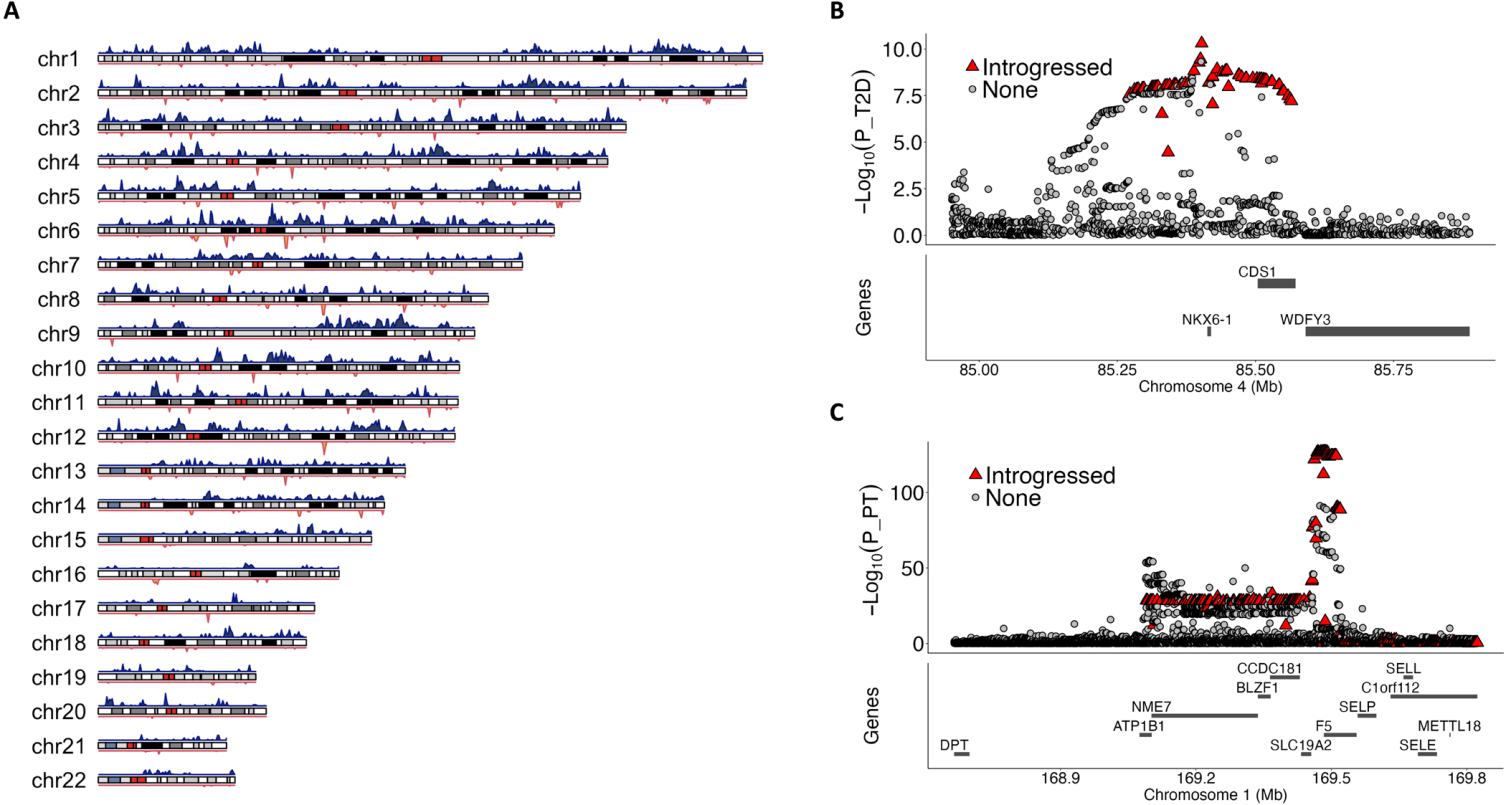
Introgressed sequences from archaic Neanderthals or Denisovans in the Japanese population. (**A**) Density plot illustrating the distribution of introgressed sequences across each chromosome. The upper track, shown in blue, represents sequences likely introgressed from Neanderthals, while the lower track displays sequences originating from Denisovans. (**B**) Variants likely introgressed from Denisovans in the *NKX6-1* locus are associated with T2D in the Japanese population. The triangle indicated the introgressed variants, and the gray dots indicated the nonintrogressed variants. (**C**) Introgressed variations from the Neanderthals in the *F5* gene are associated with PT.

EAs carry introgressed sequences from Denisovans and Neanderthals (*61*–*63*). However, the surveys of introgression have so far been restricted to a small number of samples in East Asia. To detect sequences likely introgressed from Neanderthals or Denisovans, we applied a recently developed probabilistic method, IBDmix, which does not use a modern reference population (see Materials and Methods). On an individual basis, the individual in JEWEL carries ~49 Mb of Neanderthal-derived sequences and 1.47 Mb of Denisovan-derived sequences (table S19). In total, we identified 3079 segments likely introgressed from Neanderthals and 210 segments likely introgressed from Denisovans, covering 772 and 31.46 Mb of the genome, respectively (Fig. 3A). Our results replicated 85% (2414 of 2843) of previously reported Neanderthal-introgressed segments based on the analysis of 104 Japanese in the 1000 Genomes project (1KGP) (fig. S10) (*63*). Notably, 47% (1439 of 3079) of Neanderthal-introgressed regions were not identified by the 1KGP Japanese in Tokyo, Japan (JPT) dataset, and 77% (1113 of 1439) of them were rare, with frequencies less than 5%. PCA of introgressed Neanderthal segments in JEWEL revealed no subregional differences (fig. S11). We compared Denisovan introgression in JEWEL to that in populations from the 1KGP dataset, as well as in Papuans and Philippine Ayta, both of which have a high proportion of Denisovan ancestry (*62*, *64*). The analysis revealed that the Denisovan-like segments in JEWEL significantly overlap with those in EA populations, while no statistical significance was found with those in Papuan and Philippine Ayta, indicating that Denisovan introgression in Japanese might be less relevant to that in Papuan and Philippine Ayta (table S20 and note S6).

Subsequently, we examined the phenotypic effects of the identified introgressed sequences on 106 traits based on GWAS summary statistics generated from BBJ (see Materials and Methods). We identified 44 archaic segments associated with 49 phenotypes (2 from Denisovans and 42 from Neanderthals). Among these, 43 associations have not been reported in comparison to a previous study (*65*). We validated 39 of 44 archaic segments by an alternative method SPrime and confirmed that 5 segments not detected by SPrime showed a high matching rate with the Neanderthal genome (see Materials and Methods) (*62*). The Denisovan-inherited segment at *POLR3E* was associated with height. The segment at *NKX6-1* was associated with type 2 diabetes (T2D) (Fig. 3B and Table 1). The *NKX6-1* segment has also been identified in other populations, including Papuans, Chinese [Han Chinese in Beijing (CHB) and Han Chinese South (CHS)], and Finnish (*62*). Moreover, archaic variants in this segment were found to be associated with T2D using GWAS data obtained from the FinnGen project (*P*_min_ = 8.65 × 10^−10^ at rs75560957) (*14*). For Neanderthal-derived segments, we observed 11 segments associated with seven diseases—T2D, coronary artery disease (CAD), stable angina pectoris (SAP), atopic dermatitis (AD), Graves’ disease (GD), prostate cancer (PrCa), and rheumatoid arthritis (RA) (Table 1). A pathway analysis identified “regulation of insulin secretion” as the top associated pathway (*P* = 1.9 × 10^−4^). At the *ADAMTS7* locus, the lead introgressed single-nucleotide polymorphism (SNP), rs11639375, was reported to be protective against CAD and SAP. While this SNP is observed in all major populations with high frequencies, upon further examination, it appears that rs11639375 in Japanese resides within a haplotype that is likely to have been introgressed from Neanderthals. The haplotype comprises 39 potentially archaic variants that exhibit a strong linkage disequilibrium (LD) with rs11639375 (*r*^2^ > 0.7). These variants are exclusive to EA and Latino Americans and are either absent or present at extremely low frequencies in other population groups (table S21). These data may suggest that this protective variant rs11639375 was once lost to EA and later restored through introgression. However, further analysis is needed to substantiate this hypothesis (note S7). We observed that a causal variant for AD, rs12637953, located in the *CCDC80* locus, is likely to have been inherited from Neanderthals. This variant was implicated as potentially functional via decreasing expression levels of an enhancer in CD1a^+^ Langerhans cells and skin epidermis cells by machine learning in silico prediction and was further experimentally validated (*66*, *67*). The introgressed segment at the *GLP1R* locus deserves attention. Variants at this locus were shown to be associated with T2D in a large-scale Japanese GWAS (*n* = 191,764), but not in European GWAS (*N* = 159,208), as previously reported (*68*). Through our analysis, we identified that the lead variants likely have archaic origins, specifically from Neanderthals. Further analyses using 1KGP data showed that this introgressed segment is present in Asians but absent in Europeans, which could account for the discrepancies in GWAS signals. In addition to archaic segments associated with diseases, we identified 37 distinct segments associated with 35 quantitative traits (table S22). As an example, archaic variants of the coagulation factor V (*F5*) gene showed positive associations with the bleeding trait (PT) (Fig. 3C). Notably, the same segment is associated with PT in the Icelandic population (*69*). We also confirmed that the Neanderthal-derived segment reported to be associated with severe COVID-19 (chr3: 45,859,651 to 45,909,024) was not detected in JEWEL (*70*). Last, the significant introgressed variants exhibited distinct population specificity in EAs compared to Europeans (fig. S12). The AFs were significantly higher in JEWEL compared to Europeans (*P* = 4.66 × 10^−8^, paired *t* test), and the median AF in the Japanese population is 21.5 times that of the AF in the European population.

**Table 1.** Introgressed segments associated with disease phenotypes in the Japanese population.

Introgressed segment	Lead archaic SNP	Reported *P*	Disease	Beta	Origin	Gene
chr4: 85200961–85426528	4:85301870:T:C	4.91 × 10^−11^	T2D	−0.134	Denisovan	*NKX6-1*
chr1: 39932346–40124123	1:39981740:G:A	3.16 × 10^−8^	T2D	0.062	Neanderthal	*BMP8A*
chr1: 160151058–160608637	1:160419940:A:G	3.29 × 10^−13^	GD	0.470	Neanderthal	*VANGL2*
chr2: 164906091–165538059	2:165381518:A:G	6.69 × 10^−10^	T2D	−0.172	Neanderthal	*GRB14*
chr2: 173140874–173598206	2:173321791:T:G	5.07 × 10^−12^	PrCa	−0.175	Neanderthal	*ITGA6*
chr3: 23163800–23502216	3:23210938:C:G	3.33 × 10^−15^	T2D	0.100	Neanderthal	*UBE2E2*
chr3: 111531421–113933832	3:112394029:T:C	2.88 × 10^−14^	AD	1.248	Neanderthal	*CCDC80*
chr6: 38249704–39053462	6:39037662:G:C	1.09 × 10^−17^	T2D	−0.092	Neanderthal	*GLP1R*
chr10: 63625277–64526183	10:64063077:T:C	1.26 × 10^−8^	RA	0.212	Neanderthal	*ZNF365*
chr12: 31070734–32216996	12:31441179:A:C	4.14 × 10^−25^	T2D	0.112	Neanderthal	*FAM60A*
chr15: 78635757–79216385	15:79019990:C:T	3.79 × 10^−10^	SAP	−0.078	Neanderthal	*ADAMTS7*
chr15: 78635757–79216385	15:79026723:G:A	2.90 × 10^−15^	CAD	−0.079	Neanderthal	*ADAMTS7*

### Evolutionary selection profile in the Japanese population

**Fig. 4.**
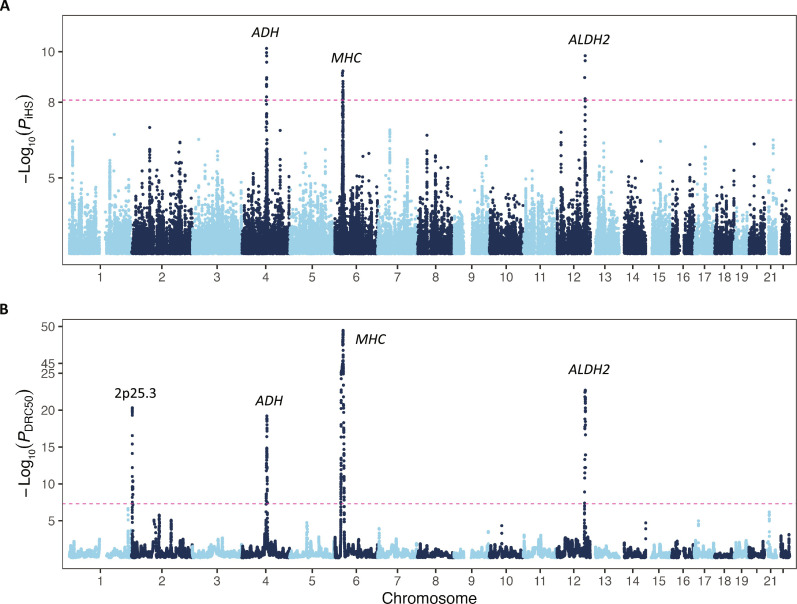
Positive selection signals in the Japanese population based on iHS and FastSMC analysis. (**A**) Manhattan plot showing the genome-wide distribution of *P*_iHS_ for variants in autosomes in the iHS analysis. The red horizontal dashed line indicates the genome-wide significance threshold of *P*_iHS_ = 8.24 × 10^−9^. (**B**) Manhattan plot of the FastSMC analysis; the genome-wide significance threshold is set at *P*_DRC50_ = 5 × 10^−8^.

We conducted genome-wide scans to detect candidate genomic loci that were likely subject to selection in the Japanese population with two methods: integrated haplotype score (iHS) analysis and FastSMC. The iHS method is effective at identifying selective sweeps based on phased haplotype information (*71*). FastSMC is an extension of the ASMC algorithm designed to rapidly identify pairwise identical-by-descent (IBD) regions at a specified coalescence time. By inferring IBD sharing, the analysis could identify regions that are overinherited from a limited number of common ancestors, potentially indicating recent positive selection (e.g., a quick frequency rise of a favorable haplotype) (*72*). By iHS, we identified three loci under positive selection at the genome-wide significance threshold (*P*_iHS_ = 8.24 × 10^−9^), including major histocompatibility complex (MHC), alcohol dehydrogenase (*ADH*) cluster, and *ALDH2* (Table 2 and Fig. 4A). The quantile-quantile plot indicated that there was no systematic bias (fig. S13). We further explored potential regional differences in the selection profile across five representative regions: West, East, Northeast, South, and Okinawa. We observed similar selection profiles across Hondo regions. However, note that the signals of *ADH* cluster and *ALDH2* were relatively weaker in Okinawa and did not reach genome-wide significance (fig. S14 and table S23). These differences could be due to a limited sample size of Okinawa or maybe varying selection pressures, necessitating further study. In addition, we used the FastSMC method as a complementary approach to validating the signals observed in iHS. We initially assessed the fit of the density recent coalescence (DRC) statistic. The density plot and the quantile-quantile plot for the empirical null model indicated that gamma fitting was generally well fitting, although it may not handle large DRC values well, leading to conservative approximate *P* values (fig. S15). In total, this method identified four candidate loci potentially targeted by selection in the past 50 generations, which include three loci significant in iHS (*ADH*, *ALDH2*, and MHC), and a candidate locus 2p25.3 (Table 3 and Fig. 4B). These three loci (*ADH*, *ALDH2*, and MHC) were also detected using the singleton density score (SDS) method in a previous study (*73*), further substantiating the presence of strong selection pressure on the autoimmune system and alcohol-metabolizing pathway for the Japanese population.

**Table 2.** Significant loci under positive selection detected by iHS analysis. BP, base pair position; DAF, derived AF; CHR, chromosome.

CHR	Position (Mb)	Cytoband	Lead SNP	DAF	Normalized iHS	*P*_iHS_	Candidate gene
4	97.20–99.30	4q22.3	rs79395698	0.914619	−6.51255	7.39 × 10^−11^	*ADH*
6	30.56–32.91	6p21.32	rs139510765	0.101505	6.19468	5.84 × 10−^10^	*MHC*
12	110.91–112.82	12q24.1	rs77768175	0.293151	6.41108	1.44 × 10^−10^	*ALDH2*

**Table 3.** Candidate loci detected to be under significant positive selection by FastSMC within the past 50 generations. DRC50, density of recent coalescence statistic within the past 50 generations.

CHR	Position (Mb)	Cytoband	*P*DRC50	Candidate gene(s)
2	2.79–5.27	2p25.3	4.91 × 10^−21^	*ADI1/COLEC11*
4	99.71–100.06	4q23	6.18 × 10^−20^	ADH cluster
6	27.05–32.74	6p21	3.30 × 10^−50^	*MHC*
12	113.15–113.43	12q24	1.97 × 10^−23^	*ALDH2*

## DISCUSSION

In this study, we generated JEWEL, a dataset consisting of clinical and WGS data from 3256 Japanese individuals across seven different regions in Japan. This comprehensive genetic dataset enables us to delve into uncharted territories concerning population and medical genetics of the Japanese population. We highlight several unique aspects of this study. Our analysis revealed fine population structure of the Japanese, echoing and lending supports to the “tripartite origins” model. We showcased potential clinical usages of JEWEL and examined the genetic legacy of Neanderthals and Denisovans in the Japanese and investigated their associations with various phenotypes, which constitutes the largest non-European analysis to date. Furthermore, the identification of genomic loci under recent selection enriched our understanding of adaptive evolution in the Japanese population.

The rich source of variants and comprehensive inclusion of samples across Japan in JEWEL, combined with PCA-UMAP and population genetics analyses, enabled us to construct a more refined Japanese population structure and propose triancestral origins of the Japanese population. Compared to the prior PCA-UMAP analysis that used array data from BBJ, our analysis, which is based on rare variants from WGS, offers enhanced resolution for distinguishing Japanese in Hondo (*35*). We reason that this is because rare variants typically emerged more recently than common ones and could be more informative in revealing the fine-scale genetic structure. In our current analysis, all Okinawa individuals were grouped into a single cluster in PCA-UMAP. This is likely due to the limited sample size, which may not capture the known genetic heterogeneity among subpopulations from different island groups within Okinawa (*74*). By incorporating samples from diverse regions of Japan, our study reveals genetic heterogeneity in Hondo Japanese, which align well with a recent study that examined array data from 11,069 individuals across all 47 Japanese prefectures (*34*). Moreover, our study provides additional insights into the potential ancestral components of the Japanese population, which we believe may be enhanced by the unbiased selection of SNPs from WGS (note S8).

Concerning ancestral origins of the Japanese population, we recommend that our data should be interpreted in the context of existing models, including the widely accepted “dual structure” model and the recently proposed tripartite origins model. The dual structure model, which suggested that the modern Japanese population formed by the admixture of indigenous hunter-gatherer Jomon people and rice-farming Yayoi migrants from continental Asia, has been extensively studied and is considered as the primary working hypothesis (*75*–*77*). A refined model, named “innerdual structure” proposed that genetic variations exist between “Central Axis” inland regions and “Periphery” coastal areas, influenced by multiple migration waves (*78*). A recent study of ancient genomes from Yayoi and Imperial Kofun periods introduced a further refined model, suggesting that the Japanese population may have three ancestral origins: Jomon, NEA, and EA (*41*). This is an intriguing hypothesis that specifically suggests the likely origins of continental ancestry. One limitation, however, is that the number of ancient genome samples, particularly those from Yayoi and Kofun periods, remains limited. As a result, some uncertainty persists, and the hypothesis has yet to be fully validated. The presence of Jomon and EA genetic component (e.g., Han Chinese) has been proposed to explain the dual-cluster pattern observed in PCA of the Japanese population. In line with this, the current study and previous research indicate that Okinawa has a higher genetic affinity to Jomon, while West, or regions near West, is genetically closer to the Chinese compared to other regions in Hondo (*33*, *34*, *40*). The qpAdm analysis offers further insights into potential ancestral origins of the Japanese population. We observed a reasonable fit of the tripartite model, involving Jomon, EA, and NEA, across our dataset, with the exception of Northeast. Crucially, two-way models using pairwise combinations of Jomon, EA, and NEA did not yield successful results. This outcome adds further supports for the triancestral model and indicates that the traditional “dual-structure” model may be insufficient. The observation that West had a closer genetic affinity to Chinese is potentially associated with a substantial influx of people with EA ancestry during the post-Yayoi period, with historical evidence indicating continued migration from the Korean Peninsula through the Kofun and Nara periods (250 to 794 CE) (*76*, *79*). This continued influx may have played a role in the formation of Japan’s first centralized imperial state during the Kofun period, which was established in West (in present-day Nara Prefecture) (*80*). This period also witnessed a substantial technological and cultural influx, characterized by Chinese influence. This is apparent in the comprehensive adoption of Chinese-style legitimation, language, and educational systems (*81*).

In our analysis, we observed K2, which is the highest in the present-day Japanese of Northeast, may serve as an additional genetic origin alongside Jomon and EA ancestries. We observed that this component has a significantly higher genetic affinity with Jomon and ancient Korean genomes in the TK era compared to West. The Northeast could be explained by a two-way admixture model using either Korea-TK_2 and Han instead of a triancestral model. It should be noted that Korea-TK_2 can be modeled as either 66% China_WLR_BA and 34% Jomon ancestry or by a triancestral model of 32% NEA, 43% EA, and 25% Jomon (*47*). These data may suggest a potential link between Northeast and NEA, although additional evidence is required to substantiate this connection. Historical records indicate that Northeast was inhabited by the so-called Emishi people, literally translated as “shrimp barbarians” (*82*). The origin of Emishi is somehow understudied and remains a matter of debate, but it was proposed that they might be related to NEA (*83*, *84*). In addition, it has been suggested that the Emishi people might have spoken a distinct Japonic language, akin to the historical Izumo dialect (*85*). Furthermore, despite the geographical distance between Northeast and South—specifically, Northern Kyushu, where evidence suggests that rice farming was first introduced in Japan (*86*)—it has been reported that local groups in the northern part of Northeast exclusively adopted rice during the early Yayoi period (*87*). This connection may be facilitated by human movements along the coastline of the Sea of Japan, potentially suggesting a link between the Northeast and the adoption of rice farming during the Yayoi period. Note that although the two-way fit model, using Korea-TK_2 and Han, demonstrates an acceptable fit, it implies the introduction of Jomon ancestry into the Northeast by continental immigrants, which seemed to be inconsistent with historical context (*76*). The unsuccessful fitting of the triancestral model could result from a higher proportion of Jomon ancestry in Northeast, possibly due to admixture with local populations with greater Jomon ancestry or owing to the limitations of our reliance on the precompiled Allen Ancient DNA Resource (AADR) dataset, which includes only 1240K SNP sites. The additional filtering on transversion sites further reduced the number of SNPs available for analysis. Ideally, this limitation would be addressed by processing directly raw sequencing alignment data; however, this extensive analysis is beyond the scope of the current study. Furthermore, the *f*_4_ analysis did not pinpoint a specific ancestral source among ancient NEA populations for Northeast. This important matter warrants future investigation, optimally involving new and more broadly and densely sampled ancient genomes from NEA. Last, we propose that genetic evidence be examined together with data from other domains, such as archaeology, culture, and linguistics. This interdisciplinary approach can enhance our understanding of the mysterious prehistory of the Japanese population. In addition, it should be acknowledged that both dual structure and tripartite origins models represent simplifications, although the latter may offer several advantages (note S9). The actual population history may be more complex and require further analysis.

In addition to the population structure analysis, we extensively analyzed coding variants in JEWEL. We observed that LoF variants in a set of genes were restricted to limited transcripts than expected by chance; sometimes, the genes are highly constrained, and carriers with those LoF variants displayed shared clinical phenotypes. A previous study has shown that more accurate transcript-level annotation could be achieved by incorporating isoform expression data (*88*). Our results suggest that WGS data offer a potential opportunity to develop a new metric or score of the constraint spectrum by comparing the intolerance of LoF across transcripts within a given gene. We have demonstrated that the extensive clinical information available in JEWEL can be effectively used to uncover potential link between genotype and phenotype.

We reported archaic-introgressed variants are associated with a broad range of phenotypes, including immune and metabolic phenotypes in present-day Japanese. It has been shown that introgressed Denisovan sequences at the *EPAS1* locus have helped Tibetans adapt to high-altitude environments (*89*). However, beyond a few specific examples such as *EPAS1*, the impacts of Denisovan introgression on human phenotypes remain less understood, particularly in comparison with introgression from Neanderthal (*90*). In this context, we have shown that Denisovan-derived segments at *NKX6-1* and *POLR3E* are associated with T2D and height, respectively. A previous study had reported the likely Neanderthal-introgressed segments associated with disease phenotypes using publicly available BBJ GWAS sumstats and precalled archaic variants (*65*). Our study replicated all reported findings and reported 43 additional associations, which greatly expanded the number of introgressed linked with phenotypes and enhanced our understanding of the phenotypic impact of archaic sequence in the Japanese population. In particular, the association between Neanderthal-derived variants of *GLP1R* and T2D is intriguing, considering population specificity and the development of oral semaglutide, a glucagon-like peptide-1 (GLP-1) analog, for treating T2D (*91*). Future research could investigate whether individuals with these archaic variants respond differently to semaglutide treatment and explore the presence of additional archaic segments that could be potential targets for drug discovery. In addition to this specific example, we have demonstrated that overall significant introgressed variants exhibit population specificity in EAs compared to Europeans, which suggests that these archaic variant–phenotype associations might be missed when only examining European data.

Our selection analysis complements genome-wide scans for recent selection signatures in the Japanese population, using methods including SDS and ASMC. In a study based on 170,882 individuals from the BBJ, 29 candidate loci were suggested to be under selection in the past 150 generations based on DRC_150_ statistics using ASMC. In addition, two loci, including the ADH cluster and MHC, were identified by the iHS method (*92*). However, the selection profile within a more recent time frame using DRC-based statistics has yet to be explored. Our analysis indicated that *MHC*, *ADH*, and *ALDH2* are under recent positive selection according to iHS, FastSMC, and previously reported SDS analysis. There are potential differences in ADH/*ALDH2* signals between Okinawa and Hondo groups, and this may warrant further analysis (note S10). We also observed a candidate locus at 2p25.3. While several genes in this locus warrant consideration as candidate genes, we recommend conducting further replication analyses before focusing on any specific gene.

In summary, our study has unveiled genetic characteristics of the Japanese population that were not previously discernible with microarray data. The extensive dataset created in this study also serves as a reference for future genetic research within and beyond the Japanese population. The study emphasized potential applications of WGS in personalized medicine and other clinical settings and highlighted the importance of extending WGS to diverse populations to decode genetic characteristics and better understand human history in a population-specific manner.

## MATERIALS AND METHODS

### WGS and variant calling

Briefly, sequencing was done at two different depths: (i) 1502 individuals were sequenced at a ~30× (mean, 32.3; median, 31.8) with Illumina HiSeq 2500 (rapid mode or V4) or Illumina HiSeq X Five platform; (ii) 1786 individuals were sequenced at a ~20× depth (mean, 19.9; median, 19.5) using the Illumina HiSeq X Five platform. Sequencing libraries were prepared using standard Illumina protocols and paired-end sequencing was conducted (2 × 125, 2 × 150, or 2 × 160 bp). After sequencing, we performed sample quality control (QC) to remove low-quality sequenced and closely related individuals. In total, 32 of 3288 individuals were excluded, leaving 3256 samples (note S2). After alignment of reads to a human reference (hg19) using BWA-MEM (v0.7.5 or v0.7.13) and removal of duplicated reads, we conducted the joint genotyping calling following the best practice proposed by GATK (v3.2-2). We performed further SNP QC with the following exclusion criteria: (i) read depth (DP) < 5; (ii) genotype quality (GQ) < 20; (iii) DP > 60 or GQ > 95; (iv) variants failed the variant quality score recalibration filtering. Detailed procedures of WGS have also been described previously (*73*). Among 3256 individuals, array-based genotyping data of 3157 individuals were available. These individuals were genotyped using Illumina Human OmniExpress Exome BeadChip or a combination of Illumina HumanOmniExpress and HumanExome BeadChips. We compared the genotyping concordance rate for QC-passed SNPs, which has a call rate ≥ 99% and a Hardy-Weinberg equilibrium *P* value (*P*_HWE_) ≥ 1 × 10^−6^. Because sequencing depths are different for two subcohorts at 20× and 30×, we examined metrics related to genotyping quality, including Ti/Tv, concordance, heterozygosity rate, and number of singleton coding variants per individual. We observed comparable values among the two cohorts (table S3). After the removal of singletons, phasing was conducted by Eagle (v2.4.1) for all biallelic variants on each chromosome using the default parameters (*93*).

### Population structure and population genetic analysis

PCA was conducted by PLINK (v1.9) based on pruned common or rare variants. We defined common variants as those with a minor AF (MAF) ≥ 0.01 and rare variants as those with an MAF between 0.001 and 0.01. We performed pruning for both categories of variants to select tag SNPs by PLINK with the following parameter: --indep 500, 50, 0.2. Variants in MHC region (chr6: 25 to 34 Mb, hg19) were excluded from the analysis. A total of 184,036 common and 1,835,116 rare variants were obtained after pruning and were used for PCA. The UMAP analysis of the top 20 PCs from rare variant–based PCA was conducted with the UMAP package (v1.1) in R (version 3.1). ADMIXTURE (v1.3.0) was used for the admixture analysis based on the 184,036 pruned common variants (*94*). To determine the optimal *K* value, we used the Structure Selector software (*38*). To avoid unbalanced sample selection, we randomly selected 50 samples from each region (excluding Okinawa, for which we included all 28 samples) and conducted admixture analysis from *K* = 2 to 6 with three repetitions at each run. In addition, we used the badMIXTURE to visualize the model fitting according to the recommended analysis procedures (*39*).

Using ADMIXTOOLS (v7.0.2) and admixr package, we computed *f*_4_ and *f*_3_ statisitcs (*95*). We calculated *f*_4_ ratio with the form *f*_4_ (a: Chinese Dai in Xishuangbanna; b: CHB; x: target population; c: Jomon; o: Yoruba in Ibadan, Nigeria). In this formula, “a” represents a population related to “b” but not involved in the admixture. On the other hand, b and “c” are the source populations contributing to the admixture, and x is the target admixed population. Last, “o” serves as the outgroup population. The a and 1-a reflect the proportion of admixture from CHB and Jomon. To ensure equal sample sizes, we selected ~30 individuals from each region considering PCA-UMAP information and merged with the AADR dataset (V54.1.p1), matching the “1240K” variants in the AADR panel. Only transversion sites were used for the analysis. We used Jomon individuals labeled “Japan_HG_Jomon” from a previous study for the *f*_4_ ratio test (*41*). Furthermore, we computed outgroup *f*_3_ statistics with the form *f*_3_ (a: target population; b: Chinese Han; o: Papuan). The statistics reflects shared genetic drift between two source population a and b, and large values indicates greater shared genetic drift and thus. We set subregion groups in JEWEL as a, Han as b, and Yoruba as o. We also calculated *f*_4_ statistics using the formula *f*_4_ (Mbuti, ancient genome; Northeast, West) and focused on results supported by more than 50,000 SNPs. We included ancient genomes from China, Korea, and Japan from previous studies (*41*, *42*, *45*, *47*, *96*). We defined NEA by grouping China_WLR_BA_o and China_HMMH_MN, as used in the previous study (*41*). In addition, following the previous report, we defined Korea-TK_2 group as AKG_10203 and AKG_10207. This group has been shown to be more closely related to present-day Japanese and other ancient Japanese groups with high Jomon ancestry (*47*). We conducted a qpAdm analysis (qpAdm version 1520) to model the three-way or two-way admixture following the configuration outlined in a previous study (*41*). We used a set of nine Eurasian populations as right group, comprising Sardinian (*n* = 3), Kusunda (*n* = 2), Papuan (*n* = 14), Dai (*n* = 4), Ami (*n* = 2), Naxi (*n* = 3), Tianyuan (*n* = 1), Chokhopani (*n* = 1), and Mal’ta (*n* = 1), with the option set to “allsnps: YES.”

### Identification of LoF variants

We performed variant annotation for all biallelic variants using the software VEP (v87) and the LoF transcript effect estimator package (*36*). For missense variants, we incorporated annotations or in silico predictions from 30 different tools, and the risk score was the sum of the number of tools supporting the variant to be deleterious (*97*). We defined LoF variants as those that cause premature stop codons (stop-gained), small-sized indels that shift the coding sequence (frameshift), or variants changing two immediately adjunct nucleotides to the splicing sites (splicing variants). Using LoF transcript effect estimator, we filter high-confidence LoF variants by filtering out those likely annotation artifacts (e.g., the LoF variants in the 3′ end of the transcripts). For LoF variants with a minor allele count ≥ 3, we examined whether the occurrence of the LoF variant is associated with UMAP1/2 by logistic regression analysis using R (v3.1).

### Human knockouts

We screened individuals carrying any rare homozygous LoF variant(s) (MAF < 0.01) or rare compound heterozygous LoF variants. We restricted this analysis to LoF variants in genes that contain pathogenic variants in the ClinVar database (v20201208) (www.ncbi.nlm.nih.gov/clinvar/). To identify potential compound heterozygotes, we filtered for instances where multiple LoF variants were present within the same gene of the same individual, and we examined the phased haplotypes. For all candidate human knockouts, we performed manual curation and visually examined the raw alignment reads by the Integrative Genomics Viewer. To identify genes in which LoF variants occurred in fewer transcripts than expected by chance, we selected 4192 genes that had more than one LoF variant and performed a simplified permutation-based test. For a gene with *N* LoF variants, we summed the actual number of transcripts affected by these *N* LoFs, denoted as *J*. Next, we randomly selected *N* positions within the gene’s coding region based on GENCODE gene annotation (v19) (www.gencodegenes.org/human/), and we counted the total number of transcripts overlapped with *N* positions, denoted as *K*. We repeated this procedure 1000 times to obtain a list of values from *K*_1_ to *K*_1000_. The empirical permutation *P* value was calculated as the rank of *J* among the 1000 *K* values, sorted in ascending order.

### Detection of introgressed sequences and variants

To identify sequences that are likely introgressed from Neanderthals or Denisovans, we applied a recently developed computational method IBDMix (*63*). In contrast to other methods, IBDMix used an archaic reference genome to infer the introgression segments. We conducted following filtering steps for the introgression analysis. Briefly, for Neanderthal and Denisovan genomes, minimal filter masks were applied (obtained from https://bioinf.eva.mpg.de/). For human genome sequences, we applied the 1KGP accessibility mask (downloaded from: http://ftp.1000genomes.ebi.ac.uk/vol1/ftp/release/20130502/supporting/accessible_genome_masks/20140520.strict_mask.autosomes.bed). We masked out sequences within 5 bp of the indels and only autosomes were analyzed. The called introgressed sequences with a logarithm of the odds ratio for linkage score ≥ 4 and a length ≥ 50 kb were retained for downstream analyses. To identify introgressed variants and exclude those misclassified due to incomplete lineage sorting, we focused on high-confidence introgressed segments. Briefly, we obtained phased haplotypes for each introgressed segment and calculated match rates to the Neanderthal and Denisovan genomes, excluding variant sites with unknown archaic status. The high-confidence Denisovan segments were defined with a Denisovan match rate ≥ 0.5 and a Neanderthal match rate < 0.5. The high-confidence Neanderthal segments were defined as a Neanderthal match rate ≥ 0.7. The variants observed in over half of introgressed haplotypes were selected as likely introgressed variants. We screened introgressed genetic variants to determine their association with both disease and quantitative traits. We used summary statistics from previous studies encompassing 42 diseases and 64 quantitative traits, all based on the BBJ dataset (*98*). We filtered associations that surpassed the genome-wide significance level at 5 × 10^−8^. To exclude association due to LD with nonarchaic variants, for all loci in which the archaic variant was not the lead variant, we calculated the *r*^2^ between lead archaic variant and lead GWAS variant and removed those pairs with *r*^2^ < 0.9. We conducted a comparison of introgression segments with those previously reported within the Japanese population based on 1KGP JPT data (*63*). In addition, we used an alternative method, SPrime, to validate introgressed segments that exhibited significant phenotypic associations. For this analysis, we set Yoruba in Ibadan, Nigeria as the outgroup and followed default parameters. For segments likely introgressed from Denisovans, we conducted an enrichment analysis to ascertain whether these segments significantly overlap with those reported in a previous study (*62*). Populations with fewer than 30 segments detected were excluded, as this could result from indirect inheritance. The 1KGP data for this analysis were obtained from the following link: https://data.mendeley.com/datasets/y7hyt83vxr/1. The Philippine Ayta data were obtain from a previous study (*68*). We used Bedtools “fisher” utility to perform the enrichment analysis (https://bedtools.readthedocs.io). We conducted a pathway analysis to identify the biological pathways that exhibited enrichment in genes containing archaic variants associated with diseases. This analysis was carried out using Enrichr. (https://maayanlab.cloud/Enrichr/). To confirm the association between archaic variants in *NKX6-1* locus and T2D, we obtained the GWAS summary statistics from the FinnGen database (https://r9.finngen.fi/) (*14*).

### Analysis related to natural selection

The selscan software (v1.3.0) was used to calculate the iHS scores using the default parameters (*99*). We restricted the analysis of autosomal biallelic variants with an MAF ≥ 0.01. Next, on the basis of the human-chimp-macaque alignment provided by Ensembl (downloaded from http://ftp.1000genomes.ebi.ac.uk/vol1/ftp/phase1/analysis_results/supporting/ancestral_alignments/), we retained variants of whose ancestral allele was present in chimpanzee or macaque. We used a Japanese-specific recombination map created using the 1KGP JPT dataset to calculate iHS (*100*). We normalized unstandardized iHS scores across 100 AF bins. The approximate *P*_iHS_ values were calculated by fitting normalized iHS scores, assuming a normal distribution. The genome-wide significance threshold was determined at 8.24 × 10^−9^ based on Bonferroni correction (0.05/6,066,864 variants). To exclude potentially false positive signals, we removed loci showing extremely high or low recombination rates, loci containing only a single significant variant, and loci with segmental duplication in the nearby region. We conducted a subanalysis on samples from five representative regions: West, East, Northeast, South, and Okinawa, using the same procedure. For Okinawa, we limited the analysis to variants with an AF of 5% or higher, owing to the limited sample size. In addition to iHS, we used FastSMC to identify genomic loci likely targeted by selection in the Japanese population. Since the method was developed and tuned using microarray data, we extracted a superset of variants included in the Illumina HumanOmniExpressExome BeadChip array, the Illumina Infinium Asian Screening Array, and the Affymetrix Japonica array. By analyzing the locus-specific IBD sharing patterns, the DRC within the past 50 generations (DRC_50_) was calculated by FastSMC. We summarized the mean DRC_50_ for each sliding window at a size of 0.05 centimorgan. The decoding file was prepared from the 1KGP JPT demographic and AF file. We then fitted a Gamma distribution to the averaged DRC_50_ values using the neutral regions in the genome. We excluded genetic loci reported to be under positive selection in the Japanese population based on genome-wide analysis (*73*, *92*). We further iteratively removed regions that showed evidence of being targeted by selection based on the DRC statistic. On the basis of this null model, we derived approximate one-sided *P* values. The genome-wide significance threshold was set at 5 × 10^−8^ for *P*_DRC50_. To assess the overlap between loci identified as genome-wide significant by iHS or DRC statistics and known SVs, we analyzed the phase 2 dataset from the Human Genome Structural Variation Consortium (http://ftp.1000genomes.ebi.ac.uk/vol1/ftp/data_collections/HGSVC2/release/v1.0/PanGenie_results/pangenie_merged_multi_nosnvs.vcf.gz).

## Supplementary Materials

### This PDF file includes:

Notes S1 to S10 Figs. S1 to S18 Legends for tables S1 to S24 References
