# Supplementary material for: Decoding triancestral origins, archaic introgression, and natural selection in the Japanese population by whole-genome sequencing
Source: Sci Adv. 2024 Apr 17;10(16):eadi8419. doi: 10.1126/sciadv.adi8419 (PMC11023554; doi:10.1126/sciadv.adi8419)
Supplement: Supplementary file 1 — Notes S1 to S10 Figs. S1 to S18 Legends for tables S1 to S24 References [file sciadv.adi8419_sm.pdf]

Science Advances
AAAS

Supplementary Materials for

Decoding triancestral origins, archaic introgression, and natural selection in the Japanese population by whole-genome sequencing

Xiaoxi Liu *et al.*

Corresponding author: Chikashi Terao, chikashi.terao@riken.jp

Sci. Adv. **10**, eadi8419 (2024)
DOI: 10.1126/sciadv.adi8419

The PDF file includes:

Notes S1 to S10
Figs. S1 to S18
Legends for tables S1 to S24
References

Other Supplementary Material for this manuscript includes the following:

Tables S1 to S24

Supplementary Text

Note S1: JEWEL, the pilot stage and beyond

JEWEL was established with the aim of sequencing samples from the BBJ. In the pilot stage of the JEWEL project, we investigated pathogenic variants associated with hereditary cancer in 1,037 JEWEL samples (*101*). A genome-wide scan employing the Singleton Density Score (SDS) method was conducted on 2,234 WGS to identify recent positive selection signatures in the Japanese population. This analysis revealed four candidate loci: ADH cluster, major histocompatibility complex (MHC) region, *BRAP-ALDH2*, and *SERHL2* (*73*). We now present the expanded JEWEL dataset, comprising a total of 3,256 high-depth WGS. Using this dataset, we generated a reference panel and utilized it for imputation in large-scale GWASs (*102*, *103*). In a separate study, SVs in JEWEL were extensively examined, leading to the identification of SVs significantly associated with various diseases and traits through imputation and association analysis (*104*).

Note S2: WGS Sample QC

A total of 3,288 samples underwent WGS, and 3,256 passed the QC criteria. The samples that failed QC were excluded due to several filters. First, we excluded five samples that were marked as "gDNA failure" in the sequencing summary. Next, we removed two samples that failed the read quality control, meeting the following criteria: I) high sequencing index error, II) high fraction of uniformity and coverage, III) high GC bias, IV) high fraction of duplication reads, and V) low sequencing depth. We then excluded 19 samples that showed identity-by-descent (IBD) or extreme heterozygosity. Subsequently, two samples with low concordance compared to microarray data were removed. Finally, three subjects with high contamination rates (determined as CHIPMIX >= 0.26 and FreeMiX >= 0.25) were excluded.

Note S3: PCA-UMAP analysis

It should be noted that the population structure pattern revealed by PCA-UMAP shown in Fig. 1C was based on rare variants with an allele frequency ranging between 0.01% to 1%. PCA-UMAP using common PCA (MAF >= 1%) could not fully capture the fine-scale population structure. We reasoned that the use of rare variants provided higher resolution in depicting population structure compared to common variants. However, we are not entirely clear on the

theoretical rationale behind the population structure revealed by PCA-UMAP using rare-variant and its correlation with the ADMIXTURE results (*105*). Furthermore, UMAP is a stochastic algorithm that utilizes randomness to expedite approximation steps. Consequently, different runs of UMAP using different initial seeds can yield slightly different results, but we have observed that UMAP tends to exhibit stability in the outcomes. Our results are generally consistent with the previous PCA-UMAP analysis applied to BioBank Japan (BBJ) array genotyping data (*35*). In the previous study, a structure exhibiting a northeast-to-southwest pattern was observed, and non-mainland clusters appeared to correspond to sub-populations from fine structure analysis. Regarding the sub-clusters of West and South, we suspected they represent individuals from regions such as particularly isolated islands, but due to the lack of detailed geographic information on the samples, this could not be confirmed. Future studies with comprehensive geographic information and a larger sample size are warranted to address these issues.

Note S4: Further note on ADMIXTURE analysis

A recent study identified a total of 27 unusual genomic regions that could potentially distort the population structure inference (*106*). Excluding these regions may enable a more comprehensible population structure through PCA and ADMIXTURE analysis. In order to examine any potential influence from these problematic regions on our ADMIXTURE results, we re-analyzed both PCA and ADMIXTURE by excluding variants within these regions. We first lift-down 11 out of these 13 troublesome regions to hg19. We noted that only a few SNPs used in PCA/ADMIXTURE analyses were located in these problematic regions (147 out of 184,036 SNPs, or 0.08%). We did not observe any noticeable differences in either PCA or ADMIXTURE analysis after removing 147 SNPs. We note there is a small change in the mean ADMIXTURE K values by region groups (Table S24).

Furthermore, we need to be careful to not interpret present-day Okinawa people as a least-admixed ancestry component or least-admixed descendants of Jomon. First, the limited sample size from Okinawa may not fully capture the extent of genetic heterogeneity among Okinawa sub-groups, as demonstrated by several analyses (*35*, *107*, *108*). Second, the genetic composition of Okinawa contained continental influences and is also likely to have been significantly shaped by factors like geographical isolation and strong genetic drift (*109*). This might partially explain

subtle differences observed between ADMIXTURE and the f_4 ratio analysis. Specifically, in ADMIXTURE analysis, South showed the second highest proportion of K1 (which might be due to genetic exchange facilitated by regional proximity) whereas f_4 ratio analysis indicated that Northeast had the second highest proportion of Jomon ancestry. The latter result aligns with a study focusing on Jomon allele analysis (*110*).

Finally, we attempted to run ADMIXTURE with both common and rare variants. However, due to the computational limitations of the original ADMIXTURE software, we were unable to process approximately 2 million variants within a reasonable timeframe. To address this challenge, we opted for a more computationally efficient version of ADMIXTURE called SCOPE (*111*). This analysis yielded a pattern consistent with that obtained using only common variants but appeared to offer more detailed insights. Specifically, it indicated that individuals from Okinawa also carry a small amount of the K2/K3 component, as illustrated in Fig. S16. This observation may align with a recent study that identified a Northeast Asian (NEA) component in historical genomes from the southern Ryukyu Islands (*48*). Additional analyses are required to investigate this potential connection.

Note S5: Pathogenic or likely pathogenic variants

We screened known pathogenic or likely pathogenic variants (P/LP) reported in the ClinVar database (version 20201208) in the JEWEL dataset. We restricted the analysis to reported variants that have a review status golden star >= 2 (practice guideline, reviewed by expert panel and multiple submitters, no conflicts) and without conflicting interpretations. In total, we identified 317 known P/LP variants. An important nuance is that subjects enrolled in JEWEL were patients of cancers (breast, prostate, colorectal, gastric) with an early onset, and patients of myocardial infarction, drug eruption, dementia, and therefore compared with non-disease population cohorts, it is more likely to identify P/LP variants. Each individual carries 1.36 +/- 0.56 (mean +/- SD) pathogenic variants. We ranked the genes according to the number of known P/LP variants and observed that *BRCA2* (N=12), *LDLR* (N=10), and *BRCA1* (N=6) were the top genes. A total of 1,723 LoF variants were observed in ClinVar genes harboring P/LP variants (1,433 genes) but not previously reported in ClinVar. We noticed genes, including *TTN, PCDH15*, and *SPG11,* have the highest number of ClinVar unreported LoF. *TTN* is a gene

associated with Myopathy, and this gene was shown to have the highest putatively pathogenic variants among mendelian cardiovascular disease genes in the UK Biobank (*97*).

Note S6: Overlap of Denisovan segments in JEWEL with those in other populations

We examined whether Denisovan segments identified in JEWEL (N = 220) significantly overlap with those reported in a previous study that included populations from the 1000 Genomes Project (1KG) and Papua New Guinea (*62*). The most notable enrichment was observed in East Asian populations, specifically CHS, JPT, and CHB, whereas no significant enrichment was found in Papua New Guinea (*P* = 0.54, enrichment ratio = 1.229). Since our analysis focused on segments with a high matching rate to the Denisovan genome, as identified by IBDMix, and the previous data were obtained using the SPrime algorithm—which may be more sensitive than IBDMix—we conducted additional analyses to ensure that the results were not influenced by the choice of calling algorithm. We analyzed Denisovan segments determined using the SPrime and found a higher fold of enrichment in Papuans than that based on IBDmix (enrichment ratio = 1.774), although the *P* value slightly fell short of statistical significance (*P* = 0.088). These results appear to suggest a weak signal of enrichment. We next repeated the analysis solely using data generated by the previous study focusing on Denisovan segments in 1KG JPT samples and in Papuans *(62)*. We again observed marginal significance (*P* = 0.054, enrichment ratio = 2.353). Based on these findings, it seems that Denisovan sequences in the Japanese population are largely similar to those in East Asian populations but show a weak enrichment when compared to those in Papuans.

The above observations seem to be plausible considering the presence of a shared Denisovan introgression between East Asians and Papuans, as well as introgression specific to EAS. It has been proposed two distinct pulses of introgression occurred in EAS (*62*). One pulse appears to be common to both EAS and Papuans, while the other pulse is specific to EAS (*112*). The precise timing of Denisovan introgression events in EAS remains largely unclear, while it has suggested that the EAS-specific Denisovan introgression, dated at approximately 48 ka, predates the Papuan-specific pulse which was estimated at 30 ka ago, and temporally closer to the initial Denisovan introgression shared between Asian and Oceanian lineages, estimated at around 45 ka (*112*). Furthermore, it was suggested that the EAS-specific introgression had a higher affinity to

the Altai Denisovan genome, whereas the EAS-Papuans shared introgression exhibited moderate affinity to the Altai Denisovan genome (*62*). In this context, when analyzing data via IBDMix, we tend to identify introgressed segments specific to the EAS lineage due to their high affinity with the Altai Denisovan genome. Conversely, using Sprime, we might be able to call more introgressed segments common to both EAS and Papuan lineages.

Note S7: Introgressed haplotype reintroduced lost allele at *ADAMTS7* locus

We observed that SNP rs11639375 (chr15:79024214:G:A) in the *ADAMTS7* locus had previously been identified as a lead variant in coronary artery disease (CAD) GWAS, where it exhibited a protective effect (*30*). Notably, this SNP is in high linkage disequilibrium (LD) (r2 = 0.91) with the top Neanderthal-derived variant rs74508956 (chr15:79022322:T:GExamining allele frequencies of the two SNPs using the Geography of Genetic Variants Browser (https://popgen.uchicago.edu/ggv) revealed that rs74508956 is restricted to populations from East/South Asia and Latin America, while rs11639375 is broadly distributed in all populations, including Africans (Fig. S17). To provide additional evidence that rs11639375 is located within an introgressed haplotype rather than merely in LD with a single archaic SNP, we found that 39 SNPs are in high LD with rs11639375 (r2 >= 0.7). These variants exhibit a frequency distribution pattern similar to that of rs74508956 as shown in Table S20.

We confirmed the same allele frequency distribution pattern using data from gnomAD. Based on the data above, we speculate that rs11639375 might have been lost during a bottleneck when East Asian populations separated from European populations approximately 35,000 to 50,000 years ago. Subsequently, this allele might have been reintroduced through the Neanderthal introgressed segment.

Note S8: ADMIXTURE analysis using array data

We observed an optimal K value of 3, in contrast to K = 2 reported in a previous study (*34*). This discrepancy may stem from different variants were used. We speculated that common variants from pre-designed microarrays may not fully capture the LD structures in the Japanese population, as compared to variants unbiasedly selected from WGS. This could potentially introduce a higher noise level into the ADMIXTURE results. In our study, array-based

genotyping data were available for 3,157 out of 3,256 subjects. To test this hypothesis, we ran ADMIXTURE using the array data, applying the same protocol as used for the WGS data, and plotted the results for the same individuals. Empirically, we found that the WGS-based ADMIXTURE analysis produced cleaner data, while the array-based approach exhibited more noise (Fig. S18). However, a similar pattern concerning the genetic cline from Northwest to West was observed in both datasets. Further analyses will be essential in the future to validate and extend these findings. This may include comprehensive sampling with detailed sample information to enhance the robustness and generalizability of the results.

Note S9: Perspective on "dual structure" and "tripartite origins" models

We would like to discuss differences and implications between the "dual structure" model and "tripartite origins" model, arguing that the latter might be a superior theoretical framework. First, although there is a consensus from both models that there had been multiple migration waves in Japan's prehistory, the "dual structure" model suggests/implies that the genetic differences in the Japanese population primarily stem from the extent of admixture between those continental people and Jomon. In comparison, the "tripartite origins" model emphasizes the significance of admixture between EA and NEA ancestry. Second, the term "Yayoi" in the "dual structure" model is ambiguous, as it can refer to heterogeneous groups of people. While defining Yayoi individuals as those with continental ancestry who migrated to Japan during the post-Jomon period may be historically or genetically valid, this definition lacks both specificity and resolution. Various waves of migration to Japan, from Yayoi period to post-Kofun era, could encompass individuals with EA, NEA, or a mixture of known or unknown continental ancestries. Although all these groups could technically be considered as having originated from Continental Asia, such a broad categorization fails to capture the nuanced differences among them. On the other hand, in the "tripartite origins" model, if we assume that the early-arriving people in Yayoi era and the later-arriving people in Kofun era may have different genetic or cultural/linguistic backgrounds—albeit the genetic differences may be challenging to distinguish due to the shallow divergence time between NEA and EA and scarcity of ancient genomes at the moment—this model may offer a better fit to the major cultural and societal shifts observed during the Yayoi and Kofun periods. Thirdly, the 'tripartite origins' offer a plausible explanation for the origin of the Japanese language by introducing NEA ancestry into the framework, as evidence suggests

that the Proto-Japonic language can be traced back to Northeast Asian roots (*42*). While it should be acknowledged that both "dual structure" and "tripartite origins" models are themselves simplifications and that the actual population history might be more complex. Additionally, we suggest that future studies could investigate the potential link between Northeast Asia and the Izumo civilization. It has been shown that the phonology and tone system of dialect in present-day Northeast share similarities with those of Izumo, suggesting a potential migratory connection between the people of Northeast and Izumo (*85*). This potential link between Northeast/Emishi and Izumo is intriguing given the prominent position that the ancient Izumo occupies in Japanese prehistoric mythology and religion (*113*, *114*).

Note S10: Difference of ADH/*ALDH2* signal between Okinawa and Hondo and potential sources of natural selection

We observed differences in the selection signals of ADH cluster and *ALDH2* between Hondo and Okinawa, which are interesting and may warrant further analysis. Reasons for the positive selection of these alcohol metabolism genes remain unknown, but it was proposed might be related to the large-scale adoption of rice cultivation in East Asia. One hypothesis suggested that elevated levels of acetaldehyde may confer a protective effect against parasitic infections, particularly from anaerobic and microaerophilic gut organisms like *Entamoeba histolytica* (*E. histolytica*) (*115*, *116*). This amoeba relies on a key bifunctional alcohol/aldehyde dehydrogenase protein, EhADH2, for its growth and survival, where this protein plays a vital role in the utilization of glucose as an energy source via the fermentative pathway (*117*, *118*). Elevated acetaldehyde levels in the blood due to *ALDH2* deficiency could potentially impair the function of EhADH2, offering a form of protection against infection. This implied notion could find indirect support from the fact that nitroimidazole, an ALDH inhibitor, has been used as the first-line medication to treat *E. histolytica* infection (*119*). Should this hypothesis be validated, individuals carrying functional variants of *ALDH2* could benefit from potential resistance to parasites. If such an advantage exists, it may be especially relevant in East Asia, where farming has a long history of widespread practice. In this context, the likelihood of encountering parasites like *E. histolytica* may be increased due to activities such as working in rice paddy, where anaerobic conditions in the soil can create an environment conducive to certain parasites. Additionally, the use of human or livestock excreta as fertilizer in agriculture can further

increase the risk of exposure to pathogens (*120*). If the link between selection of *ALDH2* and the advent of rice farming is indeed true, differing selection pressures could be present in Hondo and Okinawa, considering that rice farming was established in these regions at different times: approximately 3,000 years ago in Hondo and around 1,000 years ago in Okinawa during the Gusuku period (*121*). While other hypotheses exist, such as protection against hepatitis B virus (*122*). Moreover, a study has shown that ADH genes and *ALDH2* are associated with the dietary preferences of Japanese individuals, which include the consumption of green tea, milk, yogurt, natto, tofu, and fish, in addition to their roles in alcohol consumption (*123*). Survival analysis conducted on the BBJ dataset revealed that carrying functional variants of *ADH1B* and *ALDH2* had a positive impact on all-cause mortality in the Japanese population. Notably, these effects were independent of alcohol consumption (*124*).

Supplementary Figures

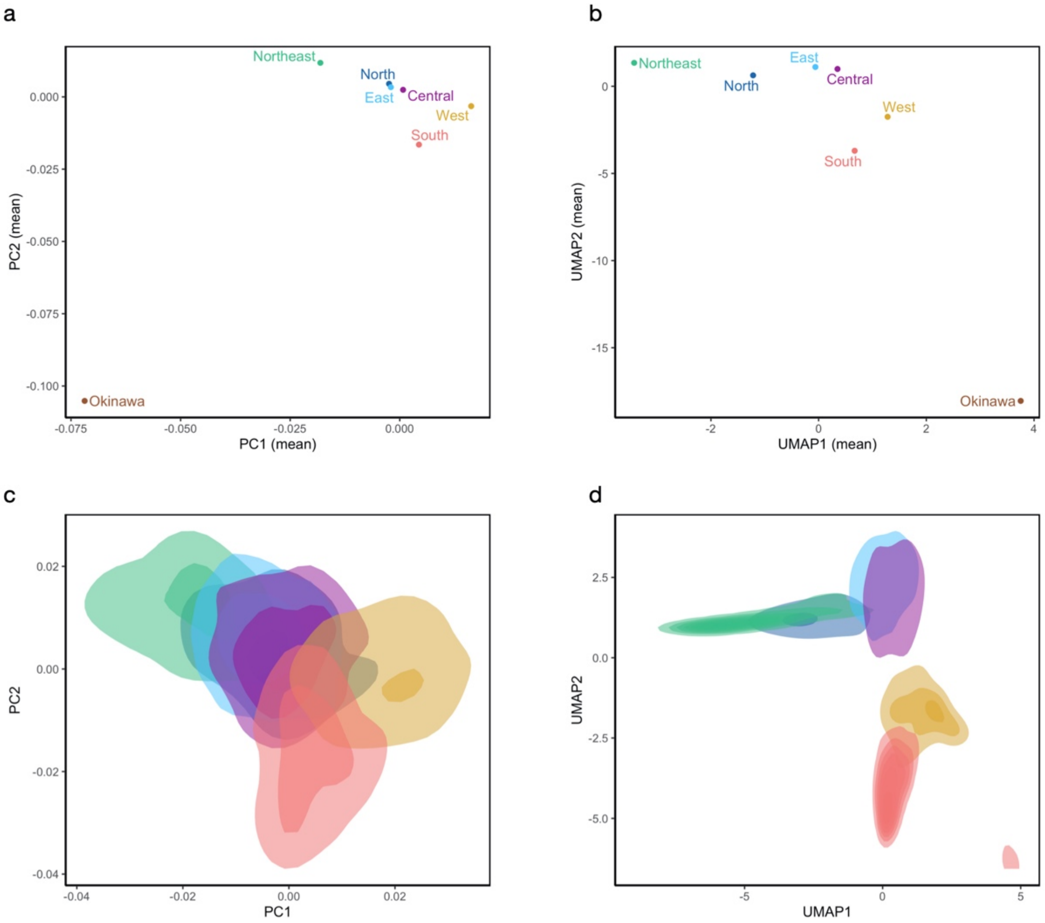

Fig. S1. PCA-UMAP analysis based on rare variants offers a more distinct separation of subjects from the Hondo region. a, b. The mean PCA and PCA-UMAP values grouped by regions. **c, d.** The contour plots depict the density distribution of PCA and PCA-UMAP values, respectively, for six Hondo regions (excluding Okinawa). The colors match those in Figure 1.

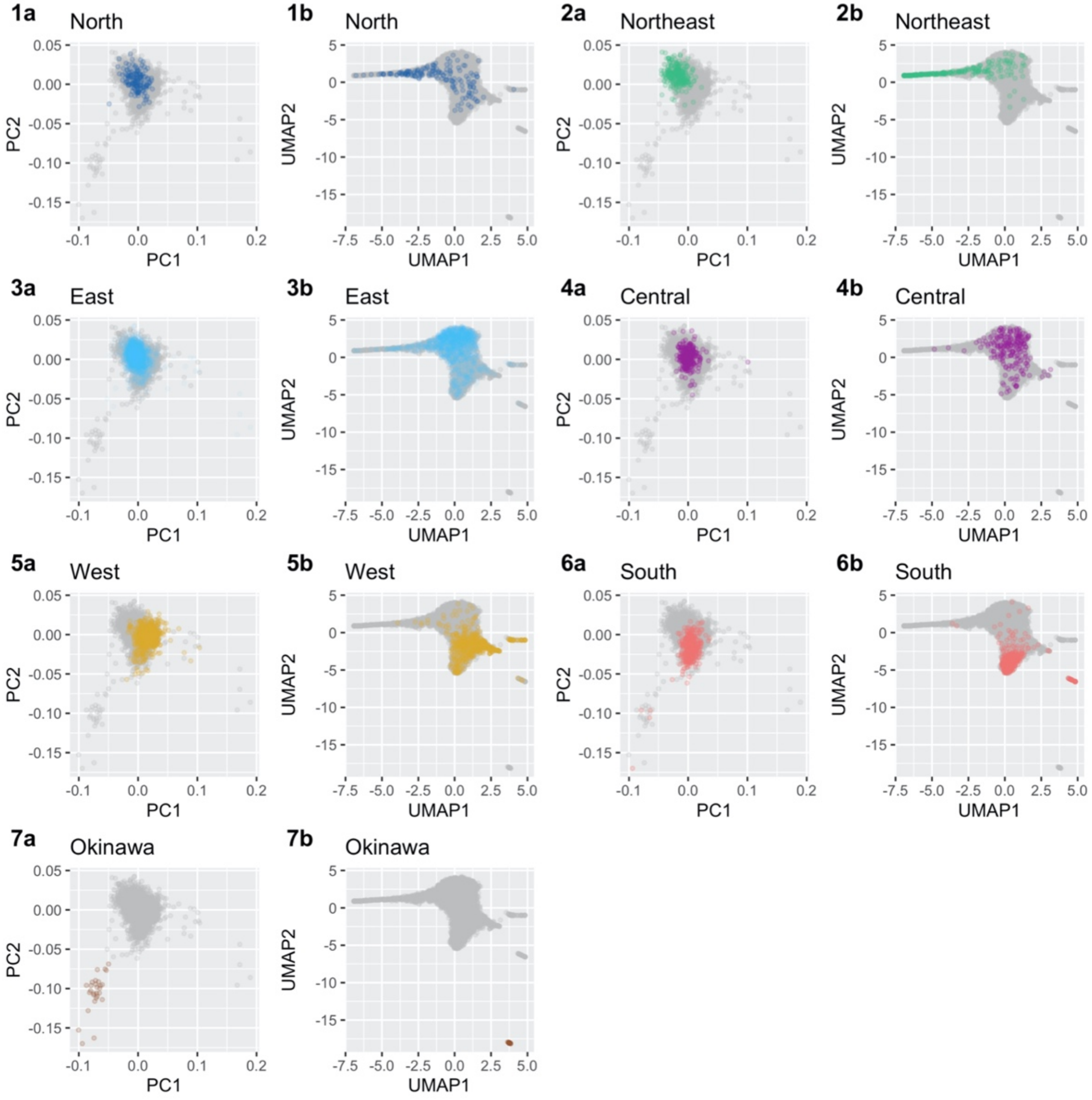

Fig. S2. PCA and PCA-UMAP for each region. 1a-7a. The PCA plots are displayed for the seven regions. In each plot, individuals from one of the seven regions are highlighted using the colors from Figure 1, while individuals from the other six regions are shaded in gray. **1b-7b**. Similarly, each region is highlighted in PCA-UMAP plots.

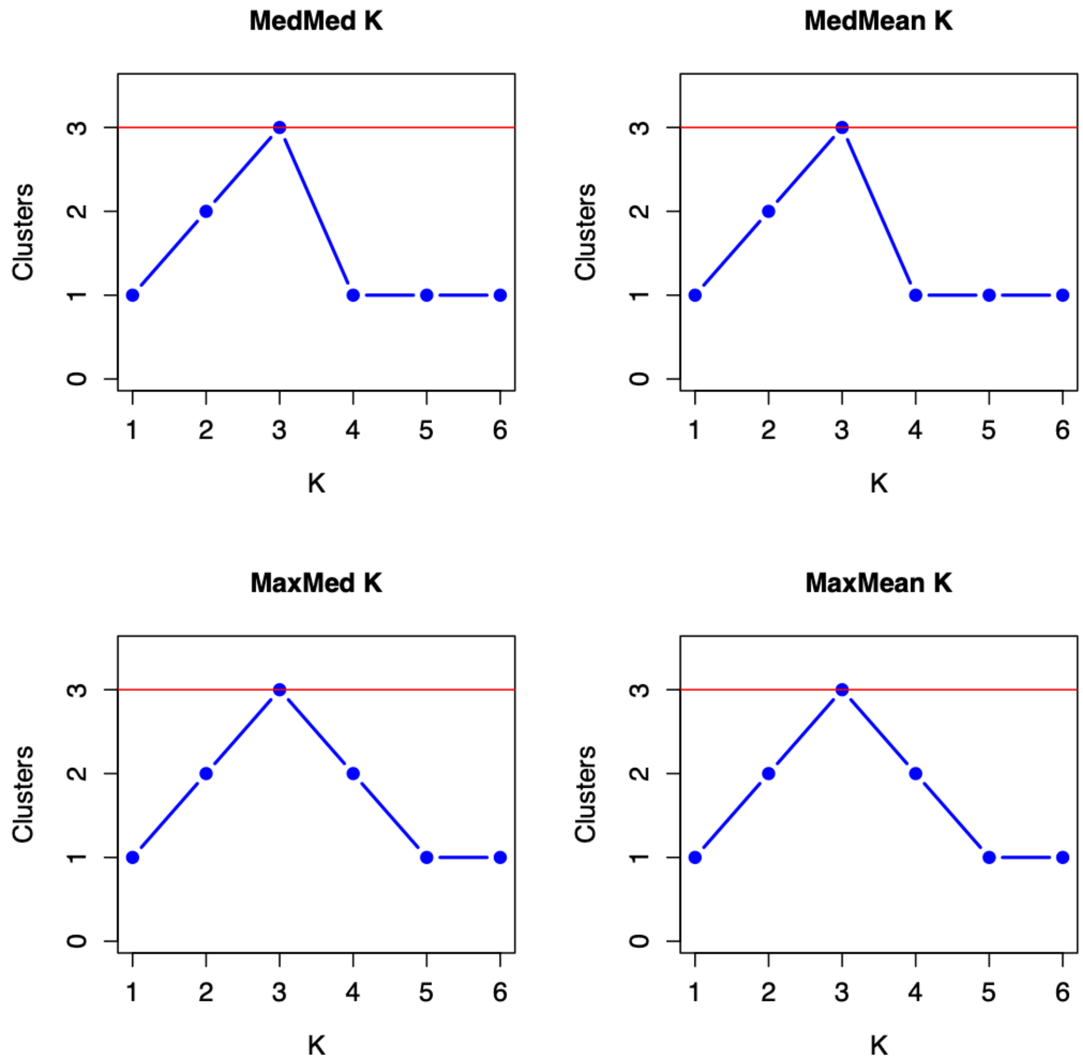

Fig. S3. Optimal K-value identification using statistics implemented in StructureSelector
Four statistics, namely MedMedK, MedMeaK, MaxMedK, and MaxMeaK, were employed to identify the optimal K value. The red horizontal line denotes the K value determined by each of the four statistics.

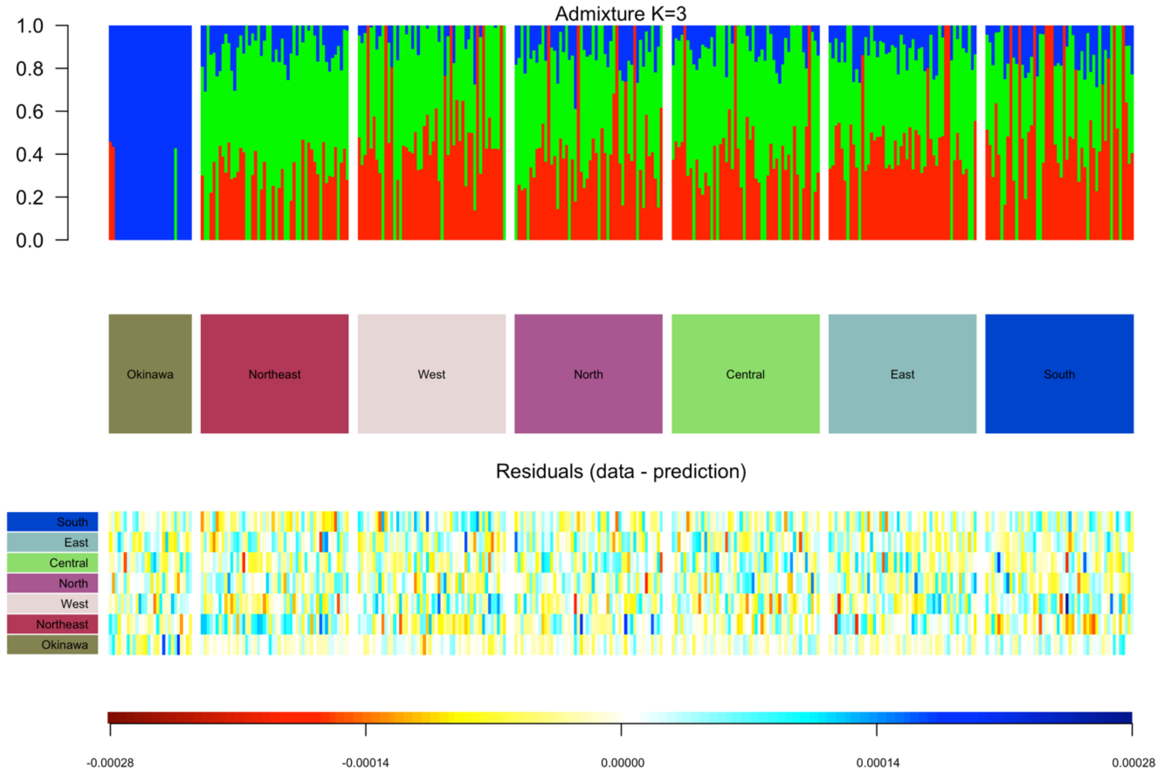

Fig. S4. Inspection of model fitting by badMIXTURE at K=3
For badMIXTURE analysis, we randomly selected 50 samples from each region, except for Okinawa, where all 28 samples were included. The upper panel displays the inferred ADMIXTURE plots at K=3, and the lower panel shows the residual palette under the best-fit ancestral population admixture model. No systematic pattern of residuals was observed.

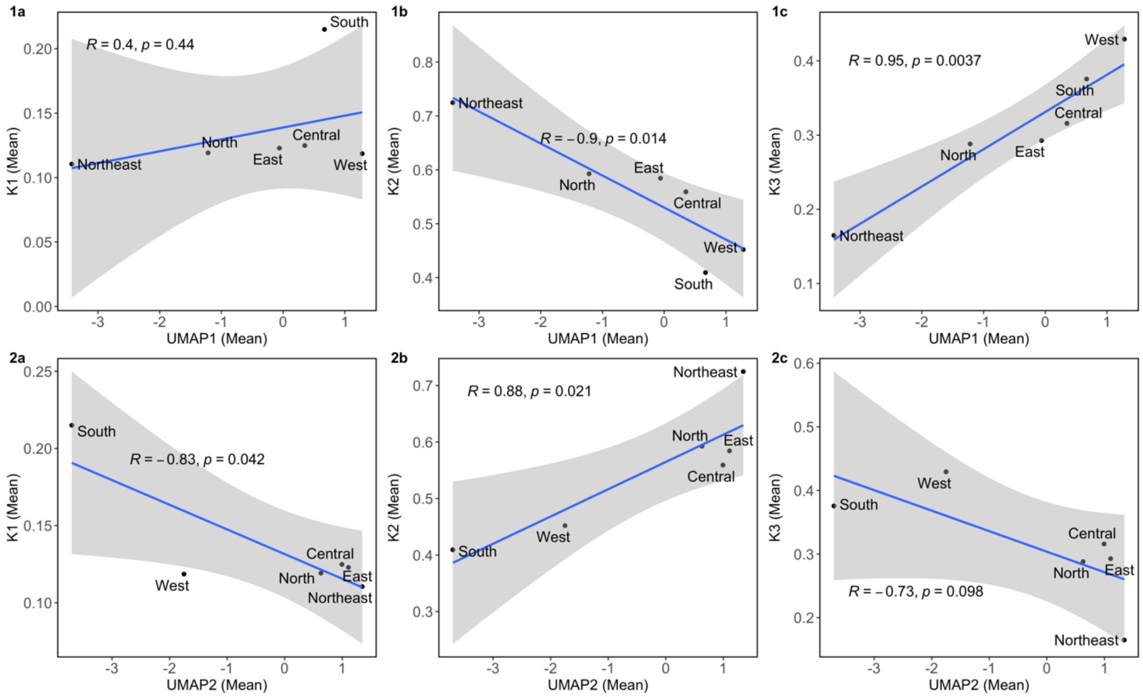

Fig. S5. Correlation between PCA-UMAP and ADMIXTURE K values.
1a-1c. The correlation between UMAP1 and K1-K3, summarized for each region excluding Okinawa. **2a-2c.** The correlation between UMAP2 and K1-K3, summarized for each region.

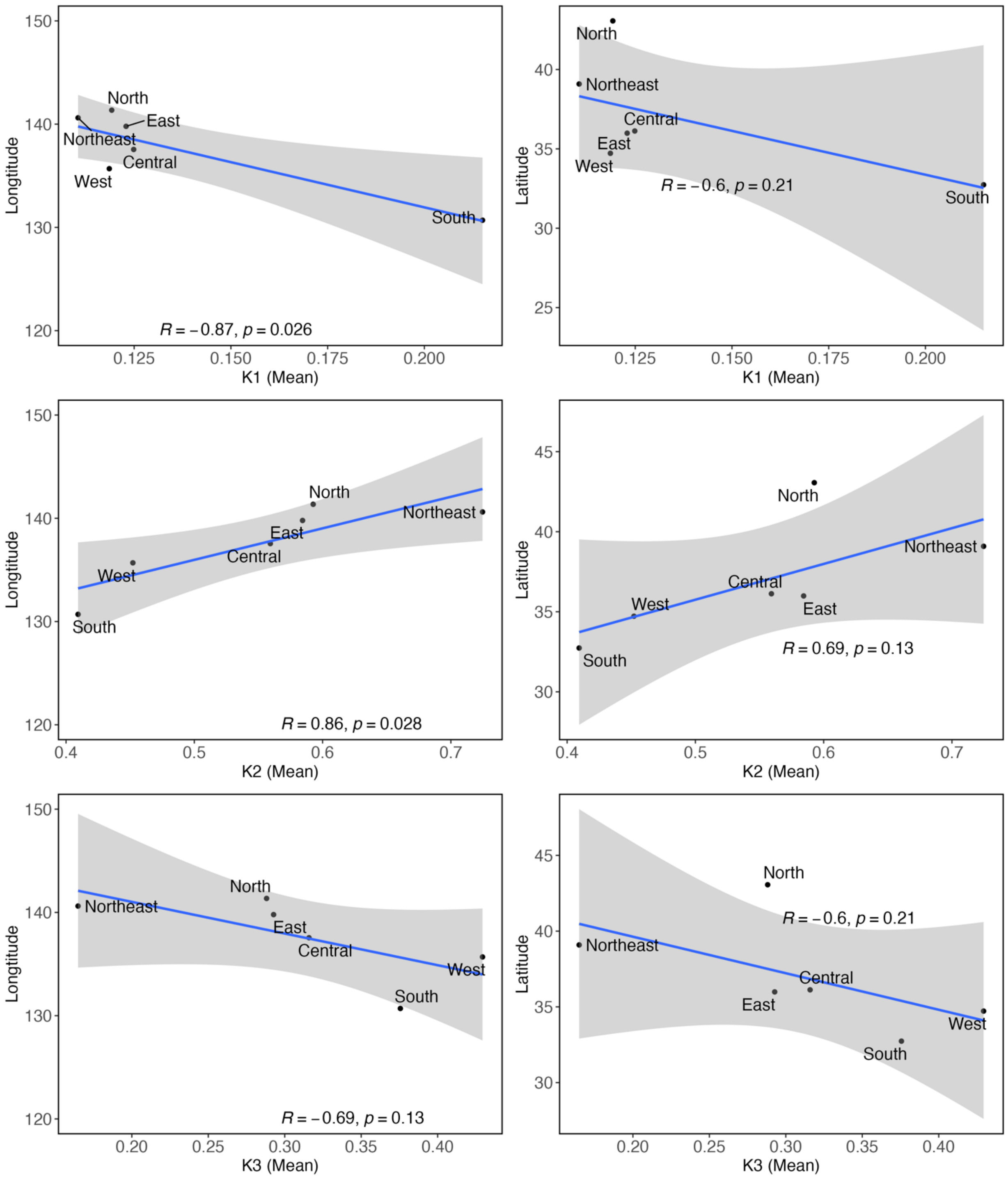

Fig. S6. Correlation between ADMIXTURE K values and geographical locations. Correlations between K1, K2, and K3 with longitude (1a, 2a, 3a) and latitude (1b, 2b, 3b). Significant correlations were observed for K1 and K2 with longitude, but not for K3. Okinawa samples were excluded from the analysis as they predominantly exhibited K1.

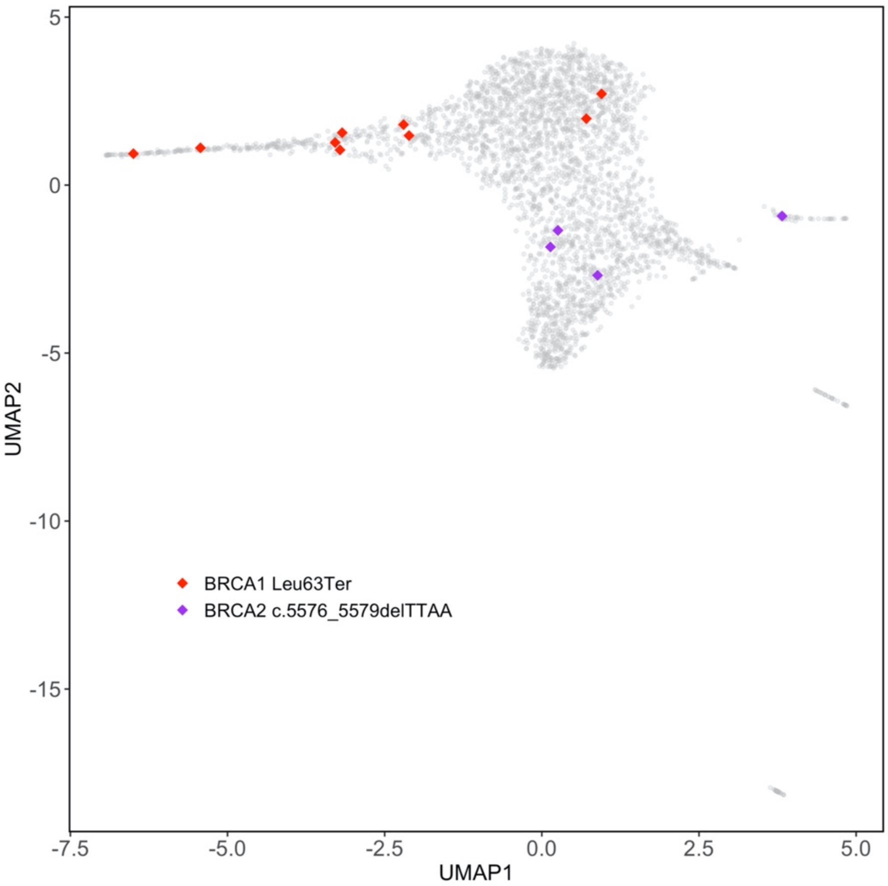

Fig. S7. The PCA-UMAP reveals the origins of two BRCA1/2 founder mutations.
The *BRCA1* Leu63Ter variant is enriched in subjects with likely northeastern ancestry, indicated by carriers marked in red. Additionally, four individuals with the *BRCA2* c.5576_5579delTTAA variant are marked in purple.

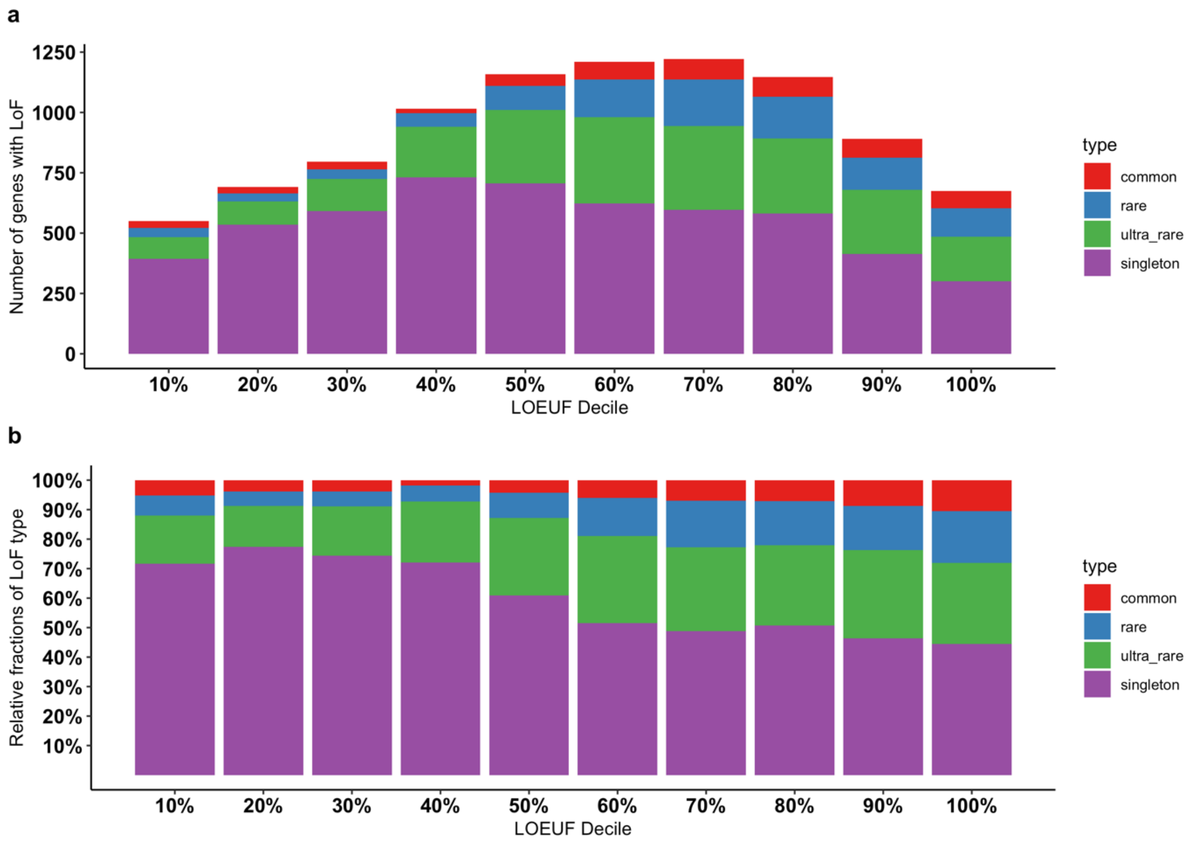

Fig. S8. Number of genes harboring a LoF variants by LOEUF decile.

a) Number of genes containing LoF variants for each decile, further divided by LoF types: Common (MAF (minor allele frequency) >= 0.01); Rare (0.001 <= MAF < 0.01); Ultra-rare (MAF < 0.001 & AC > 1); and Singleton. **b)** Relative fractions of LoF types. The data reveal that fewer genes in top LOEUF deciles contain LoF variants, and there is a higher proportion of singletons among LoF variants affecting genes in these top LOEUF deciles compared to those in the lower LOEUF deciles. Each LOEUF decile contains 1,920 genes, and genes in the top decile (e.g., top 10%) are highly intolerant to LoF mutations.

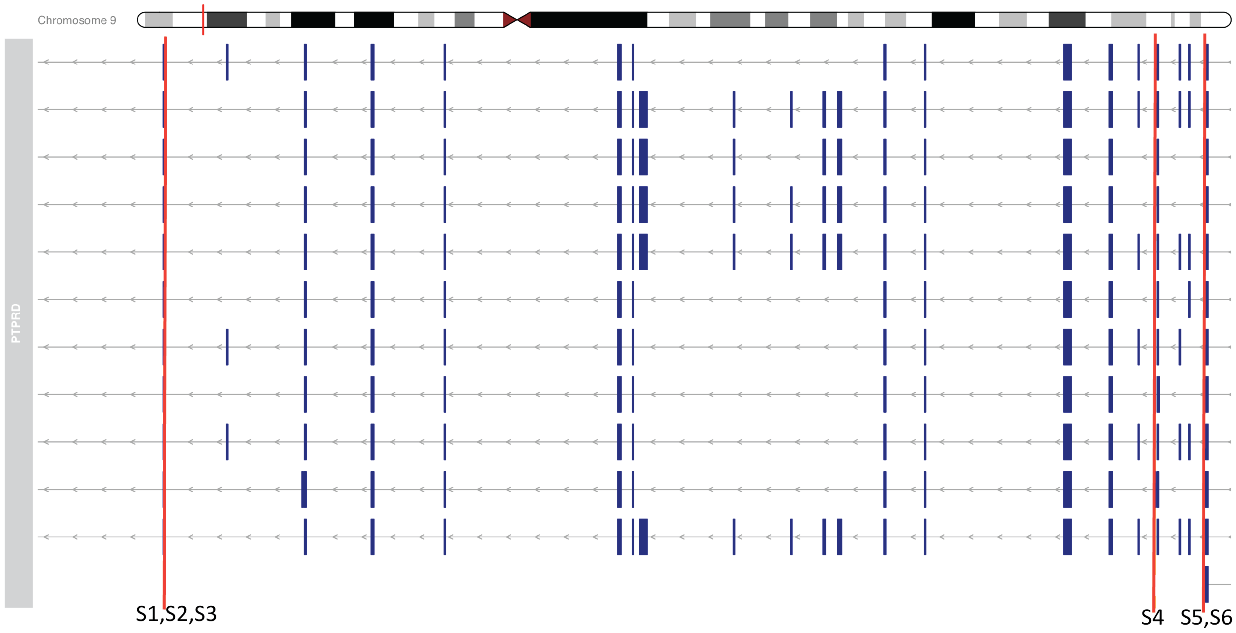

Fig. S9. LoF variants in *PTPRD* gene
The figure provides a zoomed-out view of the region from Fig 2.e, featuring 6 carriers (S1-S6). The yellow line indicates the location of the LoF variants.

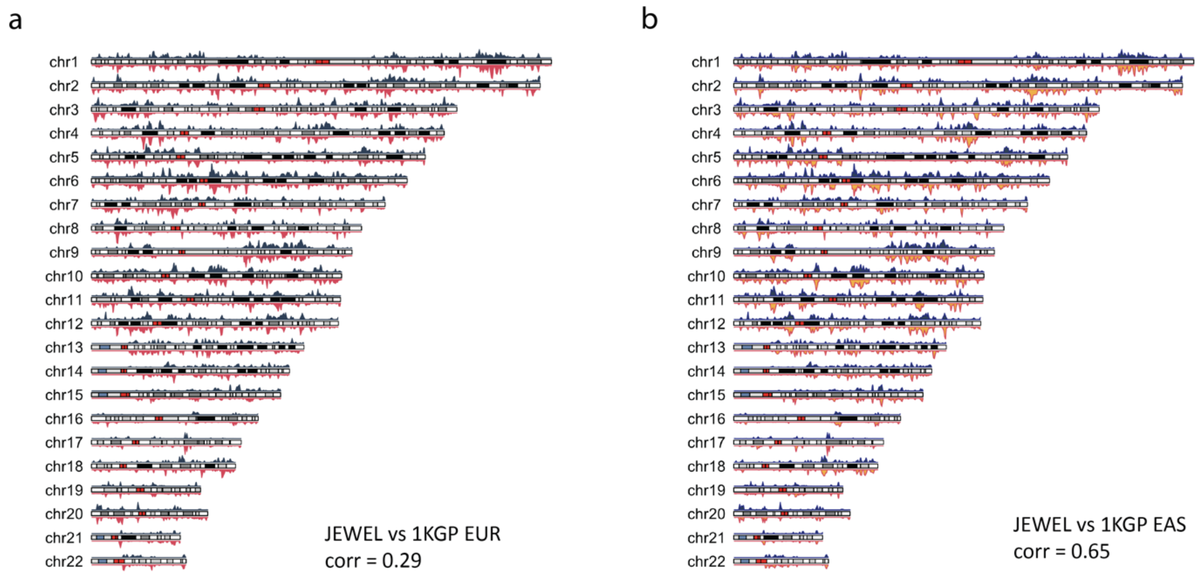

Fig. S10. Comparison of Identified Neanderthal Sequences Across Different Datasets. **a**. comparison of Neanderthal-introgressed sequences derived from the JEWEL dataset with those from the 1KGP European populations (EUR). **b** a similar comparison but with 1KGP East Asian populations (EAS). The analysis reveals a stronger correlation between JEWEL-identified introgressed regions in Japanese samples and the 1KGP EAS dataset (Pearson correlation = 0.65) compared to the 1KGP EUR dataset (Pearson correlation = 0.29).

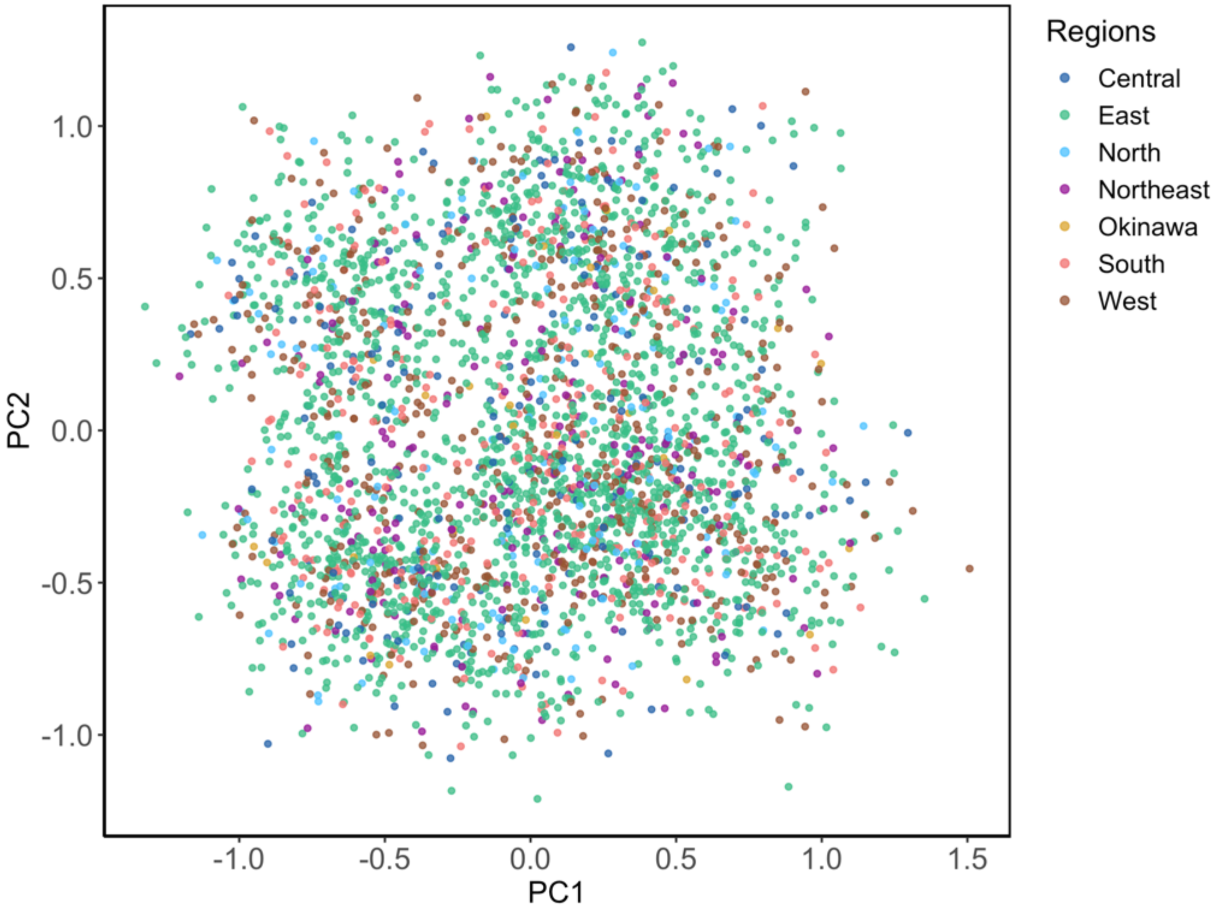

Fig. S11. PCA of the Neanderthal-introgressed segments

PCA was performed based on introgressed segments across 3,256 subjects. No sub-regional differences were observed in the analysis.

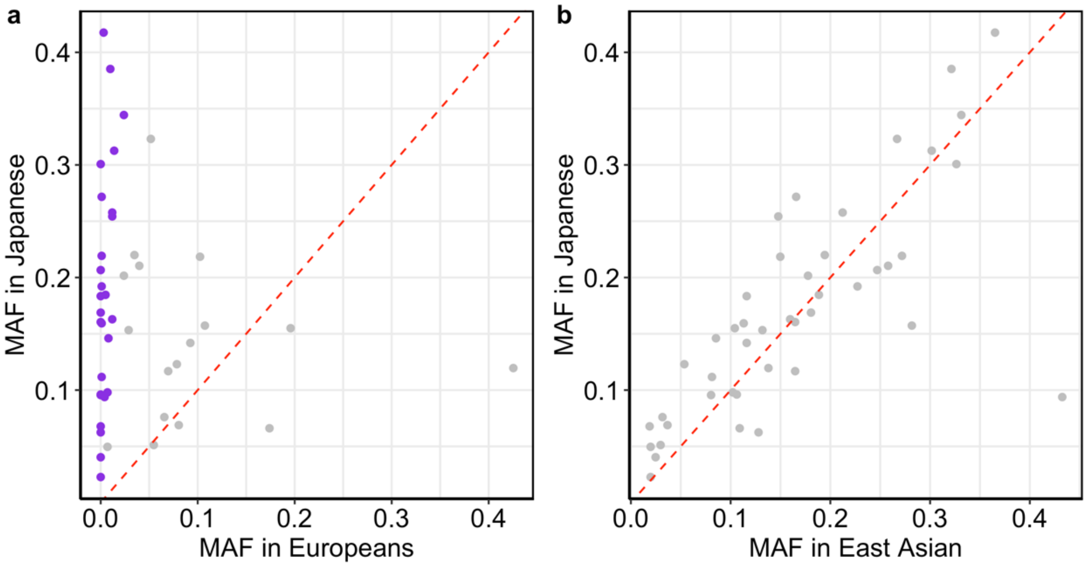

Fig. S12. Comparison of Minor Allele Frequencies (MAFs) for Lead Variants Within 44 Introgressed Segments Associated with Phenotypes. a) Between Japanese and 1KGP Europeans. **b)** Between Japanese and East Asians. Variants displaying more than a 10-fold higher MAF in the Japanese population are highlighted in purple. The findings suggest that the vast majority of lead variants within the 44 introgressed segments associated with phenotypes are specific to East Asians when compared to Europeans.

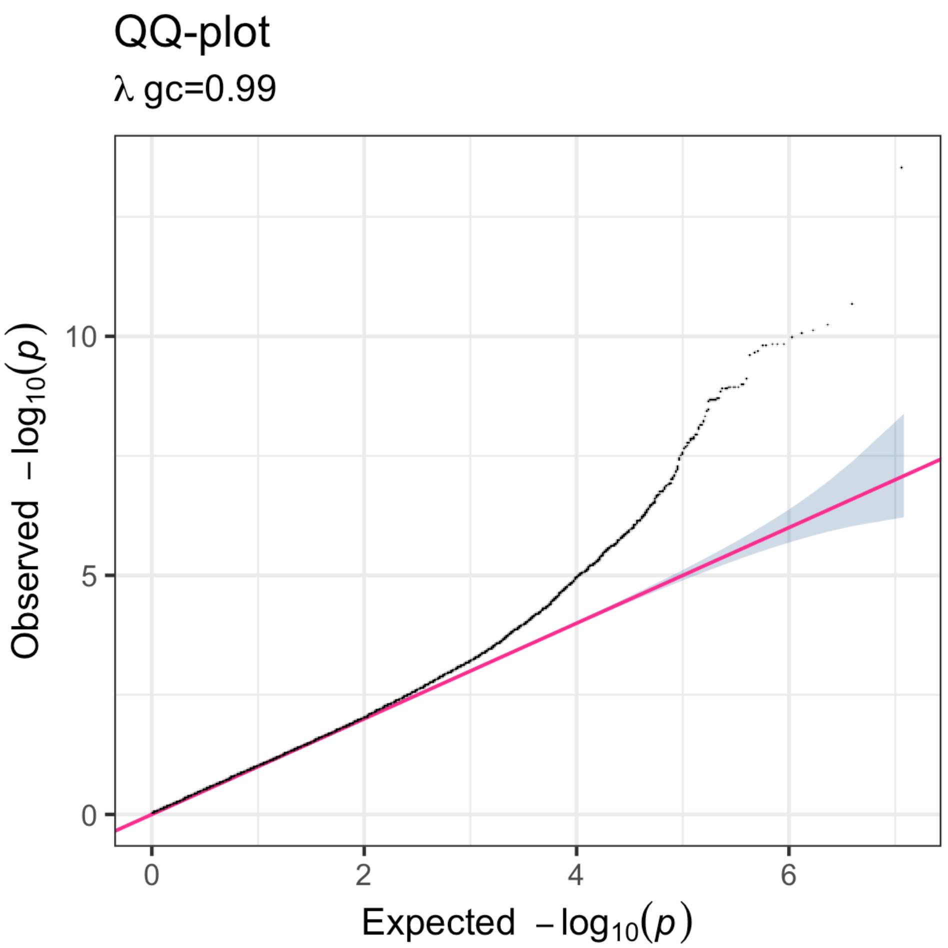

Fig. S13. Quantile-quantile (QQ) plot of iHS statistics in non-HLA regions
Comparison of observed approximate P_{iHS} values versus expected P values for all variants in non-HLA regions,

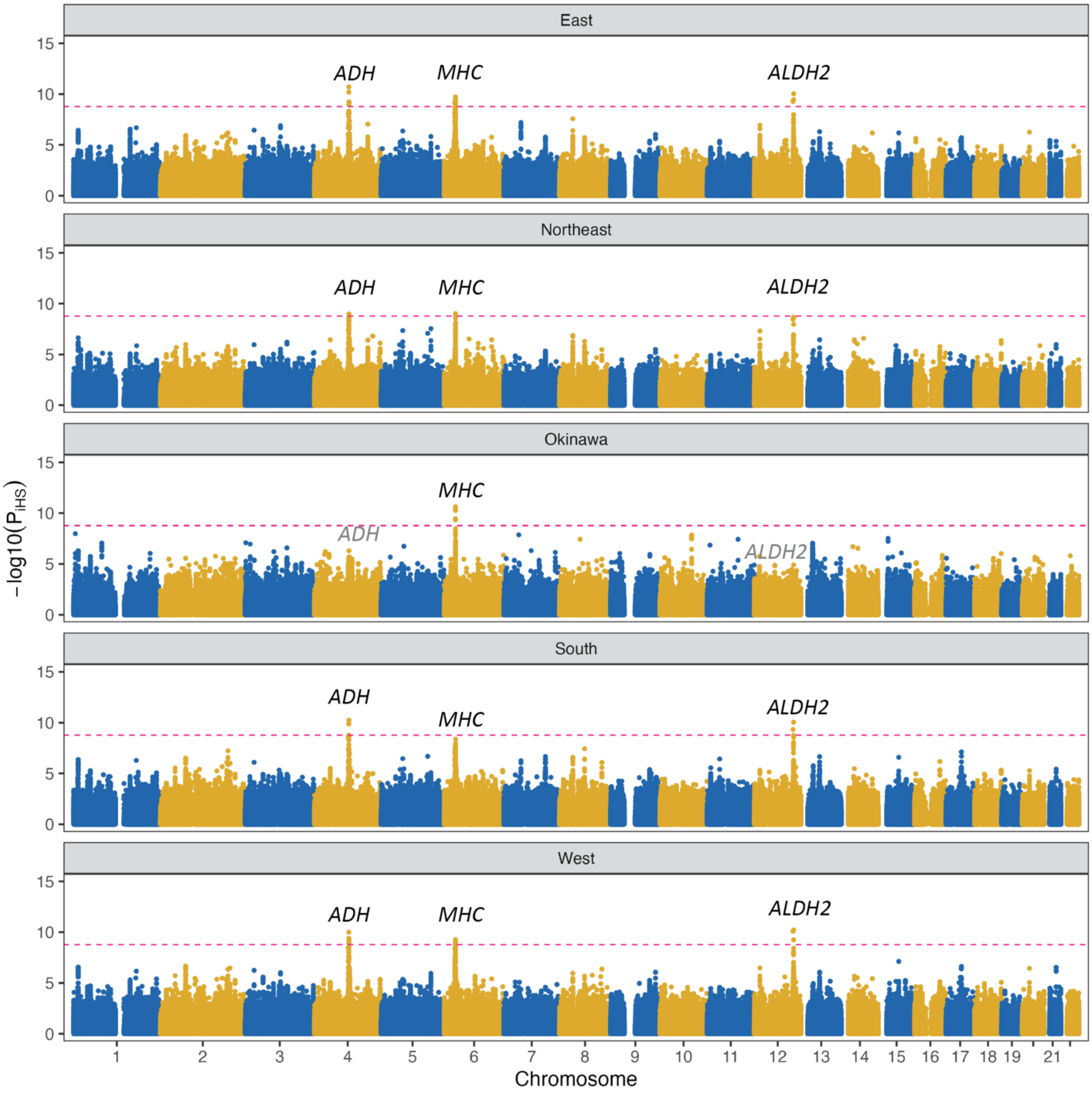

Fig. S14. iHS analysis for sub-regions.

The -log10(P_{iHS}) value (y-axis) and the chromosomal position (*x*-axis) of each SNP are plotted across the genome. The red dashed line indicates the Bonferroni-corrected genome-wide significance threshold (approximate $P_{iHS} < 8.24\times10^{-9}$). The *ADH* locus in Okinawa was marked as grey as it falls short of genome-wide significance.

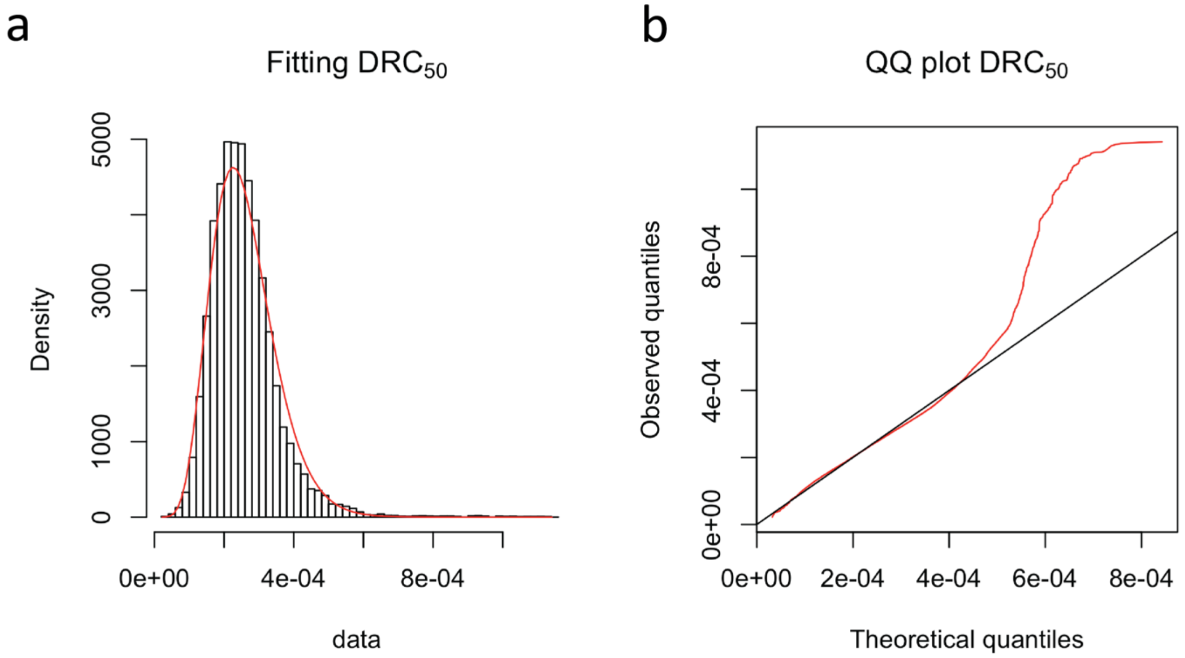

Fig. S15. Empirical null model for the DRC50 statistic.
a. the empirical distribution and Gamma fitting (red curve) for the DRC statistic were examined in the putative neutral regions of the genome. **b**. The quantile-quantile (QQ) plot for the DRC statistic

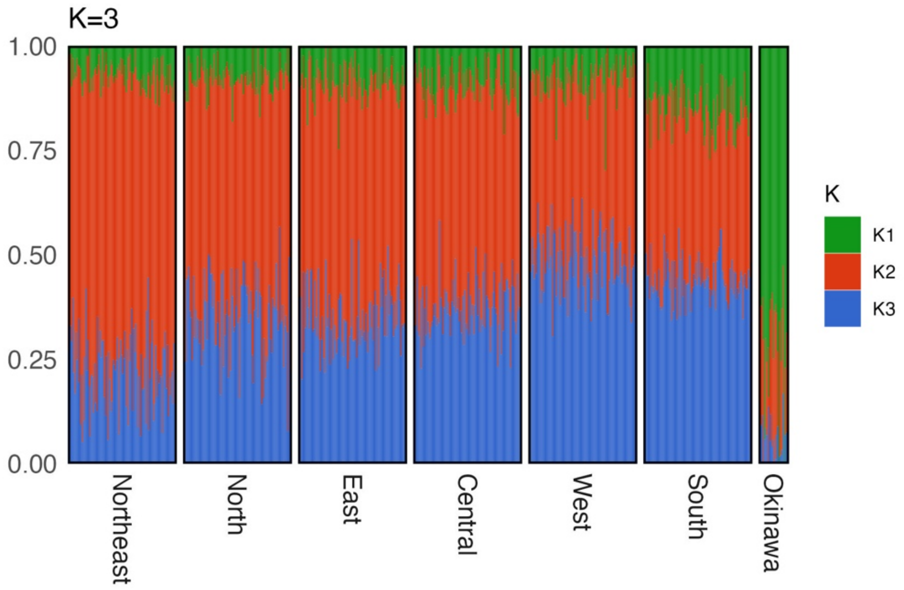

Fig. S16. ADMIXTURE analysis using both common and rare variants
ADMIXTURE analysis conducted using SCOPE software with K set to 3. Both common and rare pruned variants were included as input. The samples plotted are consistent with those presented in Fig. 1d.

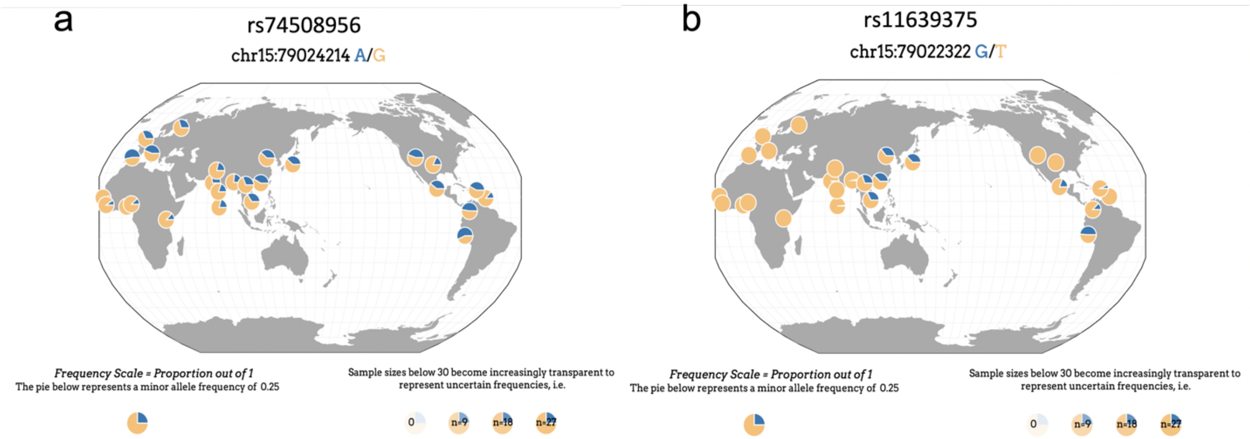

Fig. S17. Worldwide AF of rs74508956 and rs11639375

Worldwide geographic AF distribution for rs74508956 (a) and rs11639375 (b) generated by the Geography of Genetic Variants browser (https://popgen.uchicago.edu/ggv/). The source data is derived from the 1KGP dataset. Each pie chart represents a specific population; the blue slice indicates the frequency of the global minor allele.

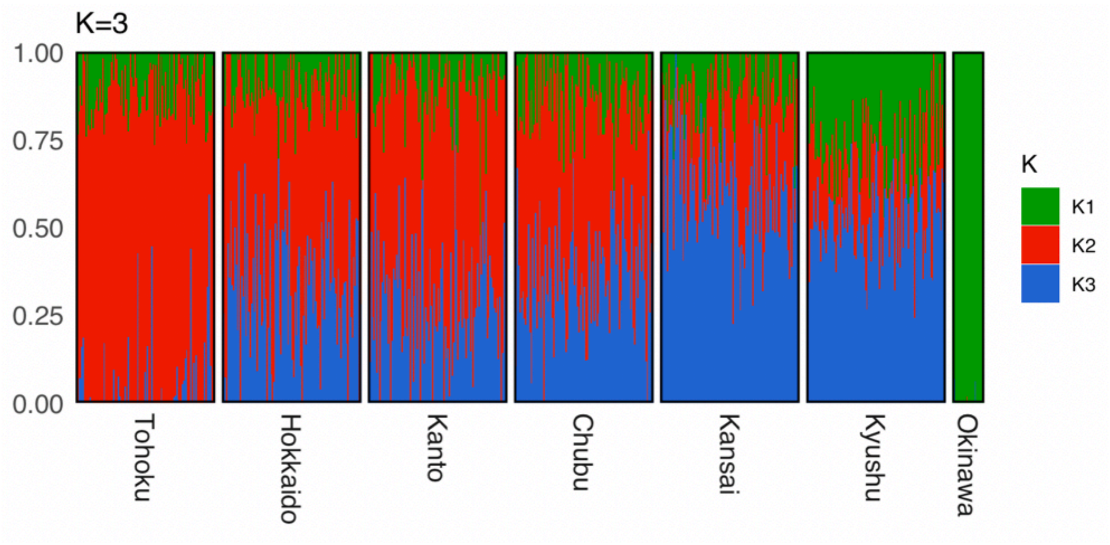

Fig. S18: ADMIXTURE data using array data
ADMIXTURE analysis was conducted using array data, adhering to the same protocol employed for the WGS dataset.

Supplementary Tables
(separate Excel file)

Table S1: Demographic characteristics of subjects in the JEWEL WGS dataset
Table S2: Statistics of biallelic SNPs/indels by chromosome in JEWEL
Table S3: Comparison of WGS quality metrics for samples sequenced at medium and high depth
Table S4: Average ADMIXTURE K values grouped by subjects' region
Table S5: Mixture proportions of CHB and Jomon ancestry estimated with f_4 ratio analysis
Table S6: Outgroup-f_3 statistics to examine the affinity between JEWEL subgroup and Han Chinese
Table S7: f_4-Statistics in form f_4(Mbuti, ancient genome; Northeast, West)
Table S8: qpAdm results of three-way model consisting NEA, EA, and Jomon, Korea-TK_2, China_YR_MN, and China_WLR_BA
Table S9: Results of qpAdm two-way model analysis with combinations of Korea-TK_2, NEA, Han, Jomon, and China_YR_MN
Table S10: Association between the ancestry K and quantitative QTL traits
Table S11: Details of putative loss-of-function (LoF) variants discovered in the JEWEL dataset
Table S12: Loss-of-function variants significantly associated with UMAP1/2
Table S13: Number of LoF, missense, and synonymous variants per individual by region
Table S14: Coding variants per individual by region based on random resampling analysis
Table S15: Mean missense risk score by allele frequency category
Table S16: Identified human knockouts in JEWEL dataset
Table S17: Clinical phenotypes of heterozygous carriers of LoF variants in *PTPRD*
Table S18: Genes with significantly fewer transcripts affected by LoF variants than expected
Table S19: Average total length of introgressed segments (Neanderthal or Denisovan) for individuals grouped by regions
Table S20: Enrichment of Denisovan segments across populations
Table S21: Introgressed variants protective for coronary artery disease (CAD)
Table S22: Introgressed segments associated with quantitative traits in the Japanese Population
Table S23: Significant loci under positive selection detected by iHS analysis for each sub-region
Table S24: Comparison of average ADMIXTURE K values after removal of 147 SNPs in problematic regions

REFERENCES AND NOTES

1. O. Bocher, C. J. Willer, E. Zeggini, Unravelling the genetic architecture of human complex traits through whole genome sequencing. *Nat. Commun.* **14**, 3520 (2023).

2. H. Jónsson, P. Sulem, B. Kehr, S. Kristmundsdottir, F. Zink, E. Hjartarson, M. T. Hardarson, K. E. Hjorleifsson, H. P. Eggertsson, S. A. Gudjonsson, L. D. Ward, G. A. Arnadottir, E. A. Helgason, H. Helgason, A. Gylfason, A. Jonasdottir, A. Jonasdottir, T. Rafnar, M. Frigge, S. N. Stacey, O. Th. Magnusson, U. Thorsteinsdottir, G. Masson, A. Kong, B. V. Halldorsson, A. Helgason, D. F. Gudbjartsson, K. Stefansson, Parental influence on human germline de novo mutations in 1,548 trios from Iceland. *Nature* **549**, 519–522 (2017).

3. S. Mallick, H. Li, M. Lipson, I. Mathieson, M. Gymrek, F. Racimo, M. Zhao, N. Chennagiri, S. Nordenfelt, A. Tandon, P. Skoglund, I. Lazaridis, S. Sankararaman, Q. Fu, N. Rohland, G. Renaud, Y. Erlich, T. Willems, C. Gallo, J. P. Spence, Y. S. Song, G. Poletti, F. Balloux, G. Van Driem, P. De Knijff, I. G. Romero, A. R. Jha, D. M. Behar, C. M. Bravi, C. Capelli, T. Hervig, A. Moreno-Estrada, O. L. Posukh, E. Balanovska, O. Balanovsky, S. Karachanak-Yankova, H. Sahakyan, D. Toncheva, L. Yepiskoposyan, C. Tyler-Smith, Y. Xue, M. S. Abdullah, A. Ruiz-Linares, C. M. Beall, A. Di Rienzo, C. Jeong, E. B. Starikovskaya, E. Metspalu, J. Parik, R. Villems, B. M. Henn, U. Hodoglugil, R. Mahley, A. Sajantila, G. Stamatoyannopoulos, J. T. S. Wee, R. Khusainova, E. Khusnutdinova, S. Litvinov, G. Ayodo, D. Comas, M. F. Hammer, T. Kivisild, W. Klitz, C. A. Winkler, D. Labuda, M. Bamshad, L. B. Jorde, S. A. Tishkoff, W. S. Watkins, M. Metspalu, S. Dryomov, R. Sukernik, L. Singh, K. Thangaraj, S. Pääbo, J. Kelso, N. Patterson, D. Reich, The Simons Genome Diversity Project: 300 genomes from 142 diverse populations. *Nature* **538**, 201–206 (2016).

4. J. Choin, J. Mendoza-Revilla, L. R. Arauna, S. Cuadros-Espinoza, O. Cassar, M. Larena, A. M.-S. Ko, C. Harmant, R. Laurent, P. Verdu, G. Laval, A. Boland, R. Olaso, J.-F. Deleuze, F. Valentin, Y.-C. Ko, M. Jakobsson, A. Gessain, L. Excoffier, M. Stoneking, E. Patin, L. Quintana-Murci, Genomic insights into population history and biological adaptation in Oceania. *Nature* **592**, 583–589 (2021).

5. Y. Field, E. A. Boyle, N. Telis, Z. Gao, K. J. Gaulton, D. Golan, L. Yengo, G. Rocheleau, P. Froguel, M. I. McCarthy, J. K. Pritchard, Detection of human adaptation during the past 2000 years. *Science* **354**, 760–764 (2016).

6. K. E. Johnson, B. F. Voight, Patterns of shared signatures of recent positive selection across human populations. *Nat. Ecol. Evol.* **2**, 713–720 (2018).

7. S. M. Carthy, S. Das, W. Kretzschmar, O. Delaneau, A. R. Wood, A. Teumer, H. M. Kang, C. Fuchsberger, P. Danecek, K. Sharp, Y. Luo, C. Sidore, A. Kwong, N. Timpson, S. Koskinen, S. Vrieze, L. J. Scott, H. Zhang, A. Mahajan, J. Veldink, U. Peters, C. Pato, C. M. van Duijn, C. E. Gillies, I. Gandin, M. Mezzavilla, A. Gilly, M. Cocca, M. Traglia, A. Angius, J. Barrett, D. I. Boomsma, K. Branham, G. Breen, C. Brummet, F. Busonero, H. Campbell, A. Chan, S. Chen, E. Chew, F. S. Collins, L. Corbin, G. D. Smith, G. Dedoussis, M. Dorr, A.-E. Farmaki, L. Ferrucci, L. Forer, R. M. Fraser, S. Gabriel, S. Levy, L. Groop, T. Harrison, A. Hattersley, O. L. Holmen, K. Hveem, M. Kretzler, J. Lee, M. M. Gue, T. Meitinger, D. Melzer, J. Min, K. L. Mohlke, J. Vincent, M. Nauck, D. Nickerson, A. Palotie, M. Pato, N. Pirastu, M. M. Innis, B. Richards, C. Sala, V. Salomaa, D. Schlessinger, S. Schoenheer, P Eline Slagboom, K. Small, T. Spector, D. Stambolian, M. Tuke, J. Tuomilehto, L. Van den Berg, W. Van Rheenen, U. Volker, C. Wijmenga, D. Toniolo, E. Zeggini, P. Gasparini, M. G. Sampson, J. F. Wilson, T. Frayling, P. de Bakker, M. A. Swertz, S. M. Carroll, C. Kooperberg, A. Dekker, D. Altshuler, C. Willer, W. Iacono, S. Ripatti, N. Soranzo, K. Walter, A. Swaroop, F. Cucca, C. Anderson, M. Boehnke, Mark I. Mc Carthy, R. Durbin, G. Abecasis, J. Marchini, for the Haplotype Reference Consortium; A reference panel of 64,976 haplotypes for genotype imputation. *Nat. Genet.* **48**, 1279–1283 (2016).

8. S.-K. Yoo, C.-U. Kim, H. L. Kim, S. Kim, J.-Y. Shin, N. Kim, J. S. W. Yang, K.-W. Lo, B. Cho, F. Matsuda, S. C. Schuster, C. Kim, J.-I. Kim, J.-S. Seo, NARD: Whole-genome reference panel of 1779 Northeast Asians improves imputation accuracy of rare and low-frequency variants. *Genome Med.* **11**, 64 (2019).

9. S. Das, G. R. Abecasis, B. L. Browning, Genotype imputation from large reference panels. *Annu. Rev. Genomics Hum. Genet.* **19**, 73–96 (2018).

10. The UK10K Consortium; The UK10K project identifies rare variants in health and disease. *Nature* **526**, 82–90 (2015).

11. F. S. Alkuraya, Natural human knockouts and the era of genotype to phenotype. *Genome Med.* **7**, 48 (2015).

12. E. V. Minikel, K. J. Karczewski, H. C. Martin, B. B. Cummings, N. Whiffin, D. Rhodes, J. Alföldi, R. C. Trembath, D. A. van Heel, M. J. Daly, Genome Aggregation Database Production Team; Genome Aggregation Database Consortium; S. L. Schreiber, D. G. MacArthur, Evaluating drug targets through human loss-of-function genetic variation. *Nature* **581**, 459–464 (2020).

13. B. V. Halldorsson, H. P. Eggertsson, K. H. S. Moore, H. Hauswedell, O. Eiriksson, M. O. Ulfarsson, G. Palsson, M. T. Hardarson, A. Oddsson, B. O. Jensson, S. Kristmundsdottir, B. D. Sigurpalsdottir, O. A. Stefansson, D. Beyter, G. Holley, V. Tragante, A. Gylfason, P. I. Olason, F. Zink, M. Asgeirsdottir, S. T. Sverrisson, B. Sigurdsson, S. A. Gudjonsson, G. T. Sigurdsson, G. H. Halldorsson, G. Sveinbjornsson, K. Norland, U. Styrkarsdottir, D. N. Magnusdottir, S. Snorradottir, K. Kristinsson, E. Sobech, H. Jonsson, A. J. Geirsson, I. Olafsson, P. Jonsson, O. B. Pedersen, C. Erikstrup, S. Brunak, S. R. Ostrowski, DBDS Genetic Consortium, G. Thorleifsson, F. Jonsson, P. Melsted, I. Jonsdottir, T. Rafnar, H. Holm, H. Stefansson, J. Saemundsdottir, D. F. Gudbjartsson, O. T. Magnusson, G. Masson, U. Thorsteinsdottir, A. Helgason, H. Jonsson, P. Sulem, K. Stefansson, The sequences of 150,119 genomes in the UK Biobank. *Nature* **607**, 732–740 (2022).

14. M. I. Kurki, J. Karjalainen, P. Palta, T. P. Sipilä, K. Kristiansson, K. M. Donner, M. P. Reeve, H. Laivuori, M. Aavikko, M. A. Kaunisto, A. Loukola, E. Lahtela, H. Mattsson, P. Laiho, P. D. B. Parolo, A. A. Lehisto, M. Kanai, N. Mars, J. Rämö, T. Kiiskinen, H. O. Heyne, K. Veerapen, S. Rüeger, S. Lemmelä, W. Zhou, S. Ruotsalainen, K. Pärn, T. Hiekkalinna, S. Koskelainen, T. Paajanen, V. Llorens, J. Gracia-Tabuenca, H. Siirtola, K. Reis, A. G. Elnahas, B. Sun, C. N. Foley, K. Aalto-Setälä, K. Alasoo, M. Arvas, K. Auro, S. Biswas, A. Bizaki-Vallaskangas, O. Carpen, C.-Y. Chen, O. A. Dada, Z. Ding, M. G. Ehm, K. Eklund, M. Färkkilä, H. Finucane, A. Ganna, A. Ghazal, R. R. Graham, E. M. Green, A. Hakanen, M. Hautalahti, Å. K. Hedman, M. Hiltunen, R. Hinttala, I. Hovatta, X. Hu, A. Huertas-Vazquez, L. Huilaja, J. Hunkapiller, H. Jacob, J.-N. Jensen, H. Joensuu, S. John, V. Julkunen, M. Jung, J. Junttila, K. Kaarniranta, M. Kähönen, R. Kajanne, L. Kallio, R. Kälviäinen, J. Kaprio, F. Gen, N. Kerimov, J. Kettunen, E. Kilpeläinen, T. Kilpi, K. Klinger, V.-M. Kosma, T. Kuopio, V. Kurra, T. Laisk, J. Laukkanen, N. Lawless, A. Liu, S. Longerich, R. Mägi, J. Mäkelä, A. Mäkitie, A. Malarstig, A. Mannermaa, J. Maranville, A. Matakidou, T. Meretoja, S. V. Mozaffari, M. E. K. Niemi, M. Niemi, T. Niiranen, C. J. O. Donnell, M. E. Obeidat, G. Okafo, H. M. Ollila, A. Palomäki, T. Palotie, J. Partanen, D. S. Paul, M. Pelkonen, R. K. Pendergrass, S. Petrovski, A. Pitkäranta, A. Platt, D. Pulford, E. Punkka, P. Pussinen, N. Raghavan, F. Rahimov, D. Rajpal, N. A. Renaud, B. Riley-Gillis, R. Rodosthenous, E.

Saarentaus, A. Salminen, E. Salminen, V. Salomaa, J. Schleutker, R. Serpi, H.-Y. Shen, R. Siegel, K. Silander, S. Siltanen, S. Soini, H. Soininen, J. H. Sul, I. Tachmazidou, K. Tasanen, P. Tienari, S. Toppila-Salmi, T. Tukiainen, T. Tuomi, J. A. Turunen, J. C. Ulirsch, F. Vaura, P. Virolainen, J. Waring, D. Waterworth, R. Yang, M. Nelis, A. Reigo, A. Metspalu, L. Milani, T. Esko, C. Fox, A. S. Havulinna, M. Perola, S. Ripatti, A. Jalanko, T. Laitinen, T. P. Mäkelä, R. Plenge, M. M. Carthy, H. Runz, M. J. Daly, A. Palotie, FinnGen provides genetic insights from a well-phenotyped isolated population. *Nature* **613**, 508–518 (2023).

15. D. F. Gudbjartsson, H. Helgason, S. A. Gudjonsson, F. Zink, A. Oddson, A. Gylfason, S. Besenbacher, G. Magnusson, B. V. Halldorsson, E. Hjartarson, G. T. Sigurdsson, S. N. Stacey, M. L. Frigge, H. Holm, J. Saemundsdottir, H. T. Helgadottir, H. Johannsdottir, G. Sigfusson, G. Thorgeirsson, J. T. Sverrisson, S. Gretarsdottir, G. B. Walters, T. Rafnar, B. Thjodleifsson, E. S. Bjornsson, S. Olafsson, H. Thorarinsdottir, T. Steingrimsdottir, T. S. Gudmundsdottir, A. Theodors, J. G. Jonasson, A. Sigurdsson, G. Bjornsdottir, J. J. Jonsson, O. Thorarensen, P. Ludvigsson, H. Gudbjartsson, G. I. Eyjolfsson, O. Sigurdardottir, I. Olafsson, D. O. Arnar, O. T. Magnusson, A. Kong, G. Masson, U. Thorsteinsdottir, A. Helgason, P. Sulem, K. Stefansson, Large-scale whole-genome sequencing of the Icelandic population. *Nat. Genet.* **47**, 435–444 (2015).

16. A. B. Popejoy, S. M. Fullerton, Genomics is failing on diversity. *Nature* **538**, 161–164 (2016).

17. A. R. Martin, M. Kanai, Y. Kamatani, Y. Okada, B. M. Neale, M. J. Daly, Clinical use of current polygenic risk scores may exacerbate health disparities. *Nat. Genet.* **51**, 584–591 (2019).

18. The All of Us Research Program Investigators, The "All of Us" research program. *N. Engl. J. Med.* **381**, 668–676 (2019).

19. D. Taliun, D. N. Harris, M. D. Kessler, J. Carlson, Z. A. Szpiech, R. Torres, S. A. Gagliano Taliun, A. Corvelo, S. M. Gogarten, H. M. Kang, A. N. Pitsillides, J. L. Faive, S.-B. Lee, X. Tian, B. L. Browning, S. Das, A.-K. Emde, W. E. Clarke, D. P. Loesch, A. C. Shetty, T. W. Blackwell, A. V. Smith, Q. Wong, X. Liu, M. P. Conomos, D. M. Bobo, F. Aguet, C. Albert, A. Alonso, K. G. Ardlie, D. E. Arking, S. Aslibekyan, P. L. Auer, J. Barnard, R Graham Barr, L. Barwick, L. C. Becker, R. L. Beer, E. J. Benjamin, L. F. Bielak, J. Blangero, M. Boehnke, D. W. Bowden, J. A. Brody, E. G. Burchard, B. E. Cade, J. F. Casella, B. Chalazan, D. I. Chasman, Yii-Der Ida Chen, M. H. Cho, S. H. Choi, M. K.

Chung, C. B. Clish, A. Correa, J. E. Curran, B. Custer, D. Darbar, M. Daya, M. de Andrade, D. L. De Meo, S. K. Dutcher, P. T. Ellinor, L. S. Emery, C. Eng, D. Fatkin, T. Fingerlin, L. Forer, M. Fornage, N. Franceschini, C. Fuchsberger, S. M. Fullerton, S. Germer, M. T. Gladwin, D. J. Gottlieb, X. Guo, M. E. Hall, J. He, N. L. Heard-Costa, S. R. Heckbert, M. R. Irvin, J. M. Johnsen, A. D. Johnson, R. Kaplan, S. L. R. Kardia, T. Kelly, S. Kelly, E. E. Kenny, D. P. Kiel, R. Klemmer, B. A. Konkle, C. Kooperberg, A. Köttgen, L. A. Lange, J. Lasky-Su, D. Levy, X. Lin, K.-H. Lin, C. Liu, R. J. F. Loos, L. Garman, R. Gerszten, S. A. Lubitz, K. L. Lunetta, A. C. Y. Mak, A. Manichaikul, A. K. Manning, R. A. Mathias, D. D. Mc Manus, S. T. Mc Garvey, J. B. Meigs, D. A. Meyers, J. L. Mikulla, M. A. Minear, B. D. Mitchell, S. Mohanty, M. E. Montasser, C. Montgomery, A. C. Morrison, J. M. Murabito, A. Natale, P. Natarajan, S. C. Nelson, K. E. North, Jeffrey R O'Connell, N. D. Palmer, N. Pankratz, G. M. Peloso, P. A. Peyser, J. Pleiness, W. S. Post, B. M. Psaty, D C Rao, S. Redline, A. P. Reiner, D. Roden, J. I. Rotter, I. Ruczinski, C. Sarnowski, S. Schoenherr, D. A. Schwartz, J.-S. Seo, S. Seshadri, V. A. Sheehan, W. H. Sheu, M Benjamin Shoemaker, N. L. Smith, J. A. Smith, N. Sotoodehnia, A. M. Stilp, W. Tang, K. D. Taylor, M. Telen, T. A. Thornton, R. P. Tracy, D. J. Van Den Berg, R. S. Vasan, K. A. Viaud-Martinez, S. Vrieze, D. E. Weeks, B. S. Weir, S. T. Weiss, L.-C. Weng, C. J. Willer, Y. Zhang, X. Zhao, D. K. Arnett, A. E. Ashley-Koch, K. C. Barnes, E. Boerwinkle, S. Gabriel, R. Gibbs, K. M. Rice, S. S. Rich, E. K. Silverman, P. Qasba, W. Gan, NHLBI Trans-Omics for Precision Medicine (TOPMed) Consortium; G. J. Papanicolaou, D. A. Nickerson, S. R. Browning, M. C. Zody, S. Zöllner, J. G. Wilson, L. A. Cupples, C. C. Laurie, C. E. Jaquish, R. D. Hernandez, Timothy D O'Connor, G. R. Abecasis, Sequencing of 53,831 diverse genomes from the NHLBI TOPMed Program. *Nature* **590**, 290–299 (2021).

20. GenomeAsia100K Consortium, The GenomeAsia 100K Project enables genetic discoveries across Asia. *Nature* **576**, 106–111 (2019).

21. D. Wu, J. Dou, X. Chai, C. Bellis, A. Wilm, C. C. Shih, W. W. J. Soon, N. Bertin, C. B. Lin, C. C. Khor, M. DeGiorgio, S. Cheng, L. Bao, N. Karnani, W. Y. K. Hwang, S. Davila, P. Tan, A. Shabbir, A. Moh, E.-K. Tan, J. N. Foo, L. L. Goh, K. P. Leong, R. S. Y. Foo, C. S. P. Lam, A. M. Richards, C.-Y. Cheng, T. Aung, T. Y. Wong, H. H. Ng, J. Liu, C. Wang, M. A. Ackers-Johnson, E. Aliwarga, K. H. K. Ban, D. Bertrand, J. C. Chambers, D. L. H. Chan, C. X. L. Chan, M. L. Chee, M. L. Chee, P. Chen, Y. Chen, E. G. Y. Chew, W. J. Chew, L. H. Y. Chiam, J. P. C. Chong, I. Chua, S. A. Cook, W. Dai, R. Dorajoo, C.-S. Foo, R. S. M. Goh, A. M. Hillmer, I. D. Irwan, F. Jaufeerally, A. Javed, J. Jeyakani, J. T.

H. Koh, J. Y. Koh, P. Krishnaswamy, J. L. Kuan, N. Kumari, A. S. Lee, S. E. Lee, S. Lee, Y. L. Lee, S. T. Leong, Z. Li, P. Y. Li, J. X. Liew, O. W. Liew, S. C. Lim, W. K. Lim, C. W. Lim, T. B. Lim, C. K. Lim, S. Y. Loh, A. W. Lok, C. W. L. Chin, S. Majithia, S. Maurer-Stroh, W. Y. Meah, S. Q. Mok, N. Nargarajan, P. Ng, S. B. Ng, Z. Ng, J. Y. X. Ng, E. Ng, S. L. Ng, S. Nusinovici, C. T. Ong, B. Pan, V. Pedergnana, S. Poh, S. Prabhakar, K. M. Prakash, I. Quek, C. Sabanayagam, W. Q. See, Y. Y. Sia, X. Sim, W. C. Sim, J. So, D. K. N. Soon, E. S. Tai, N. Y. Tan, L. C. S. Tan, H. C. Tan, W. L. W. Tan, M. Tandiono, A. Tay, S. Thakur, Y. C. Tham, Z. Tiang, G. L.-X. Toh, P. K. Tsai, L. Veeravalli, C. S. Verma, L. Wang, M. R. Wang, W.-C. Wong, Z. Xie, K. K. Yeo, L. Zhang, W. Zhai, Y. Zhao, Large-Scale whole-genome sequencing of three diverse asian populations in singapore. *Cell* **179**, 736–749.e15 (2019).

22. Y. Cao, L. Li, M. Xu, Z. Feng, X. Sun, J. Lu, Y. Xu, P. Du, T. Wang, R. Hu, Z. Ye, L. Shi, X. Tang, L. Yan, Z. Gao, G. Chen, Y. Zhang, L. Chen, G. Ning, Y. Bi, W. Wang, The China MAP Consortium; The ChinaMAP analytics of deep whole genome sequences in 10,588 individuals. *Cell Res.* **30**, 717–731 (2020).

23. P.-K. Cong, W.-Y. Bai, J.-C. Li, M.-Y. Yang, S. Khederzadeh, S.-R. Gai, N. Li, Y.-H. Liu, S.-H. Yu, W.-W. Zhao, J.-Q. Liu, Y. Sun, X.-W. Zhu, P.-P. Zhao, J.-W. Xia, P.-L. Guan, Y. Qian, J.-G. Tao, L. Xu, G. Tian, P.-Y. Wang, S.-Y. Xie, M.-C. Qiu, K.-Q. Liu, B.-S. Tang, H.-F. Zheng, Genomic analyses of 10,376 individuals in the Westlake BioBank for Chinese (WBBC) pilot project. *Nat. Commun.* **13**, 2939 (2022).

24. S. Kuriyama, N. Yaegashi, F. Nagami, T. Arai, Y. Kawaguchi, N. Osumi, M. Sakaida, Y. Suzuki, K. Nakayama, H. Hashizume, G. Tamiya, H. Kawame, K. Suzuki, A. Hozawa, N. Nakaya, M. Kikuya, H. Metoki, I. Tsuji, N. Fuse, H. Kiyomoto, J. Sugawara, A. Tsuboi, S. Egawa, K. Ito, K. Chida, T. Ishii, H. Tomita, Y. Taki, N. Minegishi, N. Ishii, J. Yasuda, K. Igarashi, R. Shimizu, M. Nagasaki, S. Koshiba, K. Kinoshita, S. Ogishima, T. Takai-Igarashi, T. Tominaga, O. Tanabe, N. Ohuchi, T. Shimosegawa, S. Kure, H. Tanaka, S. Ito, J. Hitomi, K. Tanno, M. Nakamura, K. Ogasawara, S. Kobayashi, K. Sakata, M. Satoh, A. Shimizu, M. Sasaki, R. Endo, K. Sobue, The Tohoku Medical Megabank Project Study Group, M. Yamamoto, The Tohoku Medical Megabank Project: Design and mission. *J. Epidemiol.* **26**, 493–511 (2016).

25. M. Nagasaki, J. Yasuda, F. Katsuoka, N. Nariai, K. Kojima, Y. Kawai, Y. Yamaguchi-Kabata, J. Yokozawa, I. Danjoh, S. Saito, Y. Sato, T. Mimori, K. Tsuda, R. Saito, X. Pan, S. Nishikawa, S. Ito, Y. Kuroki, O. Tanabe, N. Fuse, S. Kuriyama, H. Kiyomoto, A. Hozawa, N. Minegishi, J. D. Engel, K. Kinoshita, S. Kure, N. Yaegashi, To MMo Japanese Reference Panel Project; M. Yamamoto, Rare variant discovery by deep whole-genome sequencing of 1,070 Japanese individuals. *Nat. Commun.* **6**, 8018 (2015).

26. S. Tadaka, F. Katsuoka, M. Ueki, K. Kojima, S. Makino, S. Saito, A. Otsuki, C. Gocho, M. Sakurai-Yageta, I. Danjoh, I. N. Motoike, Y. Yamaguchi-Kabata, M. Shirota, S. Koshiba, M. Nagasaki, N. Minegishi, A. Hozawa, S. Kuriyama, A. Shimizu, J. Yasuda, N. Fuse, Tohoku Medical Megabank Project Study Group, G. Tamiya, M. Yamamoto, K. Kinoshita, 3.5KJPNv2: An allele frequency panel of 3552 Japanese individuals including the X chromosome. *Hum. Genome Var.* **6**, 28 (2019).

27. S. Tadaka, E. Hishinuma, S. Komaki, I. N. Motoike, J. Kawashima, D. Saigusa, J. Inoue, J. Takayama, Y. Okamura, Y. Aoki, M. Shirota, A. Otsuki, F. Katsuoka, A. Shimizu, G. Tamiya, S. Koshiba, M. Sasaki, M. Yamamoto, K. Kinoshita, jMorp updates in 2020: Large enhancement of multi-omics data resources on the general Japanese population. *Nucleic Acids Res.* **49**, D536–D544 (2021).

28. N. Mitsuhashi, L. Toyo-oka, T. Katayama, M. Kawashima, S. Kawashima, K. Miyazaki, T. Takagi, TogoVar: A comprehensive Japanese genetic variation database. *Hum. Genome Var.* **9**, 44 (2022).

29. Y. Kawai, Y. Watanabe, Y. Omae, R. Miyahara, S.-S. Khor, E. Noiri, K. Kitajima, H. Shimanuki, H. Gatanaga, K. Hata, K. Hattori, A. Iida, H. Ishibashi-Ueda, T. Kaname, T. Kanto, R. Matsumura, K. Miyo, M. Noguchi, K. Ozaki, M. Sugiyama, A. Takahashi, H. Tokuda, T. Tomita, A. Umezawa, H. Watanabe, S. Yoshida, Y. Goto, Y. Maruoka, Y. Matsubara, S. Niida, M. Mizokami, K. Tokunaga, Exploring the genetic diversity of the Japanese population: Insights from a large-scale whole genome sequencing analysis. *PLOS Genet.* **19**, e1010625 (2023).

30. S. Koyama, K. Ito, C. Terao, M. Akiyama, M. Horikoshi, Y. Momozawa, H. Matsunaga, H. Ieki, K. Ozaki, Y. Onouchi, A. Takahashi, S. Nomura, H. Morita, H. Akazawa, C. Kim, J. Seo, K. Higasa, M. Iwasaki, T. Yamaji, N. Sawada, S. Tsugane, T. Koyama, H. Ikezaki, N. Takashima, K. Tanaka, K. Arisawa, K. Kuriki, M. Naito, K. Wakai, S. Suna, Y. Sakata, H. Sato, M. Hori, Y. Sakata, K. Matsuda, Y. Murakami, H. Aburatani, M. Kubo, F. Matsuda, Y. Kamatani, I. Komuro, Population-specific and

trans-ancestry genome-wide analyses identify distinct and shared genetic risk loci for coronary artery disease. *Nat. Genet.* **52**, 1169–1177 (2020).

31. C. Terao, A. Suzuki, Y. Momozawa, M. Akiyama, K. Ishigaki, K. Yamamoto, K. Matsuda, Y. Murakami, S. A. McCarroll, M. Kubo, P.-R. Loh, Y. Kamatani, Chromosomal alterations among age-related haematopoietic clones in Japan. *Nature* **584**, 130–135 (2020).

32. A. Nagai, M. Hirata, Y. Kamatani, K. Muto, K. Matsuda, Y. Kiyohara, T. Ninomiya, A. Tamakoshi, Z. Yamagata, T. Mushiroda, Y. Murakami, K. Yuji, Y. Furukawa, H. Zembutsu, T. Tanaka, Y. Ohnishi, Y. Nakamura, M. Kubo, M. Shiono, K. Misumi, R. Kaieda, H. Harada, S. Minami, M. Emi, N. Emoto, H. Daida, K. Miyauchi, A. Murakami, S. Asai, M. Moriyama, Y. Takahashi, T. Fujioka, W. Obara, S. Mori, H. Ito, S. Nagayama, Y. Miki, A. Masumoto, A. Yamada, Y. Nishizawa, K. Kodama, H. Kutsumi, Y. Sugimoto, Y. Koretsune, H. Kusuoka, H. Yanai, Overview of the BioBank Japan Project: Study design and profile. *J. Epidemiol.* **27**, S2–S8 (2017).

33. Y. Yamaguchi-Kabata, K. Nakazono, A. Takahashi, S. Saito, N. Hosono, M. Kubo, Y. Nakamura, N. Kamatani, Japanese population structure, based on SNP genotypes from 7003 individuals compared to other ethnic groups: Effects on population-based association studies. *Am. J. Hum. Genet.* **83**, 445–456 (2008).

34. Y. Watanabe, M. Isshiki, J. Ohashi, Prefecture-level population structure of the Japanese based on SNP genotypes of 11,069 individuals. *J. Hum. Genet.* **66**, 431–437 (2021).

35. S. Sakaue, J. Hirata, M. Kanai, K. Suzuki, M. Akiyama, C. Lai Too, T. Arayssi, M. Hammoudeh, S. Al Emadi, B. K. Masri, H. Halabi, H. Badsha, I. W. Uthman, R. Saxena, L. Padyukov, M. Hirata, K. Matsuda, Y. Murakami, Y. Kamatani, Y. Okada, Dimensionality reduction reveals fine-scale structure in the Japanese population with consequences for polygenic risk prediction. *Nat. Commun.* **11**, 1569 (2020).

36. K. J. Karczewski, L. C. Francioli, G. Tiao, B. B. Cummings, J. Alföldi, Q. Wang, R. L. Collins, K. M. Laricchia, A. Ganna, D. P. Birnbaum, L. D. Gauthier, H. Brand, M. Solomonson, N. A. Watts, D. Rhodes, M. Singer-Berk, E. M. England, E. G. Seaby, J. A. Kosmicki, R. K. Walters, K. Tashman, Y. Farjoun, E. Banks, T. Poterba, A. Wang, C. Seed, N. Whiffin, J. X. Chong, K. E. Samocha, E. Pierce-

Hoffman, Z. Zappala, Anne H O'Donnell-Luria, E. V. Minikel, B. Weisburd, M. Lek, J. S. Ware, C. Vittal, I. M. Armean, L. Bergelson, K. Cibulskis, K. M. Connolly, M. Covarrubias, S. Donnelly, S. Ferriera, S. Gabriel, J. Gentry, N. Gupta, T. Jeandet, D. Kaplan, C. Llanwarne, R. Munshi, S. Novod, N. Petrillo, D. Roazen, V. Ruano-Rubio, A. Saltzman, M. Schleicher, J. Soto, K. Tibbetts, C. Tolonen, G. Wade, M. E. Talkowski, Genome Aggregation Database Consortium; B. M. Neale, M. J. Daly, D. G. MacArthur, The mutational constraint spectrum quantified from variation in 141,456 humans. *Nature* **581**, 434–443 (2020).

37. Japanese Archipelago Human Population Genetics Consortium; The history of human populations in the Japanese Archipelago inferred from genome-wide SNP data with a special reference to the Ainu and the Ryukyuan populations. *J. Hum. Genet.* **57**, 787–795 (2012).

38. Y.-L. Li, J.-X. Liu, StructureSelector: A web-based software to select and visualize the optimal number of clusters using multiple methods. *Mol. Ecol. Resour.* **18**, 176–177 (2018).

39. D. J. Lawson, L. van Dorp, D. Falush, A tutorial on how not to over-interpret STRUCTURE and ADMIXTURE bar plots. *Nat. Commun.* **9**, 3258 (2018).

40. T. Jinam, Y. Kawai, Y. Kamatani, S. Sonoda, K. Makisumi, H. Sameshima, K. Tokunaga, N. Saitou, Genome-wide SNP data of Izumo and Makurazaki populations support inner-dual structure model for origin of Yamato people. *J. Hum. Genet.*, **66**, 681–687 (2021).

41. N. P. Cooke, V. Mattiangeli, L. M. Cassidy, K. Okazaki, C. A. Stokes, S. Onbe, S. Hatakeyama, K. Machida, K. Kasai, N. Tomioka, A. Matsumoto, M. Ito, Y. Kojima, D. G. Bradley, T. Gakuhari, S. Nakagome, Ancient genomics reveals tripartite origins of Japanese populations. *Sci. Adv.* **7,** eabh2419 (2021).

42. M. Robbeets, R. Bouckaert, M. Conte, A. Savelyev, T. Li, D.-I. An, K. Shinoda, Y. Cui, T. Kawashima, G. Kim, J. Uchiyama, J. Dolińska, S. Oskolskaya, K.-Y. Yamano, N. Seguchi, H. Tomita, H. Takamiya, H. Kanzawa-Kiriyama, H. Oota, H. Ishida, R. Kimura, T. Sato, J.-H. Kim, B. Deng, R. Bjørn, S. Rhee, K.-D. Ahn, I. Gruntov, O. Mazo, J. R. Bentley, R. Fernandes, P. Roberts, I. R. Bausch, L. Gilaizeau, M. Yoneda, M. Kugai, R. A. Bianco, F. Zhang, M. Himmel, M. J. Hudson, C. Ning, Triangulation supports agricultural spread of the Transeurasian languages. *Nature* **599**, 616–621 (2021).

43. H. Kanzawa-Kiriyama, T. A. Jinam, Y. Kawai, T. Sato, K. Hosomichi, A. Tajima, N. Adachi, H. Matsumura, K. Kryukov, N. Saitou, K.-I. Shinoda, Late Jomon male and female genome sequences from the Funadomari site in Hokkaido, Japan, *Anthropol. Sci.* **127**, 83–108 (2019).

44. T. Gakuhari, S. Nakagome, S. Rasmussen, M. E. Allentoft, T. Sato, T. Korneliussen, B. N. Chuinneagáin, H. Matsumae, K. Koganebuchi, R. Schmidt, S. Mizushima, O. Kondo, N. Shigehara, M. Yoneda, R. Kimura, H. Ishida, T. Masuyama, Y. Yamada, A. Tajima, H. Shibata, A. Toyoda, T. Tsurumoto, T. Wakebe, H. Shitara, T. Hanihara, E. Willerslev, M. Sikora, H. Oota, Ancient Jomon genome sequence analysis sheds light on migration patterns of early East Asian populations. *Commun. Biol.* **3**, 437 (2020).

45. C. Ning, T. Li, K. Wang, F. Zhang, T. Li, X. Wu, S. Gao, Q. Zhang, H. Zhang, M. J. Hudson, G. Dong, S. Wu, Y. Fang, C. Liu, C. Feng, W. Li, T. Han, R. Li, J. Wei, Y. Zhu, Y. Zhou, C.-C. Wang, S. Fan, Z. Xiong, Z. Sun, M. Ye, L. Sun, X. Wu, F. Liang, Y. Cao, X. Wei, H. Zhu, H. Zhou, J. Krause, M. Robbeets, C. Jeong, Y. Cui, Ancient genomes from northern China suggest links between subsistence changes and human migration. *Nat. Commun.* **11**, 2700 (2020).

46. C.-C. Wang, H.-Y. Yeh, A. N. Popov, H.-Q. Zhang, H. Matsumura, K. Sirak, O. Cheronet, A. Kovalev, N. Rohland, A. M. Kim, S. Mallick, R. Bernardos, D. Tumen, J. Zhao, Y.-C. Liu, J.-Y. Liu, M. Mah, K. Wang, Z. Zhang, N. Adamski, N. Broomandkhoshbacht, K. Callan, F. Candilio, Kellie Sara Duffett Carlson, B. J. Culleton, L. Eccles, S. Freilich, D. Keating, A. M. Lawson, K. Mandl, M. Michel, J. Oppenheimer, K. T. Özdoğan, K. Stewardson, S. Wen, S. Yan, F. Zalzala, R. Chuang, C.-J. Huang, H. Looh, C.-C. Shiung, Y. G. Nikitin, A. V. Tabarev, A. A. Tishkin, S. Lin, Z.-Y. Sun, X.-M. Wu, T.-L. Yang, X. Hu, L. Chen, H. Du, J. Bayarsaikhan, E. Mijiddorj, D. Erdenebaatar, T.-O. Iderkhangai, E. Myagmar, H. Kanzawa-Kiriyama, M. Nishino, K.-I. Shinoda, O. A. Shubina, J. Guo, W. Cai, Q. Deng, L. Kang, D. Li, D. Li, R. Lin, Nini, R. Shrestha, L.-X. Wang, L. Wei, G. Xie, H. Yao, M. Zhang, G. He, X. Yang, R. Hu, M. Robbeets, S. Schiffels, D. J. Kennett, L. Jin, H. Li, J. Krause, R. Pinhasi, D. Reich, Genomic insights into the formation of human populations in East Asia. *Nature* **591**, 413–419 (2021).

47. P. Gelabert, A. Blazyte, Y. Chang, D. M. Fernandes, S. Jeon, J. G. Hong, J. Yoon, Y. Ko, V. Oberreiter, O. Cheronet, K. T. Özdoğan, S. Sawyer, S. Yang, E. M. Greytak, H. Choi, J. Kim, J.-I. Kim,

C. Jeong, K. Bae, J. Bhak, R. Pinhasi, Northeastern Asian and Jomon-related genetic structure in the Three Kingdoms period of Gimhae, Korea, *Curr. Biol.* **32**, 3232–3244.e6 (2022).

48. N. P. Cooke, V. Mattiangeli, L. M. Cassidy, K. Okazaki, K. Kasai, D. G. Bradley, T. Gakuhari, S. Nakagome, Genomic insights into a tripartite ancestry in the Southern Ryukyu Islands. *Evol. Hum. Sci.* **5**, e23 (2023).

49. M. Sekine, H. Nagata, S. Tsuji, Y. Hirai, S. Fujimoto, M. Hatae, I. Kobayashi, T. Fujii, I. Nagata, K. Ushijima, K. Obata, M. Suzuki, M. Yoshinaga, N. Umesaki, S. Satoh, T. Enomoto, S. Motoyama, K. Tanaka, Japanese Familial Ovarian Cancer Study Group; Mutational analysis of BRCA1 and BRCA2 and clinicopathologic analysis of ovarian cancer in 82 ovarian cancer families: Two common founder mutations of BRCA1 in Japanese population. *Clin. Cancer Res.* **7**, 3144–3150 (2001).

50. Y. C. Kim, L. Zhao, H. Zhang, Y. Huang, J. Cui, F. Xiao, B. Downs, S. M. Wang, Prevalence and spectrum of BRCA germline variants in mainland Chinese familial breast and ovarian cancer patients. *Oncotarget* **7**, 9600–9612 (2016).

51. H. Kim, D.-Y. Cho, D. H. Choi, S.-Y. Choi, I. Shin, W. Park, S. J. Huh, S.-H. Han, M. H. Lee, S. H. Ahn, B. H. Son, S.-W. Kim, Korean Breast Cancer Study Group, B. G. Haffty, Characteristics and spectrum of BRCA1 and BRCA2 mutations in 3,922 Korean patients with breast and ovarian cancer. *Breast Cancer Res. Treat.* **134**, 1315–1326 (2012).

52. Y. Momozawa, R. Sasai, Y. Usui, K. Shiraishi, Y. Iwasaki, Y. Taniyama, M. T. Parsons, K. Mizukami, Y. Sekine, M. Hirata, Y. Kamatani, M. Endo, C. Inai, S. Takata, H. Ito, T. Kohno, K. Matsuda, S. Nakamura, K. Sugano, T. Yoshida, H. Nakagawa, K. Matsuo, Y. Murakami, A. B. Spurdle, M. Kubo, Expansion of cancer risk profile for BRCA1 and BRCA2 pathogenic variants. *JAMA Oncol.* **8**, 871 (2022), 878.

53. K. E. Samocha, E. B. Robinson, S. J. Sanders, C. Stevens, A. Sabo, L. M. McGrath, J. A. Kosmicki, K. Rehnström, S. Mallick, A. Kirby, D. P. Wall, D. G. MacArthur, S. B. Gabriel, M. DePristo, S. M. Purcell, A. Palotie, E. Boerwinkle, J. D. Buxbaum, E. H. Cook, R. A. Gibbs, G. D. Schellenberg, J. S. Sutcliffe, B. Devlin, K. Roeder, B. M. Neale, M. J. Daly, A framework for the interpretation of de novo mutation in human disease. *Nat. Genet.* **46**, 944–950 (2014).

54. L. Sundaram, H. Gao, S. R. Padigepati, J. F. McRae, Y. Li, J. A. Kosmicki, N. Fritzilas, J. Hakenberg, A. Dutta, J. Shon, J. Xu, S. Batzoglou, X. Li, K. K.-H. Farh, Predicting the clinical impact of human mutation with deep neural networks. *Nat. Genet.* **50**, 1161–1170 (2018).

55. C. C. Paulusma, M. Kool, P. J. Bosma, G. L. Scheffer, F. Ter Borg, R. J. Scheper, G. N. Tytgat, P. Borst, F. Baas, R. O. Elferink, A mutation in the human canalicular multispecific organic anion transporter gene causes the Dubin-Johnson syndrome. *Hepatology* **25**, 1539–1542 (1997).

56. K. Hashimoto, T. Uchiumi, T. Konno, T. Ebihara, T. Nakamura, M. Wada, S. Sakisaka, F. Maniwa, T. Amachi, K. Ueda, M. Kuwano, Trafficking and functional defects by mutations of the ATP-binding domains in MRP2 in patients with Dubin-Johnson syndrome. *Hepatology* **36**, 1236–1245 (2002).

57. R. L. Snoeckx, P. L. M. Huygen, D. Feldmann, S. Marlin, F. Denoyelle, J. Waligora, M. Mueller-Malesinska, A. Pollak, R. Ploski, A. Murgia, E. Orzan, P. Castorina, U. Ambrosetti, E. Nowakowska-Szyrwinska, J. Bal, W. Wiszniewski, A. R. Janecke, D. Nekahm-Heis, P. Seeman, O. Bendova, M. A. Kenna, A. Frangulov, H. L. Rehm, M. Tekin, A. Incesulu, H.-H. M. Dahl, D. du Sart, L. Jenkins, D. Lucas, M. Bitner-Glindzicz, K. B. Avraham, Z. Brownstein, I. del Castillo, F. Moreno, N. Blin, M. Pfister, I. Sziklai, T. Toth, P. M. Kelley, E. S. Cohn, L. Van Maldergem, P. Hilbert, A.-F. Roux, M. Mondain, L. H. Hoefsloot, C. W. R. J. Cremers, T. Löppönen, H. Löppönen, A. Parving, K. Gronskov, I. Schrijver, J. Roberson, F. Gualandi, A. Martini, G. Lina-Granade, N. Pallares-Ruiz, C. Correia, G. Fialho, K. Cryns, N. Hilgert, P. Van de Heyning, C. J. Nishimura, R. J. H. Smith, G. Van Camp, GJB2 mutations and degree of hearing loss: A multicenter study. *Am. J. Hum. Genet.* **77**, 945–957 (2005).

58. A. W. Stoker, Receptor tyrosine phosphatases in axon growth and guidance. *Curr. Opin. Neurobiol.* **11**, 95–102 (2001).

59. N. Choucair, C. Mignon-Ravix, P. Cacciagli, J. Abou Ghoch, A. Fawaz, A. Mégarbané, L. Villard, E. Chouery, Evidence that homozygous PTPRD gene microdeletion causes trigonocephaly, hearing loss, and intellectual disability. *Mol. Cytogenet.* **8**, 39 (2015).

60. T. Groza, F. L. Gomez, H. H. Mashhadi, V. Muñoz-Fuentes, O. Gunes, R. Wilson, P. Cacheiro, A. Frost, P. Keskivali-Bond, B. Vardal, A. McCoy, T. K. Cheng, L. Santos, S. Wells, D. Smedley, A.-M.

Mallon, H. Parkinson, The International Mouse Phenotyping Consortium: Comprehensive knockout phenotyping underpinning the study of human disease. *Nucleic Acids Res.* **51**, D1038–D1045 (2023).

61. P. Qin, M. Stoneking, Denisovan ancestry in East Eurasian and Native American populations. *Mol. Biol. Evol.* **32**, 2665–2674 (2015).

62. S. R. Browning, B. L. Browning, Y. Zhou, S. Tucci, J. M. Akey, Analysis of human sequence data reveals two pulses of archaic Denisovan admixture. *Cell* **173**, 53–61.e9 (2018).

63. L. Chen, A. B. Wolf, W. Fu, L. Li, J. M. Akey, Identifying and interpreting apparent Neanderthal ancestry in African individuals. *Cell* **180**, 677–687.e16 (2020).

64. M. Larena, J. McKenna, F. Sanchez-Quinto, C. Bernhardsson, C. Ebeo, R. Reyes, O. Casel, J.-Y. Huang, K. P. Hagada, D. Guilay, J. Reyes, F. P. Allian, V. Mori, L. S. Azarcon, A. Manera, C. Terando, L. Jamero, G. Sireg, R. Manginsay-Tremedal, M. S. Labos, R. D. Vilar, A. Latiph, R. L. Saway, E. Marte, P. Magbanua, A. Morales, I. Java, R. Reveche, B. Barrios, E. Burton, J. C. Salon, Ma. J. T. Kels, A. Albano, R. B. Cruz-Angeles, E. Molanida, L. Granehäll, M. Vicente, H. Edlund, J.-H. Loo, J. Trejaut, S. Y. W. Ho, L. Reid, K. Lambeck, H. Malmström, C. Schlebusch, P. Endicott, M. Jakobsson, Philippine Ayta possess the highest level of Denisovan ancestry in the world. *Curr. Biol.* **31**, 4219–4230.e10 (2021).

65. M. Dannemann, The population-specific impact of Neandertal introgression on human disease. *Genome Biol. Evol.* **13**, evaa250 (2021).

66. N. Tanaka, M. Koido, A. Suzuki, N. Otomo, H. Suetsugu, Y. Kochi, K. Tomizuka, Y. Momozawa, Y. Kamatani, Biobank. Japan. Project; S. Ikegawa, K. Yamamoto, C. Terao, Eight novel susceptibility loci and putative causal variants in atopic dermatitis. *J. Allergy Clin. Immunol.*, **148**, 12931306 (2021).

67. M. Koido, C.-C. Hon, S. Koyama, H. Kawaji, Y. Murakawa, K. Ishigaki, K. Ito, J. Sese, N. F. Parrish, Y. Kamatani, P. Carninci, C. Terao, Prediction of the cell-type-specific transcription of non-coding RNAs from genome sequences via machine learning. *Nat. Biomed. Eng.* **7**, 830–844 (2023).

68. K. Suzuki, M. Akiyama, K. Ishigaki, M. Kanai, J. Hosoe, N. Shojima, A. Hozawa, A. Kadota, K. Kuriki, M. Naito, K. Tanno, Y. Ishigaki, M. Hirata, K. Matsuda, N. Iwata, M. Ikeda, N. Sawada, T.

Yamaji, M. Iwasaki, S. Ikegawa, S. Maeda, Y. Murakami, K. Wakai, S. Tsugane, M. Sasaki, M. Yamamoto, Y. Okada, M. Kubo, Y. Kamatani, M. Horikoshi, T. Yamauchi, T. Kadowaki, Identification of 28 new susceptibility loci for type 2 diabetes in the Japanese population. *Nat. Genet.* **51**, 379–386 (2019).

69. L. Skov, M. Coll Macià, G. Sveinbjörnsson, F. Mafessoni, E. A. Lucotte, M. S. Einarsdóttir, H. Jonsson, B. Halldorsson, D. F. Gudbjartsson, A. Helgason, M. H. Schierup, K. Stefansson, The nature of Neanderthal introgression revealed by 27,566 Icelandic genomes. *Nature* **582**, 78–83 (2020).

70. H. Zeberg, S. Pääbo, The major genetic risk factor for severe COVID-19 is inherited from Neanderthals. *Nature* **587**, 610–612 (2020).

71. B. F. Voight, S. Kudaravalli, X. Wen, J. K. Pritchard, A map of recent positive selection in the human genome. *PLoS Biol.* **4**, e72 (2006).

72. P. F. Palamara, J. Terhorst, Y. S. Song, A. L. Price, High-throughput inference of pairwise coalescence times identifies signals of selection and enriched disease heritability. *Nat. Genet.* **50**, 1311–1317 (2018).

73. Y. Okada, Y. Momozawa, S. Sakaue, M. Kanai, K. Ishigaki, M. Akiyama, T. Kishikawa, Y. Arai, T. Sasaki, K. Kosaki, M. Suematsu, K. Matsuda, K. Yamamoto, M. Kubo, N. Hirose, Y. Kamatani, Deep whole-genome sequencing reveals recent selection signatures linked to evolution and disease risk of Japanese. *Nat. Commun.* **9**, 1631 (2018).

74. X. Liu, M. Matsunami, M. Horikoshi, S. Ito, Y. Ishikawa, K. Suzuki, Y. Momozawa, S. Niida, R. Kimura, K. Ozaki, S. Maeda, M. Imamura, C. Terao, Natural selection signatures in the Hondo and Ryukyu Japanese subpopulations. *Mol. Biol. Evol.* **40**, msad231 (2023).

75. K. Hanihara, Dual structure model for the population history of the Japanese. *Jpn. Rev.* **2**, 1–33 (1991).

76. M. J. Hudson, S. Nakagome, J. B. Whitman, The evolving Japanese: The dual structure hypothesis at 30. *Evol. Hum. Sci.* **2**, e6 (2020).

77. N. Osada, Y. Kawai, Exploring models of human migration to the Japanese archipelago using genome-wide genetic data. *Anthropol. Sci.* **129**, 45–58 (2021).

78. T. A. Jinam, Y. Kawai, N. Saitou, Modern human DNA analyses with special reference to the inner dual-structure model of Yaponesian. *Anthropol. Sci.* **129**, 3–11 (2021).

79. K. Mizoguchi, *The Archaeology of Japan: From the Earliest Rice Farming Villages to the Rise of the State* (Cambridge University Press, ed. 1, 2013).

80. G. Barnes, *State Formation in Japan: Emergence of a 4th-Century Ruling Elite* (Routledge, 2007).

81. C.-H. Huang, D. C. Kang, State formation in Korea and Japan, 400–800 CE: Emulation and learning, not bellicist competition. *Int. Organ.* **76**, 1–31 (2022).

82. K. F. Friday, Pushing beyond the Pale: The Yamato Conquest of the Emishi and Northern Japan. *J. Jpn. Stud.* **23**, 1–24 (1997).

83. K. Hanihara, Emishi, Ezo and Ainu: An anthropological perspective. *Jpn. Rev.* **1**, 35–48 (1990).

84. H. Matsumura, Y. Dodo, Dental characteristics of Tohoku residents in Japan: Implications for biological affinity with ancient Emishi. *Anthropol. Sci.* **117**, 95–105 (2009).

85. E. De Boer, M. A. Yang, A. Kawagoe, G. L. Barnes, Japan considered from the hypothesis of farmer/language spread. *Evol. Hum. Sci.* **2**, e13 (2020).

86. E. R. Crema, C. J. Stevens, S. Shoda, Bayesian analyses of direct radiocarbon dates reveal geographic variations in the rate of rice farming dispersal in prehistoric Japan. *Sci. Adv.* **8**, eadc9171 (2022).

87. E. Endo, C. Leipe, The onset, dispersal and crop preferences of early agriculture in the Japanese archipelago as derived from seed impressions in pottery. *Quat. Int.* **623**, 35–49 (2022).

88. B. B. Cummings, K. J. Karczewski, J. A. Kosmicki, E. G. Seaby, N. A. Watts, M. Singer-Berk, J. M. Mudge, J. Karjalainen, F. K. Satterstrom, A. H. O'Donnell-Luria, T. Poterba, C. Seed, M. Solomonson, J. Alföldi, M. J. Daly, D. G. MacArthur, Transcript expression-aware annotation improves rare variant interpretation. *Nature* **581**, 452–458 (2020).

89. E. Huerta-Sánchez, X. Jin, Asan, Z. Bianba, B. M. Peter, N. Vinckenbosch, Y. Liang, X. Yi, M. He, M. Somel, P. Ni, B. Wang, X. Ou, Huasang, J. Luosang, Z. X. P. Cuo, K. Li, G. Gao, Y. Yin, W. Wang, X.

Zhang, X. Xu, H. Yang, Y. Li, J. Wang, J. Wang, R. Nielsen, Altitude adaptation in Tibetans caused by introgression of Denisovan-like DNA. *Nature* **512**, 194–197 (2014).

90. M. Dannemann, J. Kelso, The contribution of neanderthals to phenotypic variation in modern humans. *Am. J. Hum. Genet.* **101**, 578–589 (2017).

91. L. B. Knudsen, J. Lau, The discovery and development of liraglutide and semaglutide. *Front. Endocrinol.* **10**, 155 (2019).

92. Y. Yasumizu, S. Sakaue, T. Konuma, K. Suzuki, K. Matsuda, Y. Murakami, M. Kubo, P. F. Palamara, Y. Kamatani, Y. Okada, Genome-wide natural selection signatures are linked to genetic risk of modern phenotypes in the Japanese population. *Mol. Biol. Evol.* **37**, 1306–1316 (2020).

93. P.-R. Loh, P. F. Palamara, A. L. Price, Fast and accurate long-range phasing in a UK Biobank cohort. *Nat. Genet.* **48**, 811–816 (2016).

94. D. H. Alexander, J. Novembre, K. Lange, Fast model-based estimation of ancestry in unrelated individuals. *Genome Res.* **19**, 1655–1664 (2009).

95. M. Petr, B. Vernot, J. Kelso, admixr - R package for reproducible analyses using ADMIXTOOLS. *Bioinformatics* **35**, 3194–3195 (2019).

96. M. A. Yang, X. Fan, B. Sun, C. Chen, J. Lang, Y.-C. Ko, C. Tsang, H. Chiu, T. Wang, Q. Bao, X. Wu, M. Hajdinjak, A. M.-S. Ko, M. Ding, P. Cao, R. Yang, F. Liu, B. Nickel, Q. Dai, X. Feng, L. Zhang, C. Sun, C. Ning, W. Zeng, Y. Zhao, M. Zhang, X. Gao, Y. Cui, D. Reich, M. Stoneking, Q. Fu, Ancient DNA indicates human population shifts and admixture in northern and southern China. *Science*, **369** (2020), 282, 288.

97. S. J. Jurgens, S. H. Choi, V. N. Morrill, M. Chaffin, J. P. Pirruccello, J. L. Halford, L.-C. Weng, V. Nauffal, C. Roselli, A. W. Hall, M. T. Oetjens, B. Lagerman, D. P. vanMaanen, Regeneron Genetics Center, G. Abecasis, X. Bai, S. Balasubramanian, A. Baras, C. Beechert, B. Boutkov, M. Cantor, G. Coppola, T. De, A. Deubler, A. Economides, G. Eom, M. A. R. Ferreira, C. Forsythe, E. D. Fuller, Z. Gu, L. Habegger, A. Hawes, M. B. Jones, K. Karalis, S. Khalid, O. Krasheninina, R. Lanche, M. Lattari, D. Li, A. Lopez, L. A. Lotta, K. Manoochehri, A. J. Mansfield, E. K. Maxwell, J. Mighty, L. J. Mitnaul,

M. Nafde, J. Nielsen, S. O'Keeffe, M. Orelus, J. D. Overton, M. S. Padilla, R. Panea, T. Polanco, M. Pradhan, A. Rasool, J. G. Reid, W. Salerno, T. D. Schleicher, A. Shuldiner, K. Siminovitch, J. C. Staples, R. H. Ulloa, N. Verweij, L. Widom, S. E. Wolf, K. G. Aragam, K. L. Lunetta, C. M. Haggerty, S. A. Lubitz, P. T. Ellinor, Analysis of rare genetic variation underlying cardiometabolic diseases and traits among 200,000 individuals in the UK Biobank. *Nat. Genet.* **54**, 240–250 (2022).

98. K. Ishigaki, M. Akiyama, M. Kanai, A. Takahashi, E. Kawakami, H. Sugishita, S. Sakaue, N. Matoba, S.-K. Low, Y. Okada, C. Terao, T. Amariuta, S. Gazal, Y. Kochi, M. Horikoshi, K. Suzuki, K. Ito, S. Koyama, K. Ozaki, S. Niida, Y. Sakata, Y. Sakata, T. Kohno, K. Shiraishi, Y. Momozawa, M. Hirata, K. Matsuda, M. Ikeda, N. Iwata, S. Ikegawa, I. Kou, T. Tanaka, H. Nakagawa, A. Suzuki, T. Hirota, M. Tamari, K. Chayama, D. Miki, M. Mori, S. Nagayama, Y. Daigo, Y. Miki, T. Katagiri, O. Ogawa, W. Obara, H. Ito, T. Yoshida, I. Imoto, T. Takahashi, C. Tanikawa, T. Suzuki, N. Sinozaki, S. Minami, H. Yamaguchi, S. Asai, Y. Takahashi, K. Yamaji, K. Takahashi, T. Fujioka, R. Takata, H. Yanai, A. Masumoto, Y. Koretsune, H. Kutsumi, M. Higashiyama, S. Murayama, N. Minegishi, K. Suzuki, K. Tanno, A. Shimizu, T. Yamaji, M. Iwasaki, N. Sawada, H. Uemura, K. Tanaka, M. Naito, M. Sasaki, K. Wakai, S. Tsugane, M. Yamamoto, K. Yamamoto, Y. Murakami, Y. Nakamura, S. Raychaudhuri, J. Inazawa, T. Yamauchi, T. Kadowaki, M. Kubo, Y. Kamatani, Large-scale genome-wide association study in a Japanese population identifies novel susceptibility loci across different diseases. *Nat. Genet.* **52**, 669–679 (2020).

99. Z. A. Szpiech, R. D. Hernandez, selscan: An efficient multithreaded program to perform EHH-based scans for positive selection. *Mol. Biol. Evol.* **31**, 2824–2827 (2014).

100. J. P. Spence, Y. S. Song, Inference and analysis of population-specific fine-scale recombination maps across 26 diverse human populations. *Sci. Adv.* **5**, eaaw9206 (2019).

101. X. Liu, S. Takata, K. Ashikawa, T. Aoi, S. Kosugi, C. Terao, N. F. Parrish, K. Matsuda, H. Nakagawa, Y. Kamatani, M. Kubo, Y. Momozawa, Prevalence and spectrum of pathogenic germline variants in Japanese patients with early-onset colorectal, breast, and prostate cancer. *JCO Precis. Oncol.*, **4**, 183–191 (2020).

102. S. Ito, X. Liu, Y. Ishikawa, D. D. Conti, N. Otomo, Z. Kote-Jarai, H. Suetsugu, R. A. Eeles, Y. Koike, K. Hikino, S. Yoshino, K. Tomizuka, M. Horikoshi, K. Ito, Y. Uchio, Y. Momozawa, M. Kubo, The

BioBank Japan Project, A. Masumoto, A. Nagai, D. Obata, H. Yamaguchi, K. Muto, K. Takahashi, K. Yamaji, K. Yoshimori, M. Higashiyama, N. Sinozaki, S. Asai, S. Nagayama, S. Murayama, S. Minami, T. Suzuki, T. Morisaki, W. Obara, Y. Takahashi, Y. Furukawa, Y. Murakami, Y. Yamanashi, Y. Koretsune, Y. Kamatani, K. Matsuda, C. A. Haiman, S. Ikegawa, H. Nakagawa, C. Terao, Androgen receptor binding sites enabling genetic prediction of mortality due to prostate cancer in cancer-free subjects. *Nat. Commun.* **14**, 4863 (2023).

103. Y. Ishikawa, N. Tanaka, Y. Asano, M. Kodera, Y. Shirai, M. Akahoshi, M. Hasegawa, T. Matsushita, K. Saito, S. Motegi, H. Yoshifuji, A. Yoshizaki, T. Kohmoto, K. Takagi, A. Oka, M. Kanda, Y. Tanaka, Y. Ito, K. Nakano, H. Kasamatsu, A. Utsunomiya, A. Sekiguchi, H. Niiro, M. Jinnin, K. Makino, T. Makino, H. Ihn, M. Yamamoto, C. Suzuki, H. Takahashi, E. Nishida, A. Morita, T. Yamamoto, M. Fujimoto, Y. Kondo, D. Goto, T. Sumida, N. Ayuzawa, H. Yanagida, T. Horita, T. Atsumi, H. Endo, Y. Shima, A. Kumanogoh, J. Hirata, N. Otomo, H. Suetsugu, Y. Koike, K. Tomizuka, S. Yoshino, X. Liu, S. Ito, K. Hikino, A. Suzuki, Y. Momozawa, S. Ikegawa, Y. Tanaka, O. Ishikawa, K. Takehara, T. Torii, S. Sato, Y. Okada, T. Mimori, F. Matsuda, K. Matsuda, T. Amariuta, I. Imoto, K. Matsuo, M. Kuwana, Y. Kawaguchi, K. Ohmura, C. Terao, GWAS for systemic sclerosis identifies six novel susceptibility loci including one in the Fcγ receptor region. *Nat. Commun.* **15**, 319 (2024).

104. S. Kosugi, Y. Kamatani, K. Harada, K. Tomizuka, Y. Momozawa, T. Morisaki, C. Terao, Detection of trait-associated structural variations using short-read sequencing. *Cell Genomics* **3**, 100328 (2023).

105. A. Diaz-Papkovich, L. Anderson-Trocmé, S. Gravel, A review of UMAP in population genetics. *J. Hum. Genet.* **66**, 85–91 (2021).

106. Q. Liu, D. Wu, C. Wang, Identification of genomic regions distorting population structure inference in diverse continental groups. *Quant. Biol.* **10**, 287 (2022), 298.

107. T. Sato, S. Nakagome, C. Watanabe, K. Yamaguchi, A. Kawaguchi, K. Koganebuchi, K. Haneji, T. Yamaguchi, T. Hanihara, K. Yamamoto, Genome-wide SNP analysis reveals population structure and demographic history of the ryukyu islanders in the southern part of the Japanese archipelago. *Mol. Biol. Evol.* **31**, 2929–2940 (2014).

108. M. Matsunami, K. Koganebuchi, M. Imamura, H. Ishida, R. Kimura, S. Maeda, Fine-scale genetic structure and demographic history in the Miyako Islands of the Ryukyu Archipelago. *Mol. Biol. Evol.* **38**, 2045–2056 (2021).

109. K. Koganebuchi, K. Haneji, T. Toma, K. Joh, H. Soejima, K. Fujimoto, H. Ishida, M. Ogawa, T. Hanihara, S. Harada, S. Kawamura, H. Oota, The allele frequency of *ALDH2*Glu504Lys* and *ADH1B*Arg47His* for the Ryukyu islanders and their history of expansion among East Asians. *Am. J. Hum. Biol.* **29**, e22933 (2017).

110. Y. Watanabe, J. Ohashi, Modern Japanese ancestry-derived variants reveal the formation process of the current Japanese regional gradations. *iScience* **26**, 106130 (2023).

111. A. M. Chiu, E. K. Molloy, Z. Tan, A. Talwalkar, S. Sankararaman, Inferring population structure in biobank-scale genomic data. *Am. J. Hum. Genet.* **109**, 727–737 (2022).

112. X. Zhang, K. E. Witt, M. M. Bañuelos, A. Ko, K. Yuan, S. Xu, R. Nielsen, E. Huerta-Sanchez, The history and evolution of the Denisovan- *EPAS1* haplotype in Tibetans. *Proc. Natl. Acad. Sci.* **118**, e2020803118 (2021).

113. R. Torrance, The infrastructure of the gods: Izumo in the Yayoi and Kofun periods. *Jpn. Rev.* **29**, 3–38 (2016).

114. Y. Zhong, *The Origin of Modern Shinto in Japan: The Vanquished Gods of Izumo* (Bloomsbury Academic, Paperback edition, 2018).

115. H. Oota, A. J. Pakstis, B. Bonne-Tamir, D. Goldman, E. Grigorenko, S. L. B. Kajuna, N. J. Karoma, S. Kungulilo, R.-B. Lu, K. Odunsi, F. Okonofua, O. V. Zhukova, J. R. Kidd, K. K. Kidd, The evolution and population genetics of the ALDH2 locus: Random genetic drift, selection, and low levels of recombination. *Ann. Hum. Genet.* **68**, 93–109 (2004).

116. D. Goldman, M.-A. Enoch, Genetic epidemiology of ethanol metabolic enzymes: A role for selection, in *World Review of Nutrition and Dietetics*, A. P. Simopoulos, B. Childs, Eds. (S. Karger AG, 1990), vol. 63, pp. 143–160.

117. A. Espinosa, L. Yan, Z. Zhang, L. Foster, D. Clark, E. Li, S. L. Stanley, The bifunctional entamoeba histolytica alcohol dehydrogenase 2 (EhADH2) protein is necessary for amebic growth and survival and requires an intact C-terminal domain for both alcohol dehydrogenase and acetaldehyde dehydrogenase activity. *J. Biol. Chem.* **276**, 20136–20143 (2001).

118. E. Strauss, Coenzyme A biosynthesis and enzymology, in *Comprehensive Natural Products II* (Elsevier, 2010), pp. 351–410.

119. A. Azam, M. N. Peerzada, K. Ahmad, Parasitic diarrheal disease: Drug development and targets. *Front. Microbiol.* **6,** 1183 (2015).

120. N. Tran-Thi, R. J. Lowe, J. M. Schurer, T. Vu-Van, L. E. MacDonald, P. Pham-Duc, Turning poop into profit: Cost-effectiveness and soil transmitted helminth infection risk associated with human excreta reuse in Vietnam. *PLoS Negl. Trop. Dis.* **11**, e0006088 (2017).

121. H. Takamiya, N. Nakamura, The beginning of agriculture in the Ryukyu archipelago. *S. Pac. Study* **41**, 1–34 (2021).

122. X. Zhang, A. Sun, J. Ge, Origin and spread of the ALDH2 Glu504Lys allele. *Phenomics* **1**, 222–228 (2021).

123. N. Matoba, M. Akiyama, K. Ishigaki, M. Kanai, A. Takahashi, Y. Momozawa, S. Ikegawa, M. Ikeda, N. Iwata, M. Hirata, K. Matsuda, Y. Murakami, M. Kubo, Y. Kamatani, Y. Okada, GWAS of 165,084 Japanese individuals identified nine loci associated with dietary habits. *Nat. Hum. Behav.* **4**, 308–316 (2020).

124. S. Sakaue, M. Akiyama, M. Hirata, K. Matsuda, Y. Murakami, M. Kubo, Y. Kamatani, Y. Okada, Functional variants in ADH1B and ALDH2 are non-additively associated with all-cause mortality in Japanese population. *Eur. J. Hum. Genet.* **28**, 378–382 (2020).
